# Co-release of cytokines after drug-eluting stent implantation in acute myocardial infarction patients with PCI

**DOI:** 10.1038/s41598-024-51496-8

**Published:** 2024-01-12

**Authors:** Minying Wan, Kun Hu, Yi Lu, Cheng Wang, Bin Mao, Qing Yang, Zhenzhong Zheng, Hao Wu, Yihong Luo, Amit K. Maiti

**Affiliations:** 1 Department of Cardiology, Chongming Branch, Shanghai Tenth People’s Hospital, The Affiliated Hospital of Tongji University, Shanghai, 202157 China; 2 Department of Cardiology, the First Affiliated Hospital of Nanchang University, Jiangxi, 330006 China; 3 Vascular Biology Program, Department of Surgery, Boston Children’s Hospital, Harvard Medical School, Boston, MA 02115 USA; 4 Department of Genetics and Genomics, 28475 Greenfield Rd, MydnavarSouthfield, MI 48076 USA

**Keywords:** Drug discovery, Cardiology

## Abstract

Acute Myocardial Infarction (AMI) after Percutaneous Coronary Intervention (PCI) often requires stent implantation leading to cardiovascular injury and cytokine release. Stent implantation induces cytokines production including TNFα, Hs-CRP, IL-1ß, IL2 receptor, IL6, IL8, and IL10, but their co-release is not extensively established. In 311 PCI patients with Drug-Eluting Stent (DES) implantation, we statistically evaluate the correlation of these cytokines release in various clinical conditions, stent numbers, and medications. We observed that TNFα is moderately correlated with IL-1ß (r^2^ = 0.59, *p* = 0.001) in diabetic PCI patients. Similarly, in NSTEMI (Non-ST Segment Elevation) patients, TNFα is strongly correlated with both IL-1ß (r^2^ = 0.97, *p* = 0.001) and IL8 (r^2^ = 0.82, *p* = 0.001). In CAD (Coronary Artery Disease)-diagnosed patients TNFα is highly correlated (r^2^ = 0.84, *p* = 0.0001) with IL8 release but not with IL-1ß. In patients with an increased number of stents, Hs-CRP is significantly coupled with IL8 > 5 pg/ml (t-statistic = 4.5, *p* < 0.0001). Inflammatory suppressor drugs are correlated as TNFα and IL8 are better suppressed by Metoprolol 23.75 (r^2^ = 0.58, *p* < 0.0001) than by Metoprolol 11.87 (r^2^ = 0.80, *p* = 0.5306). Increased TNFα and IL-1ß are better suppressed by the antiplatelet drug Brilinta (r^2^ = 0.30, *p* < 0.0001) but not with Clopidogrel (r^2^ = 0.87, *p* < 0.0001). ACI/ARB Valsartan 80 (r^2^ = 0.43, *p* = 0.0011) should be preferred over Benazepril 5.0 (r^2^ = 0.9291, *p* < 0.0001) or Olmesartan (r^2^ = 0.90, *p* = 0.0001). Thus, the co-release of IL-1ß, IL8 with TNFα, or only IL8 with TNFα could be a better predictor for the outcome of stent implantation in NSTEMI and CAD-diagnosed AMI patients respectively. Cytokine suppressive medications should be chosen carefully to inhibit further cardiovascular damage.

## Introduction

Vascular inflammatory responses include complex interactions involving ExtraCellular Matrices (ECMs) with various inflammatory cells, such as monocytes, macrophages, neutrophils, lymphocytes, Vascular Smooth Muscle Cells (VSMCs), and platelets. When ECMs undergo inflammatory activation in response to external stimuli, an increase in the expression of adhesion molecules such as selectins, Vascular Cell Adhesion Molecule-1 (VCAM-1), and InterCellular Adhesion Molecule-1 (ICAM-1) promotes adherence to the inflammatory cells. These monocytes, neutrophils, lymphocytes, and macrophages also recruit additional cytokines, growth factors, and Matrix Metalloproteinases (MMPs). When the injurious stimulus is removed, inflammation is generally terminated, and all the mediators disappear or are inhibited. The released cytokines include TNFα (Tumor Necrosis Factor α), chemokines, interleukins, interferons, colony-stimulating factors, and growth factors. If vascular inflammation progresses unresolved, it can lead to various vascular diseases^1^.

Percutaneous Coronary Intervention (PCI) is a landmark advance in the therapeutic history of Acute Myocardial Infarction (AMI) which reduces inpatient mortality and incidence of complications^2^. In PCI patients stent implantation induces over-inflammation leading to cytokine release^3^. Recent documentation about the release of various cytokines after stent implantation in various heart conditions has been reported. It is observed that STEMI-diagnosed diabetes patients have more inflammatory cells with severe atherosclerotic plaques than nondiabetes patients. Incretin therapy reduces inflammatory cells and MACE (Major Adverse Cardiac Events) in STEMI-diabetic Mv-NOCS (Multivessel Non-Obstructive Coronary Stenosis) patients with lower mortality^4^. Similarly, incretin users of NSTEMI-NOCs patients with diabetes showed a lower incidence of mortality and cardiac death compared to nondiabetic patients. The Incretin-based therapy exerts its effect by reducing inflammatory burden in diabetic NSTEMI-NOC patients^5^. PCI patients with diabetes also have an increased incidence of restenosis and stent thrombosis than non-diabetic patients. SGLT2 (Sodium/glucose cotransporter 2) inhibitor therapy reduced MACE in Type II diabetes (T2DM) and acute coronary syndrome patients. This reduction is attributed to the anti-inflammatory effect of SGLT2^6^. Due to higher oxidative stress hyperglycemic AMI patients have lower circulating EPC (Endothelial Progenitor Cells) and SIRT1 levels that differentiate EPCs^7,8^. Notably, it is demonstrated that SGLT2 therapy in PCI with hyperglycemic patients having similar stents induces an anti-inflammatory effect and increases SIRT1 level with EPCs that leads to myocardial regeneration and neovascularization leading to better clinical outcome^s^^9^.

The principle cytokines are mainly IL8 and TNFα with Hs-CRP (Hypersensitive C-Reactive Protein) for AMI^10,11^, TNFα, Hs-CRP, IL6 with STEMI patients ^12^, IL-1ß for coronary endothelial dysfunction in CAD (Coronary Artery Disease) patients^13^, IL6, IL8, and TNFα for patients with severe stenosis in a saphenous vein^14^, TNFα for restenosis patients after coronary angiography^15^, IL-1ß, IL6 and TNFα for AMI patients with significant stenosis of the ramus interventricularis anterior^16^. T2DM Mv-NOCS patients with SGLT2 inhibitor users showed reduced BMI, and inflammation than non-users with lower levels of NLRP3 inflammasome formation and IL-1ß^17^. Adiponectin that is exclusively secreted from adipose tissue and inflammatory cytokines including TNFα, PAI-1 (Plasminogen Activator Inhibitor type 1), IL-6, leptin, and resistin favorably modulate the endothelial inflammatory responses to vascular injury^18^ and linked to restenosis and Acute Coronary symptoms after PCI^19^. It is observed that lower levels of adiponectin and TNFα in preprocedural serum are significantly associated with the development of restenosis due to endothelium function impairment. Increased levels of resistin and adiponectin are significantly associated with better clinical outcomes in restenosis patients after PCI with angioplasty and DES^19^. Most importantly, a majority of these reports only studied a single cytokine release after stent implantation, without any correlation to several cytokines released together.

The outcome of stent implantation depends on several factors such as age, various clinical conditions, the number of stents, or the length/volume of stents being implanted. Six types of either plastic or metallic stent are generally used to maintain normal blood flow. Recently DES (Drug Eluting Stent) showed major advances in reducing morbidities and other health complications^20,21^. Since cytokine release is an inevitable outcome of stent implantation, a correlation of the extent of cytokine releases with the number of stent implantation or total stent length or volume may strengthen the hypothesis of cytokine-mediated complications.

Here we assessed the levels of TNFα, IL-1ß, IL2 receptor, IL6, IL8, IL10, and Hs-CRP and correlated their levels with various conditions in 311 AMI patients with PCI after metallic DES stent implantation. We studied the correlation of two or more cytokines release to understand whether they are occurring in a maximum number of patients significantly. Most of the studies earlier focused on single cytokine release after stent implantation, thus we enquired if the expression of two or more cytokines is correlated with the increased number or length, or volume of stent implantation. We observed that after stent implantation co-release of TNFα with IL-1ß or IL8 are the major cytokines that are correlated with various conditions, such as in diabetes and NSTEMI patients with AMI after PCI. These results could be confirmed with more recurrent studies with a larger cohort and would pave the way for better diagnostics and treatment options in PCI patients after stent implantation.

## Results

### TNFα is correlated with IL-1ß and IL8 in PCI patients after stent implantation

*STEMI (ST-Elevation Myocardial Infarction), NSTEMI (Non ST-Elevation Myocardial Infarction), CAD (Coronary Artery Disease), and UA (Unstable Angina).*

**Figure 1:**
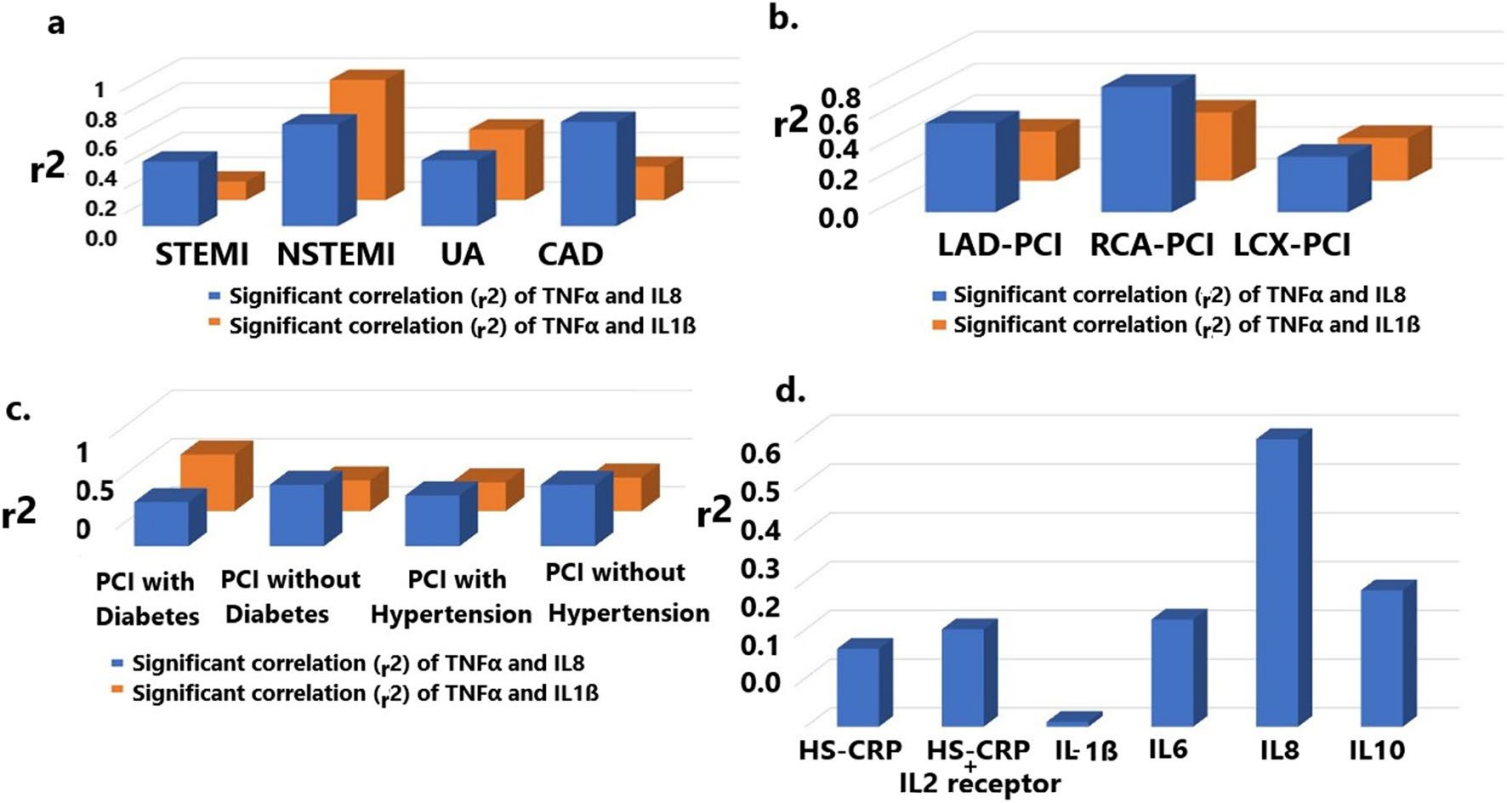
Correlation coefficient (r^2^) in various PCI patients with TNFα or Hs-CRP release. (**a**) r^2^ of TNFα and IL-1ß or IL8 in STEMI, NSTEMI, UA and CAD patients. (**b**) r^2^ of TNFα and IL8 or IL-1ß in patients with LAD-PCI, RCA-PCI and LCX-PCI. (**c**) r^2^ of TNFα release and IL8 or IL-1ß in various symptomatic conditions. (**d**) r^2^ of HS-CRP release and various cytokines in diabetes patients with PCI.

When PCI patients are diagnostically divided into STEMI, NSTEMI, CAD, and UA respectively, TNFα and IL8 levels are significantly correlated in NSTEMI (r2 = 0.82, p = 0.001) (Table 1, Fig. 1a) but moderately correlated in STEMI (r2 = 0.52, p = 0.014), and UA (r2 = 0.53, p = 0.001). In NSTEMI, TNFα and IL-1ß are also significantly and highly correlated (r2 = 0.97, p = 0.001) (Fig. 1a) but moderately with UA (r2 = 0.57, p = 0.001) and not with STEMI (r2 = 0.15, p = 0.6124). In CAD patients, TNFα is significantly correlated with IL8 secretion (r2 = 0.84, p = 0.0001) but not with IL-1ß secretion (r2 = 0.27, p = 0.101) (Table 1, Fig. 1a).

**Table 1:** TNFα level is correlated in various diagnosed PCI patients after stent implantation.

Diagnosis	Cytokines/Chemokines	Sample No	Correlation	r^2^ (Correlation coefficient) and p- value
NSTEMI	TNFα and IL8	20	High	r2 = 0.82, p = 0.001, 95% CI 0.5975–0.9275
STEMI	TNFα and IL8	23	Moderate	r2 = 0.52, p = 0.014, 95% CI 0.1412–0.7613
UA	TNFα and IL8	123	Moderate	r2 = 0.53, p = 0.001, 95% CI 0.3899–0.6464
NSTEMI	TNFα and IL-1ß	12	High	r2 = 0.97, p = 0.001, 95% CI 0.9018–0.9926
STEMI	TNFα and IL-1ß	13	Not Corelated	r2 = 0.15, p = 0.6124, 95% CI -0.4346–6496
UA	TNFα and IL-1ß	60	Moderate	r2 = 0.57, p = 0.001, 95% CI 0.3752–0.7229
CAD	TNFα and IL8	48	High	r2 = 0.84, p < 0.0001, 95% CI 0.7298–0.9074
CAD	TNFα and IL-1ß	36	Not significant	r2 = 0.27, p < 0.101, 95% CI 0.0554–0.8865)
LAD-PCI	TNFα and IL8	118	Moderate	r2 = 0.56, p < 0.0001, 95% CI 0.4232–0.6342
LAD-PCI	TNFα and IL-1ß	67	Poorly correlated	r2 = 0.31, p = 0.0086, 95% CI 0.08475–0.9015
RCA-PCI	TNFα and IL8	82	High	r2 = 0.79, p < 0.0001, 95% CI 0.7004–0.8640
RCA-PCI	TNFα and IL-1ß	49	Moderate	r2 = 0.43, p = 0.0021, 95% CI 0.1684–0.6389
LCX-PCI	TNFα and IL8	46	Moderate	r2 = 0.65, p < 0.0001, 95% CI 0.4432–0.7910
LCX-PCI	TNFα and IL-1ß	25	Moderate	r2 = 0.43, p = 0.032, 95% CI 0.04189–0.7052

#### Patients with LAD-PCI, RCA-PCI, and LCX-PCI

TNFα secretion also varies significantly with diagnosis in LAD-PCI (Mean = 44.7401, p < 0.0001) better than RCA-PCI (Mean = 42.77, p < 0.0001) as the mean patient number is more in LAD-PCI. TNFα is moderately correlated with IL8 in both LAD-PCI (r2 = 0.56, p < 0.0001) and, RCA-PCI (r2 = 0.79, p < 0.0001) (Fig. 1b, Table 1). However, TNFα is less correlated with IL-1ß in RCA-PCI (r2 = 0.43, p = 0.0021) and very poorly with LAD-PCI (r2 = 0.31, p = 0.0086). In LCX-PCI, TNFα and IL8 are moderately (r2 = 0.65, p < 0.0001) correlated but poorly (0.43, p = 0.032) with TNFα and IL-1ß.

### TNFα, IL-1ß, and IL8 release increases with the increased number of stents with various clinical conditions of PCI Patients

#### Age

We observed that PCI patient age is not correlated with diabetes (diabetes: r2 = -0.04, p = 0.39; no diabetes; r2 = 0.05, p = 0.39) or hypertension (hypertension: r2 = 0.12, p = 0.03; no hypertension; r2 =—0.12, p = 0.29). Similarly, age is neither correlated with TNFα secretion (r2 = -0.07, p = 0.39), IL8 secretion (r2 = -0.11, p = 0.15) nor with IL-1ß ((r2 = -0.11, p = 0.26)) in PCI patients.

#### Diabetes

In diabetes, TNFα and 1L-1ß are moderately correlated (r2 = 0.59, p = 0.001) (Table 2) but their association is less correlated (r2 = 0.32, p = 0.0103) with PCI patients without diabetic complications (Fig. 1c). PCI Diabetes patients exhibit less correlation with TNFα and IL8 secretion (r2 = 0.46, p = 0.0001) than PCI without diabetes (r2 = 0.63, p = 0.0001) (Table 2). For other cytokines, such as IL6 significantly but poorly correlated (r2 = 0.23, p = 0.045, 95% CI 0.0053–0.4413), but IL2 receptor (r2 = 0.088, p = 0.40, 95% CI -0.1195–0.2893) or IL10 (r2 = -0.04, p = 0.78, 95% CI -0.3249–0.2465) are not highly and significantly correlated.

**Table 2:** TNFα, IL-1ß, and IL8 release increases with an increased number of stents with various clinical conditions of PCI Patients.

Diagnosis	Cytokine/ Chemokine	Sample No	Correlation	r^2^ (Correlation coefficient) and *p*-value
PCI with Diabetes	TNFα and IL-1ß	49	Moderate	r2 = 0.59, p = 0.001, 95% CI 0.3741–0.7492
PCI without Diabetes	TNFα and IL-1ß	49	Poor	r2 = 0.32, p = 0.0103, 95% CI 0.0535–0.3766
PCI with Diabetes	TNFα and IL8	90	Moderate	r2 = 0.46, p = 0.0001, 95% CI 0.2848–0.6137
PCI without Diabetes	TNFα and IL8	218	Moderate	r2 = 0.63, p = 0.0001, 95% CI 0.5526—0.7111
PCI with Hypertension	TNFα and IL8	206	Moderate	r2 = 0.53, p = 0.001, 95% CI 0.4296–0.6258
PCI without hypertension	TNFα and IL8	102	Moderate	r2 = 0.64, p < 0.0001, 95% CI 0.5027–0.7427
PCI with Hypertension	TNFα and IL-1ß	125	Poor	r2 = 0.30, p = 0.005, 95% CI 0.1365–0.4560
PCI without hypertension	TNFα and IL-1ß	58	Poor	r2 = 0.35, p < 0.0068, 95% CI 0.1088–0.5592

#### Hypertension

TNFα and IL8 levels are significantly but moderately correlated (r2 = 0.64, p < 0.0001) (Table 2, Fig. 1c) with AMI patients without hypertension than AMI patients with hypertension (r2 = 0.53, p < 0.001). But TNFα and IL-1ß are neither correlated with patients with hypertension (r2 = 0.30, p = 0.005) nor with patients without hypertension (r2 = 0.35, p < 0.0068). We observed that the number of stents is normally distributed with hypertension. An increased number of patients with hypertension is associated with a higher number of stent implantation (Mean = 1.8103. p < 0.001, 95% CI 1.6669–1.9537) than the AMI group without hypertension (Mean = 1.6869, p < 0.001, CI 1.4881–1.8861) as the later has less Mean for stent Number. However, increased stent numbers are not correlated with TNFα either in AMI patients with (r2 = 0.20, p = 0.006, CI 95% 0.0570–0.3227) or without hypertension (r2 = 0.038, p = 0.70, 95% CI -0.1603–0.2340).

As expected, we observed that there is very little correlation between stent implantation directly with LDL_C (r2 = -0.22, p = 0.0064, 95#%CI -0.3723–0.0624) or HDL_C (r2 = 0.19, p = 0.0068, 95%CI -0.3435–0.0325).

### Stent implantation is associated with Hs-CRP release with IL8 in PCI patients

**Figure 2:**
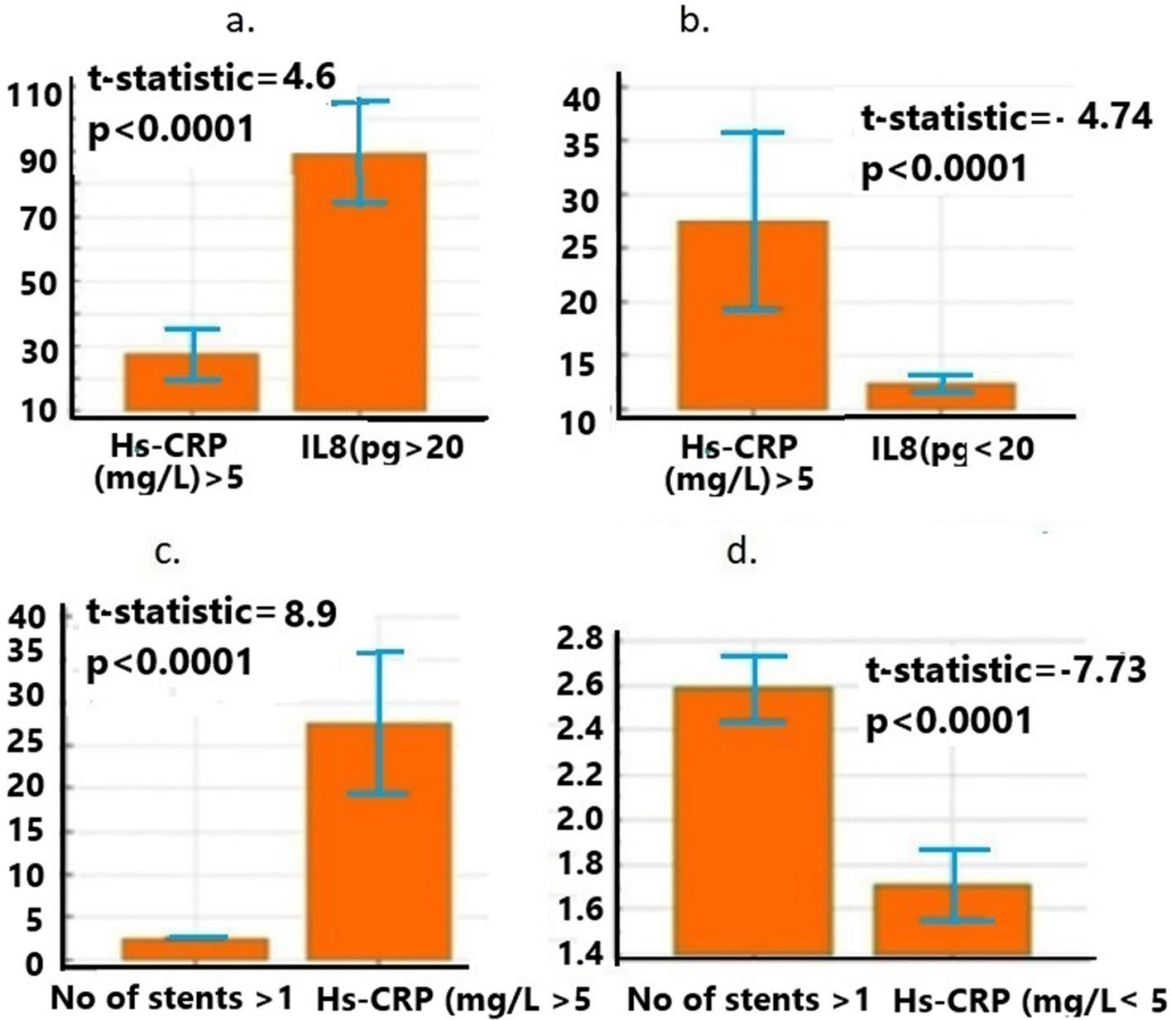
Student’s t-test statistic of Hs-CRP release and number of stents with TNFα and IL8 secretion. In t-test, error bar represents the 95% CI (**a**) Hs-CRP release at below < 5 mg/L, IL8 > 20 pg/L is significant (t-statistic = 4.6, p < 0.0001: 95% CI for Hs-CRP = 19.77–35.41 and for IL8 > 20 mg/L = 74.03–104.81) but (**b**) their co-release is not supported (negative t value) for IL8 < 20 pg/L (t-statistic = -4.74, p < 0.0001; 95% CI for HS-CRP = 19.41–35.41 and for IL8 < 20 mg/L = 11.75–13.31. (**c**) At the higher number of stent implantation, increased Hs-CRP level (> 5 mg/L) is also significantly released (t-statistic = 8.9, p < 0.0001, 95% CI for number of stent = 2.44–2.73 and for HS-CRP > 5 mg/L = 19.41–35.77) (**d**) but their co-release is not supported (negative t value) with below < 5 mg/L Hs-CRP level (t-statistic = -7.73, p < 0.0001, 95% CI for HS-CRP < 0.5 mg/L = 2.44–35.77 and for number of stent = 1.55–1.87).

We observed that the number of the stent (Mean) increases the level of Hs-CRP from < 5 mg/L (Mean = 1.7447, p < 0.0001, 95% CI 1.5952–1.8941) to above > 5 mg/L (Mean = 1.8730, p < 0.0001, 95% CI 1.6316–2.115) suggesting a higher number of stents is associated with higher Hs-CRP release. However, the Hs-CRP level does not correlate with age when the stent number is increased (r2 = 0.15, p = 0.1044) nor with blood glucose level (r2 = 0.16, p = 0.06) (Table 3). With increased stent number, an insignificantly poor correlation was observed in the Hs-CRP level with IL8 (r2 = 0.059, p = 0.5066) suggesting that their associated release does not occur as the stent number increases. Similarly, significantly but very little or almost no correlation was observed for IL6 release (r2 = 0.22, p = 0.02), for IL2 receptor (r2 = 0.20, p = 0.019), for IL10 (r2 = 0.28, p = 0.009) and IL-1ß (r2 = 0.01, p = 0.93) (Fig. 1d). As we did not observe any correlation with Hs-CRP and IL8 with an increased number of the stent, we sub-grouped IL8 level into two concentrations, > 20 pg/L and < 20 mg/L, and performed a t-test. The t-test shows that when Hs-CRP levels are > 5 mg/L and IL8 levels are > 20 pg/L (t = 4.5, p < 0.0001, 95% CI 35.2924–88.4235), their association is significant, but it is not associated with < 20 pg/L) (t statistic =—4.7, p < 0.0001, 95% CI -21.3233- -8.723) as t-statistic is negative (Fig. 2). An increased number of stents (> 1) is associated with Hs-CRP > 5 mg/L (t statistic = 8.9, p < 0.0001, CI 19.38–30.52) whereas no correlation is observed with increased stent number and a lower Hs-CRP level at < 5 mg/L (t statistic = -7.73, p < 0.0001, CI -1.09- -0.65).

**Table 3:** Stent implantation induces Hs-CRP release with IL8 in PCI patients.

Diagnosis	Stent	Cytokine/Chemokine	Sample No	Correlation	r^2^ (Correlation coefficient) and *p*-Value
Age	Increased	Hs-CRP	251	Poor	r2 = 0.15, p = 0.1044, 95% CI -0.02931–0.3088
Diabetes	Increased	Hs-CRP	73	Poor	r2 =—0.080, p = 0.5, 95% CI -0.3046—0.1527
PCI	Increased	Hs-CRP and IL8	128	Poor	r2 = 0.059, p = 0.51, 95% CI -0.1155—0.2304
PCI	Increased	Hs-CRP and IL-1ß	78	Poor	r2 = 0.01, p = 0.93, 95% CI -0.2139—0.2311
PCI	Increased	Hs-CRP and IL6	107	Poor	r2 = 0.22, p = 0.02, 95% CI 0.02714—0.3898
PCI	Increased	Hs-CRP and IL2 receptor	129	Poor	r2 = 0.20, p = 0.019, 95% CI 0.03458—0.3660
PCI	Increased	Hs-CRP and IL10	82	Poor	r2 = 0.28, p = 0.009, 95% CI 0.07419 -0.4741

### An increased number of stents is correlated with the release of TNFα and other major cytokines

**Figure 3:**
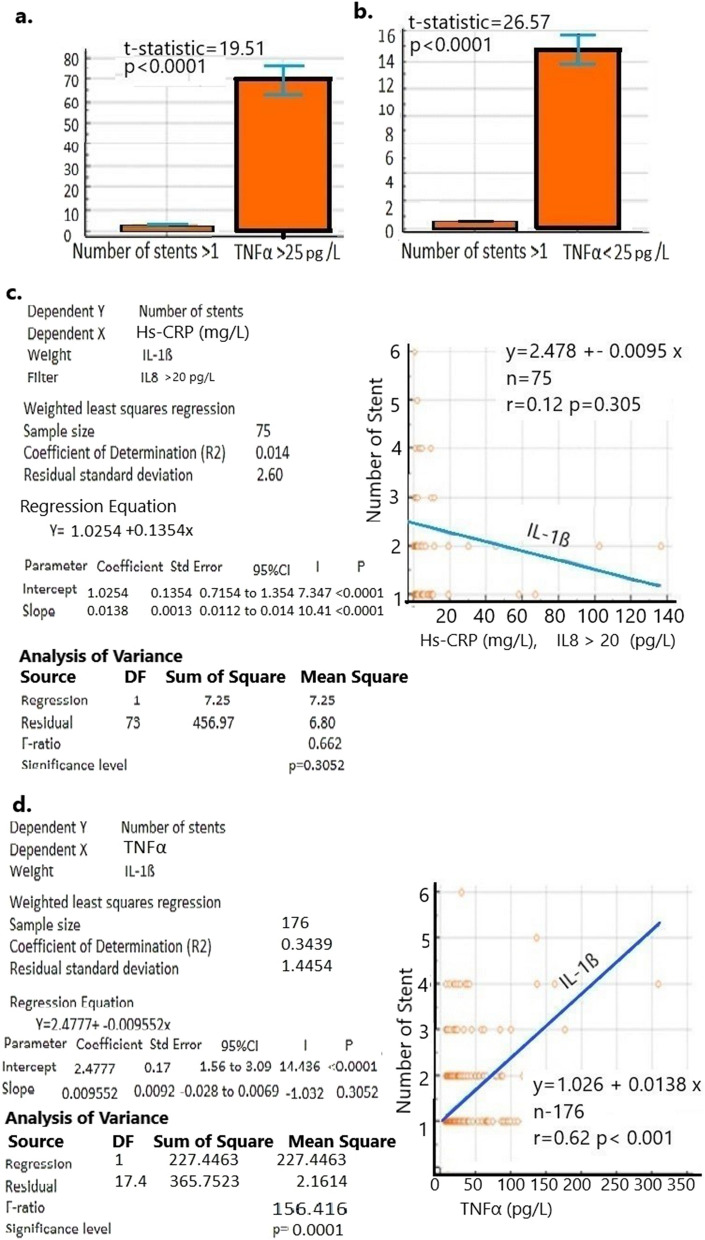
Increased number of stent implantation is significantly associated with TNFα, IL-1ß, and IL8. (**a**) t-test shows that the increased number of stent implantation is significant with TNFα release in both with positive t-statistic and p-value < 0.001 a) > 25 pg/L), error bar represents the 95% CI for stent number (2.44–2.73) and for TNFα (63.00–76.59) and (**b**) below < 25 pg/L of TNFα release. Error bar represents the 95% CI for stent number (2.44–2.73) and for TNFα (13.78–15.71). (**c**) Regression analysis shows that in patients with IL8 > 20 pg/L, number of stent and HS-CRP increase are not significantly associated with IL-1ß release (p = 0.3052) as IL-1ß is decreasing. In the left panel, the regression parameters and calculations are shown. (**d**) Regression analysis shows that the increased number of stent implantation with higher TNFα release is also associated with higher IL-1ß secretion. Orange rounds are patients, and the line represents the IL-1ß release increases as the TNFα and number of stents increases that starts from little more than 1(1.026 + 0.0138). Regression parameters and calculations are shown in the left panel.

We observed that an increased number of stents (Mean value) significantly varied with TNFα when the level is < 20 pg/L (Mean = 1.57, p < 0.0001, CI 95% 3.00–4.00)**.** Above this level at > 20 pg/L, the number of stents has no effect (Mean = 0.6, p = 0.039, 95% CI 0.5207–0.6793) as it implies that only 0.6 (0.5207–0.6793) number of stent implantation corresponds to > 20 pg/L TNFα whereas < 20 pg/L TNFα is associated with 1.57 (3.00–4.00 the number of the stent. Similarly, IL-1ß release also varies with an increased number of stents when < 5 pg/L (Mean = 1.69, p < 0.0001, 95% CI 3.00–4.000) than > 5 pg/L (Mean = 0.04, p < 0.0001, 95% CI 0.000–0.084). Other cytokines also vary significantly with an increase in the number of stents, such as IL2 receptor when it is both < 500 pg/L (Mean = 1.71, p < 0.0001, 97.5% CI 4.00–5.00) and > 500 pg/L (Mean = 1.86, p < 0.0001, 95% CI 4.00–5.00). An IL6 level > 3 pg/L also varies significantly with an increased number of stents (Mean = 1.86, p < 0.0001, 97.5 CI 4.00–5.000) than < 3 pg/L (Mean = 0.4, p = 0.06, 95% CI 0.3179–0.4917). The release IL8 is also dependent on the number of stents in both < 20 pg/L (Mean = 1.63, p < 0.0001, 95% CI 3.000–4.000) and > 20 pg/L (Mean = 1.86, p < 0.0001, 97.5% CI 4.000–5.00) whereas IL10 with < 5 pg/L (Mean = 1.7, p < 0.0001, 95% CI 3.00–4.00) than > 10 pg/L (Mean = 0.10, p =  < 0.0001, 95% CI 0.041–0.1630) are significantly dependent. When the number of the stent is greater than 1 and TNFα levels are either > 25 pg/L, (t statistic 19.515, p < 0.001, 95% CI 60.4148–74.007) or < 25 pg/L (t statistic 26.587, p < 0.001, 95% CI 11.26–13.06), they appear to be significantly associated (Fig. 3a, b). Similarly, TNFα and IL8 are significantly correlated with IL2 receptor at lower (< 500 pg/L) concentrations (r2 = 0.73, p = 0.0001, 95% CI 0.6644–0.7932) but moderately at higher concentrations (> 500 pg/L) (r2 = 0.46, p = 0.0001, 95% CI 0.2940–0.5954).

However, regression analysis shows that an increased number of stent implantation is slightly associated with TNFα and IL-1ß together (R^2^ = 0.38, p < 0.0001) when the IL8 level is > 20 pg/L. This association is not at all significant at < 20 pg/L (R^2^ = 0.30, p = 0.10). At the same level as IL8, TNFα is neither associated with the Hs-CRP level (R^2^ = 0.01440, p < 0.305) nor with any other cytokines like IL-1B (R2 = 0.07, p = 0.72), IL2 receptor (R2 = 0.008, p = 0.14), IL6 (R2 = 0.10, p = 0.12), and IL10 (R2 = 0.00, p = 0.998). With increasing stent number, Hs-CRP level is significantly associated when an IL8 level above 20 pg/L (R^2^ = 0.3712, p < 0.0001) (Fig. 3c) but not when IL8 is below 20 pg/L (R^2^ = 0.14, p = 0.3052). Again, with an increased number of stents, TNFα release is poorly associated with IL-1ß secretion (R^2^ = 0.38, p < 0.0001) (Fig. 3d).

### Left ventricular ejection fraction (LVEF) is negatively correlated with IL2 receptor

We studied whether ejection fraction (LVEF%) is correlated with any of these cytokine release with the increased number of stents (Supplementary Table 5). We did not observe any significant correlation except Hs-CRP and IL2 receptor. Hs-CRP is nearly significant but poorly co-related with a total length of the stent (r2 = 0.14, p = 0.055) but not (negatively) with a total number of the stent (r2 = -0.07, p = 0.5). EF is negatively and poorly but significantly correlated with IL2 receptor in both stent number (r2 = -0.27, p = 0.004) and total length of the stent (r2 = 0.23, p = 0.006) suggesting that the increase of LVEF% could be assessed with a larger cohort of patients to lower IL2 receptor release.

### Medications after PCI alters TNFα and IL8 or IL-1ß release

**Figure 4:**
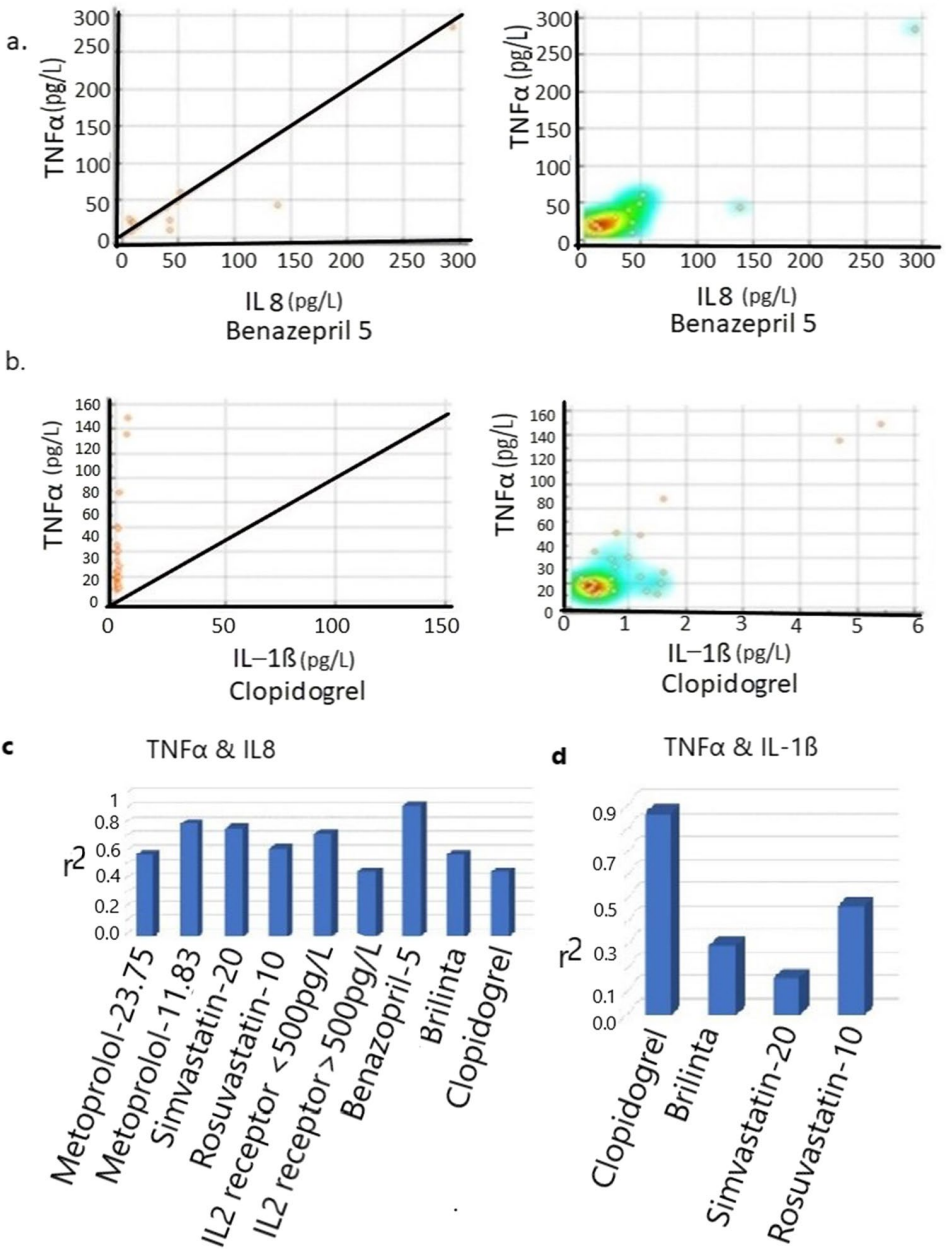
Selective ACE-ARB and antiplatelet drugs also suppress TNFα release in PCI Patients. (**a**) ACE-ARB drug Benazepril 5 is effective as it shows correlated with TNFα and IL8 release but no other ACE-ARB-drug. Left panel shows the distribution and right panel shows the heatmap of patients (maximum number of patients belong in the orange area) releasing these cytokines. (**b**) Similarly, antiplatelet Clopidogrel is correlated with TNFα and IL-1ß release but no other antiplatelet drug, such as Brilinta. Left graph shows the distribution and right graph shows the heatmap of patients with both TNFα and IL-1ß release. (**c**) TNFα is significantly correlated with IL8 in various medications used after stent implantation. (**d**) Significant correlation of TNFα with IL-1ß in various medications used after stent implantation.

During prognosis and diagnosis, stent number varies significantly better with simvastatin-20 (Mean = 1.648, p = 0.0042, 95% 1.000–4.000) than with Rosuvastatin-10 (Mean = 1.69, p = 0.0001, 95% CO 4.00–5.59). Both Simvastatin-20 (r2 = 0.77, p = 0.0001) and Rosuvastatin-10 (r2 = 0.63, p = 0.0001) (Table 4) are highly correlated with both release levels of TNFα and IL8. However, with Simvastatin-20, TNFα and IL-1ß are not significantly correlated (r2 = 0.16, p = 0.7014) but moderately associated with Rosuvastatin 10 (r2 = 0.47, p = 0.0001). Thus, Simvastatin-20 would be a better inflammatory suppressor for TNFα and IL8 release than Rosuvastatin-10. ACI/ARB treatment with Benazepril-5.0 (r2 = 0.93, p = 0.000) (Fig. 4a) or Olmesartan 20 (r2 = 0.90, p = 0.0001) is strongly associated with the release of TNFα and IL8 (Table 4) but not with other drugs, such as Valsartan 80 (r2 = 0.43, p = 0.0011).

**Table 4:** Medications are correlated with TNFα.

Medications	Cytokine	Cytokine	Sample No	Correlation	r^2^ (Correlation coefficient) and *p*-Value
Metoprolol-23.75	TNFα	IL8	204	Moderate	r2 = 0.58 p < 0.0001, 95%CI 0.4831–0.6660
Metoprolol 11.87	TNFα	IL8	6	Not significant	r2 = 0.80, p = 0.5306, 95%CI -0.0206–0.9666
Metoprolol-23.75	TNFα	IL-1ß	128	Moderate	r2 = 0.44 p < 0.0001, 95%CI 0.2872–0.5691
Metoprolol-11.87	TNFα	IL-1ß	3	Poor	r2 = 0.44 p < 0.0001, 95%CI 0.0–0.00
Simvastatin-20	TNFα	IL8	19	High	r2 = -0.55, p = 0.62, 95% CI 0.4842–0.9067
Rosuvastatin-10	TNFα	IL8	185	Moderate	r2 = 0.63, p = 0.0001, 95% CI 0.5325–0.7085
Simvastatin-20	TNFα	IL-1ß	8	Poor	r2 = 0.16, p = 0.7014, 95% CI -0.6124–0.7064
Rosuvastatin-10	TNFα	IL-1ß	113	Moderate	r2 = 0.47, p = 0.0001, 95% CI 0.3203–0.6079
Benazepril-5.0	TNFα	IL8	13	High	r2 = 0.93, p = 0.0001, 95% CI 0.7764–0.9791
Benazepril-5.0	TNFα	IL-1ß	5	Not significant	r2 = 0.51, p = 0.3735, 95% CI -0.6724–0.9608
Valsartan 80	TNFα	IL8	53	Moderate	r2 = 0.43, p = 0.011, 95% CI 0.1881–0.6320
Olmesartan 20	TNFα	IL8	13	High	r2 = 0.90, p = 0.0001, 95% CI 0.7114–0.9721
Brilinta	TNFα	IL8	259	Moderate	r2 = 0.58, p < 0.0001, 95%CI 0.4986–0.6598
Brilinta	TNFα	IL-1ß	154	Poor	r2 = 0.30, p < 0.0001 95%CI 0.1586–0.3344
Clopidogrel	TNFα	IL8	48	Moderate	r2 = 0.46, p = 0.0008, 95% CI 0.2128–0.6643
Clopidogrel	TNFα	IL-1ß	29	High	r2 = 0.87, p < 0.0001 95% CI 0.7425–0.9478

TNFα level also significantly but moderately varies with the antiplatelet drug Clopidogrel (Mean = 46.64, p < 0.0001, 95% CI 60.2343–270.4321) and Brilinta (Mean = 48.34, p < 0.0001, 95% CI 89.4843–122.4321). and TNFα is significantly but moderately correlated with IL8 with the antiplatelet drug Brilinta (r2 = 0.58, p < 0.0001) or Clopidogrel (r2 = 0.46, p = 0.0008). Similarly, TNFα is significantly and highly correlated with IL-1ß secretion for Clopidogrel (r2 = 0.87, p < 0.0001) (Fig. 4b, Table 4) but not for Brilinta (r2 = 0.30, p < 0.0001).

After stent implantation, when beta-adrenergic receptor Metoprolol-23.75 is used, TNFα levels are moderately correlated with IL8 (r2 = 0.58 p < 0.0001) (Table 4, Fig. 4c, d) but not significantly in lower dose with Metoprolol 11.87 (r2 = 0.80, p = 0.5306). Similarly, the TNFα level is significantly but moderately correlated with IL-1ß (r2 = 0.43, p < 0.0001) but not significant with Metoprolol 11.87 (r2 = -0.55, p = 0.62). Thus IL-1ß and IL8 are both moderately suppressed by Metoprolol 23.75 but not by Metoprolol 11.87.

### The extent of cytokine secretion does not differ with stent number or the total stent length

It could be hypothesized that “the total length of stent” may be more critical than the "number of stents" for the extent of cytokine secretion after stent implantation. The number of stents is highly correlated with the total stent length/volume as expected (Table 5). However, none of the criteria as Age, Diabetes or hypertension, and cytokine release is highly correlated with the total length of stent nor with the total number of stents (Table 5). In some of the cases like in coronary angioplasty results with RCA-PCI, LAD-PCI, and LCX-PCI, both numbers of the stent and the total length of the stent are significant but poorly correlated.

**Table 5:** Correlation of cytokine expression with stent number and total stent length.

Criteria	Sample No	No of Stent Correlation coefficient (r^2^)	*p*-Value	Sample No	The total length of the stent Correlation coefficient (r2)	*p*-value
No of Stent	––	–––		285	r2 = 0.69, 95% CI 0.6254–0.74	p = 0.001
Age	294	r2 = 0.09, 95% CI 0.-.029–0.19	p = 0.48	298	r2 = 0.04, 95% CI 0.-.073–0.15	p = 0.48
Hypertension	294	r2 = -0.057, 95% CI -0.056–0.17	p = 0.32	298	r2 = -0.18, 95% CI -0.13–0.95	p = 0.75
Diabetes	294	R2 = -0.14, 95% CI -0.033–0.25	p = 0.01	298	r2 = -0.04, 95% CI -0.066–0.67	p = 0.41
Blood glucose	299	r2 = -0.022, 95% CI -0.09–0.13	p = 0.69	297	r2 = -0.055, 95% CI -0.058–0.16	p = 0.34
HS-CRP	251	r2 = -0.030, 95% CI -0.15–0.09	p = 0.62	254	r2 = -0.029, 95% CI -0.15–0.09	p = 0.63
IL-1ß	176	r2 = -0.14, 95% CI -0.007–0.28	p = 0.06	177	R2 = -0.15, 95% CI -0.005–0.29	p = 0.04
IL8	292	r2 = -0.09, 95% CI -0.020–0.20	p = 0.1	296	r2 = -0.07, 95% CI -0.044–0.18	p = 0.22
IL_10	178	R2 = -0.012, 95% CI -0.13–0.15	p = 0.12	179	r2 = -0.03, 95% CI -0.18–0.10	p = 0.6
IL_2 receptor	294	r2 = -0.035, 95% CI -0.17–0.11	p = 0.95	298	r2 = -0.015, 95% CI -0.099–0.12	p = 0.79
IL6	294	r2 = -0.0035, 95% CI -0.11–0.11	p = 0.95	261	r2 = -0.05, 95% CI -0.17–0.11	p = 0.37
TNF	294	r2 = -0.14, 95% CI -0.031–0.25	p = 0.01	298	r2 = -0.021, 95% CI -0.091–0.13	p = 0.71
LAD-PCI	294	r2 = -0.18, 95% CI -0.29–0.07	p = 0.03	298	r2 = -0.10, 95% CI -0.21–0.013	p = 0.08
RCA-PCI	294	r2 = -0.16, 95% CI -0.048–0.27	p = 0.05	298	R2 = -0.14, 95% CI -0.033–0.25	p = 0.01
LCX-PCI	294	r2 = -0.17, 95% CI -0.28–0.06	p = 0.02	298	r2 = -0.16, 95% CI -0.048–0.27	p = 0.006
Metoprolol 23.75	259	r2 = -0.00	p = 1.0	261	r2 = -0.00	p = 1.0
Metoprolol 11.87	259	r2 = -0.00	p = 1.0	261	r2 = -0.00	p = 1.0
Valsartan 80	155	r2 = -0.00	p = 1.0	157	r2 = -0.00	p = 1.0
Benazepril 2.5	155	R2 = -0.090, 95% CI -0.24–0.06	p = 0.26	157	r2 = -0.064, 95% CI -0.21–0.93	p = 0.42
Rosuvastatin 10	286	r2 = -0.09, 95% CI -0.22–0.09	p = 0.10	289	r2 = -0.095, 95% CI -0.20–0.02	p = 0.10
Simvastatin 20	286	r2 = -0.0	p = 0.12	289	r2 = -0.0	p = 0.11
Olmesartan 20	155	r2 = -0.085, 95% CI -0.24–0.07	p = 0.29	157	r2 = -0.035, 95% CI -0.19–0.12	p = 0.6

## Discussion

The inflammatory biomarkers are potential non-invasive, diagnostic, predictive, prognostic, and therapeutic molecular biomarkers after implantations of stents^22,23^. Cytokine release is an obvious outcome after stent implantation. Although major cytokines like TNFα, Hs-CRP, IL-1ß, IL6, and IL8 are reported after stent implantation in several related disease conditions, such as AMI with PCI, stenosis, coronary angiography, coronary endothelial dysfunction, etc.^12–16^, their correlation of co-release in blood has not been extensively established with special emphasis on age, sex, gender, clinical conditions (diabetes, hypertension), various diagnosis criteria (e.g. STEMI, NSTEMI, UA, CAD) and for treatments with antiplatelet drugs, statins, and beta-blockers, etc.

TNFα is the most common cytokine release after stent implantation in PCI ^1,12,23^. Hs-CRP ^11,12,22^, IL-1ß^13,16^, IL6^14,16,24^, and IL8^10^ release have already been assessed independently. In diabetic patients after stent implantation, IL-1ß and IL8 release are significantly but moderately correlated with TNFα but less significant in nondiabetic patients. Although each of these cytokines’ release with TNFα has been observed previously in diabetic patients with PCI, their correlation of composed secretion was not established^10,13^. Our results suggest that in diabetic patients, IL-1ß and IL8 secretion with TNFα could be developed as targets for drug treatment after stent implantation to suppress inflammatory complications.

An increased number of stents is significantly correlated with TNFα and IL-1ß or IL8 release in PCI patients when diagnosed with NSTEMI but not with STEMI or UA. Kozel et al^12^ observed no difference in TNFα, IL6, or HS-CRP levels until after one year of metallic stent implantation in 46 STEMI patients which supports our results as our STEMI patients with DES did not show any significant correlation with any of these cytokine releases. In CAD we also observed the correlated expression of TNFα with IL8 but not with IL-1ß. No information or study was conducted earlier in NSTEMI, UA, or CAD patients for the release of these cytokines together. Nevertheless, a large cohort of patients can be recruited to validate the correlations of TNFα and IL-1ß or IL8 in NSTEMI or CAD patients. An increase in IL6 level is correlated with an increased number of stents, while the levels of IL-1ß, IL2 receptor, IL8, and IL10 are unchanged, indicating that these cytokines do not change with the increased number of stents in PCI patients.

Hs-CRP is a new independent index for CHD (Coronary Heart Disease) with a strong utility for forecasting cardiovascular disease. Hs-CRP level varies extensively after stent implantation in STEMI-diagnosed patients^12,22,23^. We observed an increased number of stent varies slightly with Hs-CRP levels at > 5 mg/L, but remains unchanged at basal levels (< 5 mg/L) but does not correlate with age or blood glucose level. With an increased number of stents, a poor correlation is observed between Hs-CRP and IL2 receptor, IL-1ß, IL6, or IL10 implying that their secretions are not coordinated. But a higher Hs-CRP (> 5 mg/L) level is significantly associated with both > 20 pg/L and < 20 pg/L concentrations of IL8. Although the release of these two cytokines is not studied earlier together, our observation suggests that IL8 release is coupled with Hs-CRP level and that could be potent diagnostics criteria after stent implantation in PCI patients.

The use of certain medications is obvious as a precaution to suppress inflammatory cardiovascular complications after stent implantation ^1,6^. In this study, all medications related to the suppression of inflammation were used within 2–3 h after stent implantation. The suppressive effect of medication on inflammatory cytokine would represent a negative or poor correlation. To develop a prognostic approach after stent complications, it is extremely important to correlate their secretion in blood in a cumulative way for understanding their full function.

Beta-blockers are shown to effectively suppress inflammatory molecules, such as TNFα and IL10 in cardiomyopathy patients^25^. During AMI, the leukocytes especially neutrophils and monocytes readily migrate to the injury site to clear the dead cells and debris as delayed response leads to maladaptive tissue remodeling or scar formation with a negative impact on heart function. VCAM1 and CCL2 positively and CCR2 negatively regulate the migration of these leukocytes. Chronic beta-blocker treatment increases VCAM1 and decreases CCR2 expression to augment the recruitment of leukocytes and reduce the severity of innate immune responses leading to increased wound healing capacity at the injury site^26^. In addition, ß1-AR (beta-adrenergic receptor) antagonist Metoprolol treatment also increases VCAM1 expression and leukocyte accumulation while reducing CCR2 expression and alters the leukocyte function for responsiveness to acute injury. We observed that a lower dose of beta-blocker Metoprolol-11.87 is highly correlated with the increased level of TNFα and IL8 but moderately with a higher dose of Metoprolol-23.25. Thus, the effect of a higher dose of this beta blocker could have a better suppressive effect after stent implantation which could be further evaluated with other supporting studies.

Rosuvastatin-10 users have higher TNFα and IL8 or IL-1ß levels than the patients using Simvastatin-20 or Simvastatin-10. This implies that having similar blood vessel damage Simvastatin better suppresses cytokine releases than Rosuvastatin-10. Thus, after stent implantation before choosing any of these drugs, it could be helpful to determine the correlated release of TNFα and IL-1ß or IL8 levels.

Antiplatelet drug Clopidogrel, but not Brilinta, is more effective for suppressing the effect of TNFα and IL8. TNFα varies significantly with the antiplatelet drug Clopidogrel and it conjugately varies with IL8 when both Clopidogrel and Brilinta are used. Several combinations, such as Dual Antiplatelet Therapy (DAPT) are helpful to reduce morbidities but increase DAPT-related bleeding complications and stent thrombosis ^27^,^28^. After discontinuing DAPT Bioabsorbable polymer Everolimus-Eluting Synergy Stents in high-risk patients reduces these symptoms^29^.

TNFα and IL8 levels are significantly correlated with ACI/ARB drug Benazepril 5.0 or Olmesartan 20 but not with Valsartan 80 or Perindopril 4. However, further research and supporting evidence are needed to evaluate whether Perindopril 4 or Valsartan 80 should be preferable to Benazepril 5.0 or Olmesartan 20 after PCI intervention with stent implantation. It also appears that the differences in the number/length/volume of the stent may not have a profound effect on cytokine release or other factors such as Age or hypertension or medications used.

Several approaches could be manifested to neutralize these cytokines to reduce inflammation after stent implantation. TNFα antibody eluting stents are shown to reduce restenosis in saphenous vein organ culture in vivo and may have potential clinical benefit in PCI ^30^. Further, these TNFα antibodies eluting stents may be conjugated with IL-1ß or IL8 based on their correlation with stent implantation in PCI patients. Apart from this strategy, intravenous anti-TNFα (etanercept, Adalimumab, Infliximab)^31,32^, anti-IL8^33,34^ or anti-IL-1ß (canakinumab, gevokizumab)^35–37^ gene disruption or treatment are shown to reduce myocardial infarction (MI) in animal models, human patients or human cells^38–41^. However, the Clinical trial with anti-TNFα alone did not reduce heart failure possibilities in heart failure patients^42,43^ and has been attributed to the counterplay of TNF receptor, TNFR1, and TNFR2 mediated inflammasome activation^35^. Nevertheless, IL-1ß or IL8 treatment with TNFα could have the potential clinical benefit after stent implantation. The advantages of using these antibodies are already used for various heart ailments, and their safety issues are well documented.

## Limitations

This is a single-center study that includes evaluations of cytokine release after stent implantation. Although we considered the total length or volume of stents by the addition of several small stents, a longer single stent could affect cytokine secretion differently which has not been addressed. Stent length/volume may be extensively assessed in future studies with more subtle criteria to evaluate the extent of cytokine secretion. ACS (Acute Coronary Syndrome) is an acute occlusion of a coronary artery segment or an at least relevant stenosis leading to schema and hypoxia of the myocardium, respectively. So, the measured cytokines here could also represent the cumulative effect of both processes rather than from the damage of the vascular wall alone after stent implantation. Whatever the reason for cytokine secretion, we raised the possibility that instead of studying single cytokine, correlating two or multiple cytokine releases may be better predictors of various conditions in PCI patients after stent implantations.

## Conclusions

Stent implantation causes cardiovascular injury that leads to multiple cytokine releases but their co-release has not been extensively established. We showed here that in diabetic PCI patients, IL8 and IL-1ß release is correlated with TNFα release, thus together they could be a predictor of complications and should be suppressed. Similarly, in NSTEMI patients, TNFα, IL-1ß, or IL8 release is significantly correlated with an increased number of stents and thus needs further attention to treat these patients. In CAD patients, TNFα secretion is correlated with IL8 but not with IL-1ß. Our data also suggest that the release of TNFα and IL8 is better suppressed by beta-adrenergic receptor Metoprolol 23.75 but not with its lower dose and also better suppressed by Clopidogrel than Brilinta. TNFα and IL-1ß release is poorly correlated with Simvastatin-20 but not with Rosuvastatin-10, thus former should be evaluated for better clinical outcomes in further studies. We also observed that after stent implantation, Valsartan 80, or perindopril 4, should have higher efficacy than Benazepril 5.0 or Olmesartan 20. Our results suggest that after DES implantation, measurements of TNFα, IL8, and IL-1ß and their correlation analyses could be evaluated further in a large cohort to select drugs to reduce over-inflammation-mediated cardiovascular complications to prevent morbidities.

## Materials and methods

### A Ethical statement

The Ethics Review Committee of the Chongming Branch of Shanghai Tenth Peoples Hospital approved this study (Ethics Number: EC20221104-1001, Dated: 2013–09-10). **All methods were performed following the relevant guidelines and regulations**. All subjects provided written informed consent before participating in this study. During CAD evaluation both the angiography and coronary stent implantation were completed voluntarily and were selected by the patients and their family members. The patient’s family members signed the informed consent forms in all cases.

### Recruitment of patients

Inpatients were admitted to the Department of Cardiology, Xinhua Hospital, Shanghai Jiaotong University School of Medicine from 2014 to 2015. All blood biochemistry, electrocardiogram, echocardiography, etc. were examined in the hospital. All patient records and test results were collected after discharge. The patient diagnosis, measurements, and treatment outcomes are in Supplementary Tables 1, 2, and 3.

### Inclusion and exclusion of patients

During the study period (01/2014–05/2015, 17 months), approximately ~ 40 patients were recruited every month (a total of 672 patients). Most patients who refused to have a stent implanted (n = 32) or required surgical bypass (n = 221) or could not be followed up (n = 31), so they were excluded from this study. All patients with STEMI, NSTEMI, UA, and CAD are diagnosed based on international guidelines. Some patients did not give consent for a research study (n = 14). Other patients (n = 63) have different types of stents except for DES of various shapes and unique manufacturers that are excluded from the study analysis. 311 patients are included in the study with similar types of stents (mostly balloons).

### Detailed procedures

#### STEMI (ST-Elevation Myocardial Infarction) patients:

STEMI patients have a significant elevation of ST segment in ECG associated with myocardial ischemia with persistent chest discomfort or other associated symptoms suggestive of ischemia as shortness of breath, nausea, fatigue, and palpitations. STEMI was diagnosed based on international guidelines as at least two anatomically contiguous leads (for men < 40 years, > 2.5 mm; < 40 years, > 2 mm; for women, > 1.5 mm, for men and women, V4R, V3R, V7-V9 > 0.5 mm). Elevated cardiac troponin values were at least one value above the 99^th^ percentile.

#### NSTEMI (Non-ST Segment Elevation (NSTEMI) Myocardial Infarction) patients

NSTEMI patients have elevated cardiac troponin levels but no ST-segment elevation in ECG. According to the Grace score, very high-risk patients received coronary angiography within 2 h with PCI; moderate-risk patients received coronary angiography within 24 h with PCI; low-risk patients received coronary angiography within 72 h with PCI treatment.

#### UA (Unstable Angina)

UA has the same timing as NSTEMI that are collectively referred to as NST-ACS (non-ST-segment elevation-Acute Coronary Syndrome) those have neither ST-segment elevation nor passed the elevated troponin level criteria. According to the Grace score, very high-risk patients underwent coronary angiography within 2 h after PCI; intermediate-risk patients underwent coronary angiography within 24 h after PCI; and low-risk patients underwent coronary angiography within 72 h after PCI.

#### CAD (Coronary Artery Disease)

For CAD patients having symptoms of chest pain and discomfort, non-invasive examinations, such as electrocardiogram, exercise treadmill test, etc., are generally performed first. If myocardial ischemia is observed, coronary angiography is performed. If the vascular stenosis is > 75%, PCI treatment is performed.

##### PCI

After an initial assessment, primary PCI was performed within 3 h of hospital admission as a standard procedure by inserting a catheter into the artery that released a "radio-opaque dye (iodine-based) to locate the lesions clearly by a real-time x-ray imaging. The catheter left the DES spanning the lesions in the artery.

For STEMI patients, when the STEMI onset was less than 12 h, they underwent emergency PCI; When the onset was more than 12 h, they first received conservative treatment with drugs; If the patient's condition was relieved and the vital signs were stable, then PCI was performed after one week; if the patients were not well remitted or if there was progressive aggravation, they were given emergency PCI.

#### LAD-PCI, RCA-PCI and LCX-PCI

Percutaneous Coronary Intervention (PCI) of lesions in the proximal Left Anterior Descending coronary artery (LAD) is worse in conditions than proximal Right Coronary Artery (RCA) and Left Circumflex coronary artery (LCX).

#### Stent implantation

The severity of the patient's disease and the damage to the blood vessels combined with the consent of their families were considered for selecting stents, which vary based on length, number, and manufacturer. In some patients, a single blood vessel has a long course. In some patients with multivessel disease, multiple stents of the same type were implanted. All stents are drug-eluting metal stents (DES) that are imported (Lepu, Medtronic, Resolute) or domestically obtained (JWMS). Most of them used in this study are balloon stents of Firebird, Excel, NANO, and TIVOLI. We limited our study analysis to the level of cytokine and chemokines from similar types of stents to minimize the errors. As diverse types of stents of various lengths, types, or volumes may affect the release of different levels of cytokines and complicate the analysis,

#### Medications

Patients admitted to the hospital with a diagnosis of ACS (including STEMI, NSTEMI, UA, and CAD) are treated with antibodies, anticoagulants, enhanced statins, beta receptor antagonists, ACEI, or ARB. Medications are started approximately within 2–3 h after stent implantation. The drugs used were Brilinta (AstraZeneca Pharmaceuticals, China), Metoprolol (AstraZeneca Pharmaceuticals, China), Clopidogrel (Shenzhen Xinitai Pharmaceuticals, China), Simvastatin (Hangzhou Merck), Rosuvastatin (AstraZeneca Pharmaceuticals, China), Valsartan, Olmesartan, Benazepril (Changzhou Siyao Pharmaceuticals, China).

#### Cytokines (IL-6/IL-8/IL-10/TNF-a/IL-1B/Hs-CRP) and cholesterol measurement

For emergency PCI patients after stent implantation, blood was drawn after 12 h. Cytokines (cytokine kit, LS BIO, WA, USA) and Hs-CRP (Hs-CRP kit, Abnova, USA ) were measured immediately by a two-site chemiluminescent enzyme immunometric assay in an IMMULITE analyzer (SIEMENS Healthineers, Germany). Cholesterols were measured (HDL and LDL assay kit, LS BIO, WA, USA) using standard laboratory equipment, Hitachi 7104 Analyzer (Hitachi, Tokyo, Japan).

#### SYNTAX score

The SYNTAX score, an angiographic score, was applied to assess the severity and complexity of cardiovascular disease and the severity of coronary lesions ^44^. The total SYNTAX score for each participant was calculated by taking the sum of the total points assigned to each lesion with > 50% stenosis and > 1.5 mm diameter in coronary arteries. Two observers independently calculated the SYNTAX score based on Coronary Angiography. The participants were divided into three subsets according to SYNTAX score: Group 1: score ≤ 22, Group 2: score = 23–32, Group 3: score > 33 ^45^.

#### Total length/volume of stent

The total length/volume of the stent was calculated by multiplying the dimension of each stent (cubic mm). This value is again multiplied by the number of stents for a single type or multiple types of cubic mm value of each stent are added as specified for each patient.

### Statistical analysis

Statistical analysis was done using various software such as SPSS 16.0 and MEDCALC etc. To calculate the simple arithmetic means with the significance of occurrence for the normalized distribution, a D’Augustino Pearson Test or Chi-square test was done.

For all statistical analysis, a single data table was prepared to consist of all parameters provided in supplementary Tables 1, 2, and 3 and used as data in the MEDCALC software.

### Summary statistics

For determining the arithmetic mean and normal distribution, parameters were selected manually with various combinations, such as age vs number of stents, number of stents vs IL8 level, and so on. In each case, D'Augustino Pearson Test is selected for normal distribution, and software output of arithmetic mean, normal distribution, 95% Confidence Intervals (CI), and acceptability of normal distribution are mentioned.

### Correlation

R^2^ (correlation coefficient) was calculated by using the correlation coefficient test using the same data table. In each case, variable X and variable Y are selected, such as X = TNFα and Y = IL8 with a filter, such as Hypertension = "Yes". Similarly, to assess the effect of stent implantation, the number of stents > 1 is set for filter and other covariates such as EF% in Y-axis, and other cytokines, such as IL8, IL-1B, etc. in the X-axis. For results, r^2^, p-value, and 95% CI are noted from the software output. Depending upon the r^2^ value as > 0.80-high, 0.4–0.8-moderate and 0.0–0.4-poor were designated as the extent of correlation with a p-value below < 0.05 as significant.

### Regression analysis

Regression (designated here as R^2^) and logistic regression were done by calculating intercept and slope by plotting x and y with the use of each criterion in the data. Both independent and paired t-tests were used to test the hypothesis of the release of cytokines together.

Baseline characteristics of PCI patients for statistical analysis with various criteria are listed in Supplementary Table 4 (Baseline characteristics). A Kolmogorov–Smirnov test was used to assess the normality of the distribution for all variables. The distribution of biochemical markers among the participants, excluding the blood glucose, TG, TC, LDL, and HDL values, was skewed in this study. The skewed variables were expressed in the median, and the interquartile range was analyzed after a logarithmic transformation. The correlation between the inflammatory cytokines was analyzed with a Spearman coefficient. We compared the inflammatory cytokines of participants using one-way ANOVA. Generalized linear regression analysis was performed to determine the association between inflammatory cytokines and various conditions. All tests were two-sided and p < 0.05 was considered statistically significant.

## Informed consent

All participants consented to the study.

### Supplementary Information

Supplementary Information.

## Data Availability

All data will be available to the researcher upon request to Dr. Minying Wan, Dr. Yihong Luo, or Dr. Amit K Maiti.
