# Supplementary material for: Co-release of cytokines after drug-eluting stent implantation in acute myocardial infarction patients with PCI
Source: Sci Rep. 2024 Jan 12;14:1236. doi: 10.1038/s41598-024-51496-8 (PMC10786845; doi:10.1038/s41598-024-51496-8)
Supplement: Supplementary file 1 — Supplementary Information. [file 41598_2024_51496_MOESM1_ESM.zip › pci-suppl table 2.pdf]

Suppletable 2: Patient's leision, procedural and stent information

Name	ECG	Left front drop	(Detour)	No obvious stenosis	No of stents	Stents	Stent volume/length-1(mm^3)	Stent volume/length-2(mm^3)	Stent volume/length-3(mm^3)	Stent volume/length-4(mm^3)	Total STENT Length/volume (mm^3)
a1	Acute anterior myocardial infarction	80% stenosis in the middle	85% stenosis in the proximal of OM1	70% of the most stenosis	1	2.75 * 18mm EXCEL balloon stent	49.5				49.5
a2	Normal ECG	50% stenosis of the distal part of the stent	More than 90% stenosis in the middle segment	50% stenosis in the proximal segment, 90% stenosis in the middle segment	3	4.0*15?2.5*30mm baloon RESOLUTE	60	75			135
a3	Normal ECG	70-80% stenosis in the near middle segment and 50% stenosis in the far segment	60% of the most stenosis	60% stenosis in the middle segment, 40% stenosis in the distal segment	2	3.5 * 18mm EXCEL balloon stent	126				126
a4	Sinus bradycardia, ST-T changes	Diffuse lesions, the most stenosis in the distal segment 70%	The original LCX stent is unobstructed, 40% stenosis in the middle of the OM, and 100% occlusive in the distal segment	Diffuse calcification, the distal stent in the proximal segment patency, 50% stenosis in the middle segment, 99% stenosis in the opening of PD	2						

a5	Sinus bradycardia, III, aVF showed QS, ST: II, III, aVF increased 0.5-1mm, ST: I, aVL decreased 0.5-1mm, T: V3-V4 biphasic, V5-V6 inverted	90% stenosis of the opening, middle myocardial bridge, 90% stenosis during contraction	Irregular vessel wall in the proximal mid-segment	Distortion in the proximal segment is obvious, TIMI0-1 level							
a6	Sinus bradycardia	50% stenosis in the proximal segment, 95% or more in the middle segment	Advantages, 40-50 % stenosis in the distal segment	Small and 50% stenosis in the proximal segment	1	2.75 * 24 sheet baloon stent	60				60
a7	Normal ECG	65% stenosis in proximal segment	90% stenosis in the middle distal segment	The proximal vessel wall is irregular, 80% stenosis in the middle segment, and the posterior descending branch is completely occluded	3	Resolute baloon 2.25*8mm,3.5*36mm baloon EXCEL?3.5*14mm baloon EXCEL	18	126	49		193
a8	II, III, aVF is qR	40% stenosis at the opening and proximal segment	50% stenosis in the proximal mid-segment	99% of the most stenosis	1	3.0 * 36mm EXCEL balloon stent	108				108

a9	Inferior and posterior wall myocardial infarction may be possible, ST: I, aVL level is reduced by 0.5mm	Diffuse lesions in the proximal middle segment, the most severe stenosis is 85%, and the distal stenosis is 40%	80% of the most severe stenosis in the proximal mid-segment	70% stenosis in the proximal segment, middle and distal segments are completely blocked	1						
a10	IRBBB, ST: V2-V6 horizontal depression 1-4mm	Proximal mid-segment lesions, 80% stenosis	More than 90% stenosis from the middle segment of the lesion	Long lesions in the proximal middle segments, 90% stenosis, long lesions from the distal segment to PD	2	2.5 * 26, 2.75 * 18 NANO balloon stent	65	49.5			114.5
a11	Sinus palsy, III, aVF is qR	Long lesions near the middle segment, 90% stenosis, 90% stenosis in the mid-distal segment	80% stenosis in the middle distal segment	40-50% stenosis in the proximal middle segment, and 100% occlusion in the middle and distal thrombosis	2	3.0 * 23mm Firebird Balloon Stent	138				138
a12	Ventricular premature beats	Irregular vessel wall	Irregular tube wall in the proximal segment, almost 100% occlusion in the distal segment	Irregular vessel wall	1	2.5 * 33mm Firebird Balloon Stent	82.5				82.5

a13	ST-T changes, prolonged QT	The longest part of the diffuse long lesion is the narrowest 95%, shows the lateral branch supplying the distal RCA	Irregular tube wall with 50% stenosis in the proximal segment	Diffuse lesions in the proximal segment, 99% of the most stenosis in the middle segment	4	3.5*35mm tivoli 3.5*12,mm baloon stent 3.0*29?2.75*29 mm FIREBIRD baloon	122.5	42	87	79.75	331.25
a14	ST: V1-V4 is raised by 2-4mm, ST: I, V6 level is lowered by 0.5-1mm, T: I, II, aVR, aVL, V4-V6 is inverted	99% of the proximal stenosis with plaque is not hidden, and 70% of the proximal stenosis	No obvious stenosis	No obvious stenosis	1	TIVOLI 3.5*21mm baloon	73.5				73.5
a15	Left ventricular high voltage, ST-T changes	Diffuse lesion, 85% stenosis in the proximal segment	99% narrow in the middle segment	The proximal segment is completely occluded, and the distal segment is supplied with left coronary collaterals	1	3.5*15mm LEPU baloon stent	52.5				52.5
a16	Normal ECG	Complete occlusion from the proximal segment, D1 and proximal stenosis 80%	The closest severe stenosis is 80%, with unstable plaque	Diffuse long lesions in the whole process, the most severe stenosis in the proximal middle segment is 75%, the most severe stenosis in the middle segment is 85%, and the distal segment is completely occluded							

a17	Left ventricular high voltage	Diffuse calcification, 80% narrowest in the proximal segment, 70% narrow in D1 opening	Diffuse lesion with 50% of the most stenosis	95% stenosis in the middle of the original stent with thrombosis, 90% stenosis outside the distal stent	2	4.0*12mm NANO balloon stent	57.6				57.6
a18	Lower Myocardial Infarction, ICRBBB	70-80% stenosis in the proximal segment	100% stenosis in the distal segment	The original stent is unobstructed, and the distal end is completely occluded	2	2.75*24mm resolute baloon stent	66				66
a19	Sinus rhythm, grade I AVB, possible acute myocardial infarction	90% stenosis in the proximal segment	Middle segment occlusion	The middle segment is occlusion	1	3.5*36mmEXCEL balloon stent	126				126
a20	ST: V5-V6 horizontal depression 0.5mm	Irregular vessel wall	The wall of the proximal and middle segments is irregular, 40-50% stenosis, and the middle and distal segments are 90% stenosis	Irregular vessel wall	1	3.0*24mmEXCEL balloon stent	72				72
a21	Normal ECG	99% stenosis of proximal stent	No obvious stenosis	No obvious stenosis	2	3.5*12mm NANO baloon	84				84
a22	CLBBB, T wave changes	40% narrower opening, the original stent is unobstructed	40% stenosis in the opening and 90% stenosis in the distal segment	100% occlusion in the middle segment	1	3.5*12mm Firebird baloon	42				42

a23	Normal ECG	99% narrow in the proximal segment	No obvious stenosis	No obvious stenosis	1	3.5*18mm Firebird balloon stent	63				63
a24	ST: The back of the II, III and aVF arches are raised 1mm upwards, AVB of I degree	85% narrow in the proximal segment	No obvious stenosis	60% stenosis in the proximal segment, 95% stenosis in the middle thrombosis, and 70% stenosis in the PD opening	1	3.0*18mmEXCEL balloon stent	54				54
a25	II, III, aVF is qR, T: III, aVF is reverse	60% narrow in the proximal segment	Irregular vessel wall	Almost 100% occlusion in the anterior trigeminal segment	1	3.0*14mmEXCEL balloon stent	42				42
a26	T: T, aVL, V5 dual phase, V4 inverted	99% narrow in the proximal mid segement	Irregular vessel wall	Irregular vessel tube wall throughout the segments, 40% stenosis in the proximal middle segments and 50% stenosis in the distal segment	1	3.5*28mmEXCEL balloon stent	98				98

a27	Acute posterior wall myocardial infarction, T: II, III, aVF flat, V4 biphasic, V5-V6 inverted, ST: V1-V4 horizontal depression 0.5-1.5mm, Q-T extended	Diffuse long lesions near the middle, the most severe stenosis is 90%, the D1 opening stenosis is 90%	100% occlusion in the proximal middle segment	Long lesions from the proximal to the distal segment, with about 70% stenosis%??	2	2.5*24mm?3.0*14mmEXCEL balloon stent	60	42			102
a28	ST: V1-3 raised 1-2mm	75% stenosis in the proximal segment, 80% stenosis in the proximal segment	40% opening, 95% stenosis in the middle segment	No obvious abnormalities	2	2.75*18mm?2.5*18mm balloon stent	49.5	45			94.5
a29	ST: The back of the II, III, aVF arch is raised 0.5-1mm, ST: I, aVL, V3-V6 is depressed 0.5-2.5mm, III, aVF is qR, CRBBB, T: II, III, aVF is inverted	Opening stenosis is 50%, proximal lesions with calcification, the most severe stenosis is 75%, the middle segment stenosis is 70%, the middle and distal segments	75% stenosis in the opening, OM1 is completely occluded	30% stenosis in the opening, 90% stenosis in the proximal segment with calcification, and complete occlusion in the middle segment	2	TIVOLI 2.5*18mm,3.5*15mm balloon stent	45	52.5			97.5

a30	V1 is QR	90% stenosis in the proximal middle	80% stenosis in the proximal middle segment and 70% stenosis in the distal segment	50% stenosis in the proximal segment, 99% stenosis in the middle segment, and 90% stenosis in the distal segment	4	2.5*18mm,2.75*24 baloon, 2.75*18mm, 3.0*33mmEXCEL balloon stent	45	66	49.5	99	259.5
a31	First degree AVB, IRBBB	Middle myocardial bridge, 70% stenosis during contraction	Irregular vessel wall	99% stenosis in the proximal segment	1	2.5*30mm resolute baloon stent	75				75
a32	Normal ECG	85% stenosis in the proximal middle segement	No obvious stenosis	No obvious stenosis	1	4.0*18mmEXCEL balloon stent	72				72
a33	Lower Myocardial Infarction, CRBBB	50% narrow in the middle	More than 90% stenosis in the middle and distal segment	95% stenosis in the proximal segment	1	3.0*21mmLEPU baloon stent	63				63
a34	Normal ECG	50% proximal stenosis	80% stenosis is in the middle segment, middle distal stent is unobstructed	Irregular vessel wall, 30% stenosis in the middle segment	2	3.0*28mmEXCEL balloon stent	84				84
a35	I degree AVB	The proximal section of the stent is unobstructed, and the distal section is about 80%	99% stenosis in the proximal segment, the original stent in the middle segment is unobstructed	Nearly 60% stenosis, mild intimal hyperplasia in mid-stent	3	3.0*12mm stent	108				108

a36	T: I, aVL low level	Lesions throughout, 50-60% narrowest	More than 80% of the most stenosis in the middle segment	Irregular vessel wall, 75% stenosis in distal segment	1	3.5*28mmEXCEL balloon stent	98				98
a37	Sinusian bradycardia	99% narrow in the proximal segment	No obvious stenosis	No obvious stenosis	1	3.5*23mm XIENCEPRIME balloon stent	80.5				80.5
a38	Normal ECG	Diffuse lesions in the whole process, long lesions in the mid-distal segment, 80-95% stenosis, D1 thick, 99% proximal stenosis, multiple D2 stenosis with tumor-like expansion	Multiple diffuse lesions throughout the process, 80-90% stenosis in multiple segments	30% stenosis in the proximal segment, 70% stenosis in the posterior descending branch, and 95% stenosis in the posterior branch of the left ventricle	2	3.0*36mmEXCEL ?2.5*33mm Firebird	108	82.5			190.5
a39	T: V2-V4 inverted, V5 biphasic, I, aVL, V6 low level, I degree AVB	99% narrow in the proximal segment	No obvious stenosis	No obvious stenosis		TIVOLI 4.0*30mm balloon stent	120				120
a40	III?aVF is QR	50-60% stenosis in the proximal segment, 80% stenosis in the proximal segment of the stent	The original stent of the distal mid-segment is unobstructed	30% stenosis in the stent	4	2.5*15mm XIENCEPRIME balloon stent	150				150

a41	Sinus reflex, III, aVF raise 0.5-1mm, ST: V5-V6 horizontal depression 0.5-1mm, T: I biphasic, V4 notch	Prolonged lesions in the proximal segment, 50% stenosis, about 70% stenosis of D1 gross opening		70% stenosis in the proximal segment, 90% stenosis in proximal of PL	1	2.75*23mm Firebird balloon stent	63.25				63.25
a42	Sinusian bradycardia	Proximal midsection long lesions, 90% stenosis	40% opening, 80% stenosis in the proximal mid-segment	60% stenosis in the middle of the stent		2.75*38mm Xience Primer balloon stent	104.5				104.5
a43	V1, V2 are QS, V3 is qrS, ST: III, aVF, V1-V4 arched back up 0.5-2mm, T: I, aVL, V2-V5 inverted	100% occlusion in the proximal mid segement	No obvious stenosis	40% stenosis in the proximal middle segment	2	3.35*33?3.5*29 mm Firebird balloon stent	110.55	101.5			212.05
a44	Atrial fibrillation, T: I, aVL biphasic, II, aVF, V5, V6 upside down, ST: V3-V5 arched back up 0.5mm, V1 is QS, V3-V4 is qrS	From the opening to the middle of the long lesion with calcification, more than 90% stenosis, D1 opening and the proximal 80% stenosis	90% stenosis in the middle distal segment	Long lesions near the middle segment with calcification, more than 90% stenosis	2	3.0*28mm 3.0*18EXCEL baloon	84	54			138

a45	AVB, T: V4-V5 biphasic, CRBBB, room early	The original stent in the proximal part is unobstructed	The original segment of the proximal segment is unobstructed	80% stenosis in the proximal and distal segments	4	TIVOLI 3.5*30mm?3.5*21 balloon stent	315	73.5			388.5
a46	Sinusian bradycardia, First degree AVB	Long lesions in the proximal the mid-segment, with a maximum of 70% stenosis	The vessel wall is irregular, the original stent is unobstructed	70% of the most stenosis in the proximal middle segments	2	4.0*29mm Firebird balloon stent	232				
a47	CRBBB	40% narrow in the proximal segment	No obvious stenosis	70% stenosis in the middle segment	1	4.0*33 balloon stent	132				132
a48	Atrial flutter, high voltage left ventricle	95% stenosis in the middle, 70% stenosis in D1	No obvious stenosis	40% stenosis in the middle segment	1	4.0*18 Ji Wei baloon	72				72
a49	Normal ECG	70% stenosis in the middle segemet	80% stenosis in the proximal segment	75% stenosis in the middle segment	1	2.75*29?3.5*21 mm LEPU baloon stent	79.75				79.75
a50	ST: I, aVL, V6 horizontal depression 0.5-1mm, T: I, aVL, V5-V6 inverted	100% occluded in the proximal segment	80% stenosis in proximal segment, 90% stenosis in OM1 opening and proximal segment	Diffuse long lesions throughout the segments, with 80% of the most severe stenosis near the middle segment, 80% stenosis in PLA, 99% stenosis in PDA, collateral blood	2	2.5*33?2.5*29mm Firebird balloon stent	82.5	72.5			155
a51	Sinus bradycardia, low voltage lead	Irregular vessel wall in the proximal middle	90% stenosis of proximal segment	85% stenosis in the proximal segment and 99% restenosis in the stent		3.5*30mm resolute baloon stent	105				105

a52	ST: V1-V3 raised 0.5-1.5mm, T: I, aVL, v4-v6 upside down	99% stenosis in the proximal segment	Middle vessel wall is irregular	No obvious stenosis	1	3.5*18mm Firebird balloon stent	63				63
a53	I degree AVB, T wave change	50% stenosis in the middle segement	Irregular vessel wall, 40% moderate stenosis, 95% the most stenosis in the distal segment	30% stenosis in the proximal middle segments, 40% stenosis in the middle distal segments							
a54	III, aVF is qr, low voltage of limb lead	85% stenosis in the proximal segment and 90% in the distal segment	70% stenosis in the opening and near segment, 80% stenosis in the distal segment	The intimal hyperplasia of the mid-distal stent is mild, 40% stenosis in the distal segment, and 70% stenosis in the proximal segment of PD	2	3.0*18mm Firebird balloon stent	108				108
a55	II, III, and aVF are Qr, ST: the back of the arch is raised 0.5-1.5mm, I, aVL, and V5 are lowered by 1mm horizontally, T: I is flat, V4 is biphasic, and V5-V6 is inverted	Mild stenosis in the proximal stent	Irregular vessel wall	The proximal stent is unobstructed, and the middle and distal segments are 100% occluded	5	TIVOLI 3.5*21mm, 3.0*21mm stent?3.5*12 baloon stent	220.5	63	42		325.5

a56	Sinus bradycardia, T: I, II, aVF, V5, V6 low level, V2-V4 biphasic	90% stenosis in the middle segment	No obvious stenosis	No obvious stenosis	2	2.75*23mm Firebird balloon stent, 2	126.5				126.5
a57	ST: I, II, aVF, V5, V6 reduced by 0.5mm	Mild stenosis in the proximal stent	80% stenosis in the middle distal segment	No obvious stenosis		2.5*24mm resolute baloon stent	60				60
a58	I degree AVB, ST-T change	80% stenosis in the proximal middle segment	40% stenosis in the proximal segment, 90% stenosis in MO	50% stenosis in the distal segment	3	3.5*29?3.5*23mm Firebird?	203	80.5			283.5
a59	Anterior wall myocardial infarction, I, II, aVF, V5, V6 horizontal depression 0.5-1mm, T: I, aVL, V4-V6 inverted, biphasic II, left ventricular high voltage, extended Q-T	Diffuse long lesions in the proximal middle, the most severe 90% stenosis, D2 opening and near 60% stenosis	70% stenosis in the middle segment	70% stenosis in the distal segment, 70% stenosis in the PD, 80% stenosis in PL	1	3.5*28mmEXCEL balloon stent	98				98

a60	T: V4-V6 upside down	40% stenosis in the opening, long lesions in the middle, 80% in the most severe stenosis, 90% in D1 stenosis	40% stenosis in the middle segment, 60% stenosis in the distal segment	Diffuse long lesions throughout, 95% of the most severe stenosis in the proximal segment, 90% stenosis in the middle segment, 90% stenosis in the distal segment, and 85% occlusion in PD	4	3.0*33?2.5*23?3 .5*33?3.5*29mm Firebird balloon stent	99	57.5	115.5	101.5	373.5
a61	Atrial fibrillation, ventricular early, V1-V3 showed QS, ST-T changes	50% of proximal segment, 80% of stenosis with calcification in proximal segment	99% stenosis in the opening, 70% stenosis in the middle segment with calcification	85% stenosis in the proximl middle segment and 80% stenosis in the middle and distal segment	3	2.75*24?4.0*30 mm resolute baloon stent 3.0*33 Firebird	66	120	99		285
a62	Normal ECG	Myocardial bridge in the middle segment, 50% stenosis during contraction	85% stenosis in the middle and distal segment	Irregular vessel wall		3.0*24mmEXCEL balloon stent	72				72
a63	I degree AVB, T: aVL inverted low level	Irregular vessel wall in the proximal middle segment, myocardial bridge in the middle segment	Nearly mid-segmental long lesions, with more than 90% heavy stenosis; 70% gross stenosis in D1	50% stenosis in the middle segment	1	3.5*18mm XIENCEPRIME balloon stent	63				63
a64	ST: I, V4-V6 down 0.5-1mm	Unobstructed stent in the proximal middle segment	Middle stent is unobstructed	90% stenosis in the middle segment, 90% stenosis in the distal segment	5	2.75*18?2.75*24 mm EXCEL baloon	198	66			264

a65	I degree AVB, II, III and aVF exhibit QR	Diffuse lesions in the proximal middle segment, the most severe stenosis 60-70%, the distal stenosis 80%	More than 90% stenosis in the middle segment	The original stent is unobstructed	2	2.75*24mm NANO baloon	66				66
a66	T: aVL inverted, I low level	Diffuse long lesions in the proximal middle segment, the original stent is unobstructed	95% stenosis with tumor-like dilation in the proximal mid-segment	The original stent is unobstructed, and 80% stenosis in the opening of PD	3	2.25*30mm resolute baloon stent	67.5				67.5
a67	Normal ECG	Diffuse lesions, 40% stenosis in the opening, 75% stenosis in the mid-distal segment, and 80% stenosis in the middle segment	80% stenosis in the proximal middle segment and 65% stenosis in the distal segment	95% in the proximal segment, 80% in the middle segment, and 75% in the distal segment	2	3.0*33mm, 3.0*29mm Firebird baloon	99	87			186
a68	Atrial fibrillation, CRBBB, III, aVF is qr, T wave is inverted	99% stenosis in the middle segment	Irregular middle vessel wall	Stent is unobstructed	3	2.25*12???2.75*36 Ji Wei baloon	27				27
a69	T: aVL low level	85% stenosis in the proximal middle segment	The proximal middle vessel is irregular, and 30% thick and stenosis in OM	The vessel wall in the middle and distal segments is irregular, with the most 30% stenosis			99				99

a70	T wave change	Diffuse lesions in the proximal middle segment, with 75% stenosis in the proximal segment and 80% in the distal segment, with visible myocardial bridges	Irregular vessel wall	Irregular vessel wall in the proximal segment	1	3.5*14mmEXCEL balloon stent	49				49
a71	Normal ECG	Irregular vessel wall	Mid-stage subtotal occlusion	Irregular vessel wall, 20-30% stenosis	1	3.0*18mm NANO baloon	54				54
a72	ST: V1-V4 is raised by 1-2mm, ST: II is lowered by 0.5mm, T: I, aVL, V6 is flat, V5 is inverted	100% occlusion in the proximal segment, 80% D1 stenosis	85% stenosis in the proximal segment	40-50% stenosis in the middle segment, 90% stenosis in PL	1	3.0*24mmEXCEL balloon stent	72				72
a73	Normal ECG	No obvious stenosis	85% stenosis in the proximal segment	Irregular vessel wall	1	3.0*12mm NANO baloon	36				36
a74	Sinus bradycardia, low voltage lead, CRBBB	90% stenosis in the D1 proximal segment	No obvious stenosis	No obvious stenosis	1	3.0*14mmEXCEL balloon stent	42				42
a75	Sinus bradycardia, I degree AVB	Irregular vessel wall in the proximal segment	More than 90% stenosis in the middle segment	60-70% stenosis in the proximal segment	1	3.5*15mm XIENCEPRIME balloon stent	52.5				52.5

a76	Sinus bradycardia,	Diffuse lesions in the proximal middle segment, the most severe stenosis is 80%, and the distal stenosis is 70%	90% stenosis at the opening and proximal segment	The proximal stent is unobstructed and 85% stenosis in the distal segment	2	3.0*21mm NANO baloon	126				126
a77	ST: V2-V3 lowered by 0.5mm, T: I, aVL low level, V2-V3 biphasic	Diffuse lesions in the middle segment, the most severe stenosis is 40%, myocardial bridge can be seen near the middle segment, and D1 has a gross stenosis of 85%	No obvious stenosis	Tumor-like expansion can be seen in all segments, without obvious stenosis	1	2.75*18mm resolute baloon stent	49.5				49.5
a78	Ventricular premature beats	D1: 90% stenosis in the proximal segment and 70% stenosis in the distal segment	50% opening, 60% stenosis in the proximal and mid-segment	No obvious stenosis		2.25*24mm resolute stent	78				78
a79	ST: V2-V3 is raised 1-2mm, T: V2, V6, II, III, aVF biphasic, aVL, V3-V5 inverted	90% stenosis in the original stent and the distal segment	No obvious stenosis	The wall of the middle and distal segments is irregular and no obvious stenosis	2	3.5*21mm NANO baloon	73.5				73.5

a80	Normal ECG	Mid-branch lesion, 90% stenosis of main branch, 80% stenosis of D2 opening	No obvious stenosis	No obvious stenosis	1	3.5*15mm resolute baloon stent	52.5				52.5
a81	III, aVF showed QS, left ventricular hypertrophy with strain	70% stenosis in the proximal segement	No obvious stenosis	80% stenosis in the middle segment, 90% stenosis in the distal segment	2	2.75*33 Gu Wei 4.0*25 baloon stent	90.75	100			190.75
a82	ST: V1-V4 is raised by 1-2mm, II, III, aVF is raised by 0.5-1mm, T: I, aVL is inverted, V5 biphasic	Completely occluded above the middle segment	70% stenosis in the opening and proximal segment, 30% stenosis in the middle segment	No obvious stenosis	2	2.75*36mmEXCEL 3.0*29mm Firebird baloon	99	87			186
a83	II, III, aVF showed qR, ST-T changes	90% at the proximal segment, 99% at the middle segment, and 95% at the distal segment	100% occlusion	Subtotal occlusion in the middle segment, and completely occluded in the distal segment	4	T1voh3.5*15mm, 3.5*30mmEXCEL, 3.0baloon, *33?2.75*29mm Firebird baloon	52.5	105	99	79.75	336.25

a84	ST: V3-V6 horizontal depression 0.5mm	Diffuse lesions in the proximal and mid-segment, 90% of the most severe stenosis; and 60% stenosis in the middle and distal segment	The distal wall is irregular, 90% stenosis is in OM1, and OM2 is completely occluded	The wall of the middle and distal segments is irregular, with 30% of the most severe stenosis	1	3.0*36mmEXCEL balloon stent	108				108
a85	T wave change, QT extension	The original stent is completely occluded	The proximal mid-segment stent is unobstructed	80% stenosis in the proximal middle segments	2						
a86	Premature ventricular contractions (PVCs)	Diffuse lesions in the proximal segment, more than 80% stenosis	Long lesions in the proximal middle, 70-80% stenosis	The middle segment is unobstructed and 40% stenosis in the distal segment	1	3.5*36mm NANO	126				126
a87	T: I, aVL biphasic	The most severe stenosis in the proximal and middle segment is 20%, and the distal segment is 90%	Subtotal occlusion in the near segment and 90% stenosis in the distal segment	90% stenosis in the proximal segment, 80% stenosis in the middle segment, 99% in the distal segment, and 30% stenosis in PLA	3	3.0*18mm Firebird baloon, 3.5*36 3.5*18mm NANO baloon	54	126	63		243
a88	Normal ECG	40% stenosis in front of the proximal mid-stent	No obvious stenosis	30% stenosis in the proximal segment	1	3.5*23mm XIENCEPRIME balloon stent	80.5				80.5

a89	Normal ECG	Diffuse lesions throughout, 50% stenosis of the proximal segment, D1 stenosis 60-70%	More than 80% stenosis in the middle segment	40% stenosis in the proximal segment	1	2.25*24mm resolute baloon stent	54				54
a90	Normal ECG	75% of proximal stenosis, middle myocardial bridge	40% stenosis in the middle segment	The proximal vessel wall is irregular, long lesion in the middle segment, and 50% of the most severe stenosis	1	3.5*23mm XIENCEPRIME balloon stent	80.5				80.5
a91	V1-V3 presents QS, ST: V1-V3 raises upward by 1-2mm, and II, III, aVF presents QRs	Middle stent is unobstructed	No obvious stenosis	50% stenosis in the proximal and middle segments, complete occlusion in the distal segment	3	2.5*24mmEXCEL ?3.5*23mm XIENCEPRIME baloon stent	120	80.5			200.5
a92	Normal ECG	95% proximal to the original stent	50% stenosis in the middle segment	50% stenosis in the middle segment	2	3.0*14mmEXCEL balloon stent	84				84
a93	Normal ECG	40% stenosis in the middle segment	The vessel wall of the middle and distal segments is irregular, and 50% stenosis in the OM2	Proximal stent is 100% occlusion	3	3.5*33?3.0*25mmEXCEL balloon stent	115.5	75			190.5

a94	Old inferior myocardial infarction, T: I, aVL, V4 low, V5-6 biphasic	The original stent is unobstructed	90% stenosis of the original stent in the proximal segment and 90% stenosis in the proximal segment of the OM	The original stent is unobstructed, and 80% stenosis in the distal segment	4	2.25*18mm resolute baloon stent	162				162
a95	III, aVF is QRs, low voltage of limb lead	Near-mid stent without stenosis, 50% occlusion at the distal segment	Vessel wall is Irregular, 40% stenosis in the distal segment	Near-segment long lesions, the narrowest 70% with plaque rupture, mid-segment long lesions, 85% of the most severe stenosis	3	2.75*15?3.0*15 Ya Pei baloon stent	82.5	45			127.5
a96	frequent premature ventricular contractions?T: I is low flat, aVL is upside down	Prolonged lesions in the proximal segment, the most severe stenosis is more than 90%	Irregular vessel wall	Irregular vessel wall, 50% stenosis in the middle segment	2	2.0*15?2.75*24 mmEXCEL balloon stent	30	66			96
a97	Normal ECG	Prolonged lesions in the proximal segment, the most severe stenosis is more than 90%	Stent in the proximal mid-segment is unobstructed	Diffuse long lesions in the proximal middle segments, 80% of the most stenosis with signs of plate rupture	5	3.5*18?3.0*25mm balloon stent	252	75			327

a98	T: V4, V5 inverted, V6 biphasic, Q-T interval extended	95% stenosis at opening and proximal segment	No obvious stenosis	No obvious stenosis	1	4.0*23mm XIENCEPRIME balloon stent	92				92
a99	Normal ECG	The most severe stenosis with calcification in the proximal segment is 60%, and D2 stenosis is 65%	85% stenosis in the middle segment, 80% stenosis in the middle distal segment	75% stenosis in PDA, 70% stenosis in PLA	2	2.25*18mm resolute baloon 2.25*15mmEXCEL baloon	81	33.75			114.75
a100	Sinus bradycardia, T: I low, aVL inverted	85% stenosis in the proximal the middle segment	80% stenosis in the middle and distal sections, 65% stenosis in the OM1	No obvious stenosis	2	3.0*158?2.75*18mm resolute baloon stent	474	49.5			523.5
a101	Sinus velocity, V3-V6 biphasic	Mild stenosis in the proximal stent, D1 stenosis 80%	No obvious stenosis	No obvious stenosis	1						
a102	Normal ECG	The stent in the proximal segment is unobstructed, and 80% stenosis in the distal segment	20% stenosis in the distal segment	80% stenosis in the most distal segment	2	3.0*21mm NANO?2.5*25mm Aili balloon stent	63	62.5			125.5
a103	ST: aVF level is reduced by 0.5mm, T: III biphasic	70% stenosis in the middle segment	No obvious stenosis	Unobstructed stent	1	3.5*28 Ji Wei baloon	98				98

a104	ST-T changes, QT prolongs	90% stenosis in the proximal segment, 85% D1 stenosis	95% stenosis in the middle segment	70% stenosis in the proximal segment, 80% stenosis in the distal segment	2	3.5*30mmTIVOLI baloon 2.75*18mmEXCEL baloon	105	49.5			154.5
a105	CRBBB,Atrial premature beat	85% stenosis in the middle segment	No obvious stenosis	Irregular vessel wall	1	2.5*14 Firebird baloon	35				35
a106	ST-T changes	Near 40% stenosis, middle segment myocardial bridge	Open and proximal lesions, 60% of the most stenosis	Long lesions in the proximal middle segments, 85% of the most stenosis	2	3.0*29, 3.5*24mm baloon	87	84			171
a107	Atrial pacing rhythm	95% stenosis in the proximal distal segment	Vessel wall is irregular, 50% stenosis in the opening and middle segments	75% stenosis in the proximal middle segment, unstable plaque	2	4.0*29?3.0*15mm NANO baloon	116	45			161
a108	T wave change	50% stenosis in the middle, 80% stenosis in D1	No obvious stenosis	No obvious stenosis	1	3.0*12mm XIENCEPRIME balloon stent	36				36
a109	Normal ECG	60% stenosis in the opening and the proximal segment, long lesions in the middle segment, and 90% in the most severe stenosis	The proximal vessel wall is irregular and 80% stenosis in OM1	85% stenosis in the proximal segment	1	3.0*24mm resolute baloon stent	72				72

a110	T: V3 biphasic, V4-6 inverted, V4-6 horizontally depressed 0.5mm	Diffuse lesions in the proximal and middle segments, the most severe stenosis 50-80%, subtotal occlusion in the middle and distal segments, 99%	50% opening, 70% stenosis in the proximal and middle segment, 99% stenosis and subtotal occlusion in the middle and distal segment	80% stenosis in the proximal middle segments, 95% stenosis in the middle distal segments	1	3.0*18 Ya pei baloon stent	54				54
a111	Sinus rhythm, ST: I, aVL level is depressed by 0.5mm, T: II is low, I, aVL, V5-V6 is inverted, V4 is biphasic	30% stenosis in the proximal segment, visible myocardial bridge in the mid-distal segment	No obvious stenosis	95% stenosis in PLA	1	2.25*12mm resolute baloon stent	27				27
a112	T wave change	Diffuse lesions in the proximal the middle, the most severe stenosis 80%	The original stent in the proximal segment is unobstructed, and 60% stenosis in the distal segment	Long-range lesions in the middle and distal segments, 40% of the most stenosis	2	3.5*21mm NANO baloon stent	73.5				73.5
a113	T wave change	40-50% stenosis in the proximal segment, 85% stenosis in the middle segment with calcification	40% stenosis in the middle segment	40% stenosis near the middle segment		3.5*15mm NANO baloon stent	52.5				52.5

a114	III, aVF is QR	Diffuse lesions in the proximal middle segment, the most severe stenosis 90%	50% stenosis in the middle segment, the original stent in the middle and distal segment is unobstructed	Irregular vessel wall in the proximal and middle segments	2	3.5*36mm NANO baloon stent	252				252
a115	V1-V4 is QS, ST: V5-V6 is lowered by 0.5-1mm, T: V5, V6 are inverted	The original stent is unobstructed	85% stenosis in the proximal segment and 90% stenosis in the mid-distal segment	30% of the most stenosis in the proximal segment	3	3.5*33mm Firebird baloon, TIVOLI 3.5*21mm baloon	231	73.5			304.5
a116	CRBBB	Diffuse lesions in the proximal middle segment, stenosis 60-70%	60% stenosis in the proximal-distal segment	Diffuse long lesions in the proximal segment, the most severe stenosis 80%, 60% stenosis in the middle segment, 80% stenosis in PD	1	3.5*23mm Firebird baloon	80.5				80.5
a117	Sinus slow, V5-6 low level	D1 proximal stenosis 100%	The original stent is unobstructed	Irregular vessel wall in the proximal segment	3	TIVOLI 31.2*6mm baloon, 2.0*20mm baloon stent	374.4	40			414.4
a118	Normal ECG	80% stenosis in the middle segment	The original stent is unobstructed	60% stenosis in the middle segment, 80% stenosis in the distal segment	2	2.5*38mm XIENCEPRIME balloon stent	95				95
a119	Sinus slow	85% stenosis in the proximal middle segment	50% stenosis in the middle segment	No obvious stenosis	1	3.5*18mm XIENCEPRIME balloon stent	63				63

a120	III?aVF is RS	80% stenosis of the heaviest in the proximal mid-segment	No obvious stenosis	Irregular vessel wall	1	4.0*24mm resolute baloon stent	96				96
a121	Sinus rhythm, atrial paced rhythm, III, aVF showed Qr, T: I, aVL, V5 biphasic	Diffuse lesions in the proximal the middle segment, the most severe stenosis 85%	80% stenosis in the distal segment	30% stenosis in the proximal segment, 85% stenosis in the middle segment, and 95% stenosis in the distal segment	2	2.5*23?3.5*13mm Firebird baloon	57.5	45.5			103
a122	III, aVF is QR	95% stenosis in the proximal middle segment, 65% D1 stenosis	40% stenosis in the distal segment	No obvious stenosis	1	3.5*24mmEXCEL balloon stent	84				84
a123	Limb lead low voltage	60% stenosis in the proximal mid-segment	95% stenosis in the proximal OM1	99% stenosis in the middle segment	4	3.0*36mm EXCEL baloon, 3.0*15, 3.5*18mm Firebird baloon	108	45	63		216
a124	AVB of degree I, Limb lead low voltage, T: I biphasic, aVL inverted	Irregular vessel wall	60% stenosis in the middle segment	80% stenosis in the middle segment, 90% stenosis in the distal segment	1	4.0*15mm NANO baoon	60				60
a125	II, III, aVF is QR, T: V5-6 is inverted	Long lesions in the proximal segment, stenosis in the proximal segment is 60%, and stenosis in the proximal segment is 80%	More than 90% stenosis in the middle segment	The original stent is unobstructed	2	3.0*29mm NANO baloon	261				261

a126	Normal ECG	75% of proximal stenosis	No obvious stenosis	No obvious stenosis	1	4.0*15mm XIENCEPRIME balloon stent	60				60
a127	III, aVF is QR, ST: V1-4 is raised by 1-2.5mm, T: II, III, aVF is inverted	95% stenosis of original stent	100% occlusion in the middle segment	90% re-stenosis in the middle stent	4	3.5*14mmEXCEL balloon stent	196				196
a128	Atrial fibrillation, V1-V2 showed QS, T: II, III, aVF inverted, ST: V4 raised 0.5mm	50% stenosis in the proximal segment	80% reduction in the middle segment	Irregular vessel wall	1	4.0*18mm resolute baloon stent	72				72
a129	Sinus bradycardia	95% stenosis	No obvious stenosis	Irregular vessel wall	1	2.75*24mm resolute stent	66				66
a130	ST: V1-3 is raised by 1-3mm, V5-V6 is horizontally depressed by 0.5mm, T: I, aVL, V6 dual	Original baloon stent mild intimal hyperplasia	Irregular vessel wall	95% stenosis in the proximal segment	2	TIVOLI 4.0*18mm	144				144

a131	III, aVF showed QS, grid: V1-3 arched back raised 0.5-1mm, T: I, aVL, V1-6 inverted	99% stenosis in the proximal segment, 70% stenosis in D1	No obvious stenosis	No obvious stenosis	1	3.5*18mm Firebird	63				63
a132	CRBBB	90% stenosis in the proximal middle segment, 50% stenosis in the middle segment	No obvious stenosis	No obvious stenosis	1	3.5*18mm Firebird baloon	63				63
a133	Sinus bradycardia?ST-T change	80% of proximal D2 stenosis	No obvious stenosis	No obvious stenosis	1	3.5*15mm resolute baloon stent	52.5				52.5
a134	Sinus bradycardia?ST: V4 raised 1.5mm, T: aVL upside down	Irregular middle vessel wall	95% stenosis in the middle segment	Irregular vessel wall in the middle segment	1	3.0*23mm XIENCEPRIME balloon stent	69				69
a135	ST: V1-3 raised 1.5-3mm	Irregular vessel wall in the proximal the middle segment	75% stenosis in the proximal middle segment	30% stenosis in the opening, 99% stenosis in the proximal segment, and 75% stenosis in the distal segment	2	3.5*36?3.5*28mmEXCEL balloon stent	126	98			224

a136	ST-T Change	40% stenosis in the proximal segment, 99% stenosis in the middle segment, 90% stenosis in D2, and 99% stenosis in D3	40% stenosis in the proximal segment and 99% stenosis in the distal segment	85% of the most stenosis in proximal middle segment	2	3.0*15mm Firebird?3.0*36 mmEXCEL balloon stent	52.5	108			160.5
a137	Left ventricular high voltage	The most severe stenosis in the opening and proximal segment is 85%, the stenosis in the distal segment is 80%, and the stenosis in D1 is 90%.	No obvious stenosis	The original stent is unobstructed, and 30% stenosis in the distal segment	3	3.5*24mm Resolutue baloon, 3.0*36 EXCEL baloon	84	108			192
a138	Sinus rhythm, ST: III, aVF level is 0.5mm lower, ST: I, aVL, V1-V6 is raised 0.5-4.5mm, aVL, V4-V6 is QS, III, aVF biphasic	Diffuse lesions in the proximal segment, with the most severe stenosis 60%, complete occlusion from the proximal segment, and 75% stenosis in the D1 opening	Irregular vessel wall in the middle and distal segments	Irregular vessel well in the proximal segment, 50% stenosis in the posterior descending opening, and 70% stenosis in the proximal segment	1	3.5*28mmEXCEL balloon stent	98				98

a139	Normal	Irregular vessel wall	30% reduction in the opening	50% stenosis in the proximal middle segments, 40% stenosis in the middle segment, and 85% stenosis in the distal segment	1	4.0*30mm resolute baloon stent	120				120
a140	Sinus velocity, T: II, III, aVF, V5, V6 biphasic	90% stenosis in the middle segment, the original stent in the middle and distal segment is unobstructed	Irregular vessel wall	No obvious stenosis	2	TIVOLI 2.75*25mm baloon	68.75				68.75
a141	T: aVL inverted	95% stenosis in the middle segment	The middle and distal segment are completely occlusion	60% stenosis in the middle and distal segments	2	2.75*28?3.0*28 mmEXCEL balloon stent	77	84			161
a142	T: aVL two-phase, V5 notch	Diffuse lesions in the proximal middle segment, the most severe stenosis is 95%, and the proximal D1 stenosis is 80%	60% stenosis in the proximal segment of OM1	Nearly 60% stenosis in the proximal segment	4	3.5*18?3.0*28?3 .0*36mmEXCEL? 2.75*36NANO	63	84	108	99	354
a143	V1-V2 showed QS, ST: V1-V2 lifted up 0.5mm, T: V4-5 biphasic	Subtotal occlusion in the proximal middle segment	No obvious stenosis	No obvious stenosis	1	3.0*13mm Firebird baloon	39				39

a144	Sinus bradycardia?CRBBB	60% stenosis in the proximal-mid segment, D1 90% stenosis	90% stenosis in the middle segment	50% stenosis in the middle and distal segments	1	3.5*15mmEXCEL balloon stent	52.5				52.5
a145	ST: the back of II, III, aVF, V4 is raised 0.5-1mm	80% stenosis in the proximal segment, 60% stenosis in the D1 opening	85% stenosis in proximal segment	More than 90% of the stenosis in the proximal segment	2	TIVOLI 3.0*35mm baloon, 2.5*36 NANO baloon	105	90			195
a146	CRBBB	60% stenosis in the proximal segment	Irregular vessel wall	50% stenosis in the proximal segment and 80% stenosis in the distal segment	1	2.5*23mm XIENCEPRIME balloon stent	57.5				57.5
a147	Normal ECG	Distortion of the proximal segment with 60% stenosis	Considering the unstable plaque, 70% stenosis is in the middle and distal segment	50-60% stenosis in the middle segment	1	3.0*33mmEXCEL balloon stent	99				99
a148	Ventricular premature beats, ST: V5, V6 horizontal depression 0.5-1mm, V2 elevation 0.5mm, T: V3-6 inverted, V1, V2 showed QS	60% stenosis of the distal part of the original stent	More than 80% stenosis in the proximal stent	90% stenosis in the middle segment	4	TIVOLI 4.0*21mm baloon, 3.0*24NANO baloon	84	72			156
a149	Normal ECG	95% narrow in the middle, 60% narrow in D2 opening	50% stenosis in the proximal and middle segments	50-60% stenosis in the middle segment	2	2.25*12mm XIENCEPRIME balloon stent	54				54

a150	I degree AVB	Mild endometrial hyperplasia in the proximal-middle segment, with 85% stenosis in the mid-distal segment and 90% stenosis in the distal segment	40% stenosis in the distal segment	50% stenosis in the middle segment, 80% stenosis in middle segment of PD2		3.0*14mm XIENCEPRIME?2.25*14mm resolute baloon	42	31.5			73.5
a151	CRBBB?I, aVL is QS	95% of proximal stenosis with calcification	100% occlusion in the proximal and middle segments	80% stenosis in the proximal and distal segments	2	3.0*29, 3.5*33mm Firebird baloon	115.5	87			202.5
a152	T wave change	Irregular vessel wall	80% stenosis in the middle and distal segments, 60% stenosis in the proximal segment of OM	Irregular vessel wall	1	3.0*12mm XIENCEPRIME balloon stent	36				36
a153	T: III upside down, aVF low level	95% stenosis in the proximal middle segment, myocardial bridge in the middle and distal segment	75% stenosis in the middle segment	70% narrow in the middle segment	1	3.0*29mm NANO baloon	87				87
a154	I degree AVB	95% stenosis in D segment	30-40% stenosis in the distal segment	No obvious stenosis	1	3.0*15mmTIVOL baloon	45				45

a155	V1-V3 showed QS, ST: V1-V3 raised 0.5-2mm upward, V5, V6 horizontally depressed 0.5-1mm, T: I, aVL, II, aVF low level, V5-6 biphasic	No obvious stenosis	Irregular vessel wall	75% stenosis in the middle segment of PDA	1	2.25*12mm XIENCEPRIME balloon stent	27				27
a156	Normal ECG	No obvious stenosis	99% stenosis in the opening	No obvious stenosis	1	2.25*38mm XIENCEPRIME balloon stent	85.5				85.5
a157	sinus bradycardia?Premature ventricular contractions (PVCs)?ST-T change	100% occlusion from the proximal segment	Subtotal obstruction in the distal segment, 90% stenosis in the opening of OM, and 99% stenosis in the proximal segment	95% stenosis of the opening, 95% of the most stenosis in the middle segment with calcification	1	2.5*13mm Firebird baloon	32.5				32.5
a158	IIIQS, aVF is QRs, ST: III, aVF arch dorsal raise 0.5mm, I, aVL level lower 0.5mm	80% stenosis in the middle segment	80% stenosis in the middle segment	100% occlusion in the middle segment		3.0*36mm baloon stent	108				108

a159	ST: Level II, III, aVF reduced by 0.5mm	More than 90% stenosis in the proximal segment	The original stent is unobstructed	60% stenosis in the proximal segment, 40% stenosis in the middle segment	3	3.5*28?4.0*18mmEXCEL balloon stent	98	144			242
a160	V1-V5 showed QS, ST: V1-V3 raised 0.5-2mm, T: I, aVL inverted	Complete occlusion was occurred in the proximal segment after D1, 70% stenosis of long lesions in the D1	70% stenosis in the proximal segment of OM1	Nearly 80% localized stenosis	3	2.25*20?2.5*30?2.5*30mm resolute baloon stent	45	67.5	75		187.5
a161	V1-V2 showed QS, ST: V1-V3 raised 0.5mm, T: I low flat, aVL inverted	99% stenosis in the proximal segment	80% stenosis in the middle and distal segments	No obvious stenosis	3	2.5*25 baloon stent, 3.5*29mm Le Pu baloon, 3.5*29mmTIVOLI baoon	62.5	101.5	101.5		265.5
a162	sinus bradycardia	More than 95% stenosis in the middle segment	More than 70% stenosis in the distal segment	Irregular vessel wall	1	3.0*24 baoon stent	72				72
a163	ST: V5-V6 level down 0.5mm, T: II, v5-6 low level	Mild intimal hyperplasia in the original stent	85% stenosis in the middle segment of OM	Small, no stenosis	2	2.25*18mm XIENCEPRIME balloon stent	81				81
a164	Limb lead low voltage, ventricular early, T wave high sharp	70% stenosis in the proximal segment, 95% stenosis in the D1 opening	Irregular vessel wall	75% stenosis in the middle segment	3	2.5*18mm ENDEVOR baloon stent, 2.5*15mm Mei dun Li baoon, 3.0*24mm endeavor baloon	40.5	37.5	72		150

a165	Atrial premature beats and pairs,Limb lead low voltage, CRBBB	80% stenosis in the proximal middle segment	The original stent is unobstructed, 95% stenosis in the middle and distal segments	95% stenosis segment in the middle and distal segments	4	2.5*38?2.25*12 mm XIENCEPRIME baloon stent	190	54			244
a166	III, aVFQS, II is QRs, T: II, III, aVF upside down	50% stenosis in the middle segment	100% occlusion in the middle and distal segments	95% stenosis in the proximal segment	3	3.0*35mm TIVOLI, baloon 2.25*28mmXIENCE, 2.75*21LEPU baloon	63	57.75			120.75
a167	aVF shows QS, T wave changes	50% stenosis in the middle segment	Irregular vessel wall, mild stenosis	Subtotal occlusion in the middle segment	1	2.75*15mm TIVOLI	41.25				41.25
a168	Sinus reflex, I degree AVB, T changes	95% stenosis of the original stent, 70% stenosis in the middle segment, 70% stenosis in the distal segment and 90% stenosis in the D1	50% stenosis in the proximal segment, 85% stenosis in the middle and distal segments	50% stenosis in the proximal segment, 70% in the middle segment, and 50% in the distal segment	2	3.5*15mm TIVOLI baloon, 3.5*14m2mNANO baloon	52.5	-49			3.5
a169	Normal ECG	90% stenosis under the D bifurcation	No obvious stenosis	Irregular vessel wall	1	4.0*12mm solute balloon stent	48				48
a170	IRBBB	80% stenosis in the proximal segment	Irregular vessel wall	Irregular vessel wall	1	2.75*28mmEXCEL balloon stent	77				77

a171	Limb lead low voltage	Irregular vessel wall	80% stenosis in the mid-distal segment and 60% stenosis in the proximal OM segment	Irregular vessel wall	2	3.0*25mm TIVOLI baloon, 2.25*12mm XIENCE baloon	75	27			102
a172	CRBBB,Atrial premature beat	95% stenosis in the proximal segment	Irregular vessel wall	60% stenosis in the middle segment	2	3.0*12mm TIVOLI baoon, 2.25*14mm ENDEAVOR baloon	36	31.5			67.5
a173	T: V1-V6 is low, QT interval is prolonged	95% stenosis in the proximal middle segment	No obvious stenosis	No obvious stenosis	1	3.0*21mmLEPU stent	63				63
a174	Left ventricular high voltage	99% stenosis in the proximal segment	More than 80% stenosis in the middle and distal segment	Nearly 60% stenosis in the proximal segment	2	4.0*15mmLEPU stent?3.0*14mm EXCEL	60	42			102
a175	V2-V3 showed QRs, ST-T changes	99% stenosis in the proximal middle segment	85% stenosis in the middle and distal segment	Nearly 85% stenosis in the proximal middle segments	2	2.75*21?3.0*15 mmLEPU stent	57.75	45			102.75
a176	II, III, aVF are QR, V4-6 are qRs, ST: II, III, aVF, V5, V6 horizontal depression 0.5-1mm	More than 90% stenosis in the proximal segment	80% of the most stenosis in the proximal segment	100% occlusion in the middle segment	2	3.0*21mmLEPU stent?3.0*35mm sheet music stand stent	63	105			168
a177	III?aVF shows QR	50% stenosis in the proximal segment	Irregular vessel wall	90% stenosis in the proximal and middle segments and 40% stenosis in the distal segment	1	4.5*20Libertte stent	90				90

a178	ST-T Change	80% stenosis in the opening and proximal segment, 95% stenosis in the middle segment	100% occlusion in the middle segment	75% stenosis in the proximal segment, 60% stenosis in the middle segment, and 70% stenosis in the distal segment	1	2.5*29mm Firebird	72.5				72.5
a179	ST: II, III, aVF increased 0.5-2mm, ST: I, aVL horizontal depression 1-1.5mm, T: II, III, aVF inverted, III degree AVB	More than 50% stenosis in the proximal segment	Irregular vessel wall	100% occlusion in the proximal segment	1	3.5*28mmEXCEL balloon stent	98				98
a180	Ventricular premature beats, low voltage lead	70% stenosis in the proximal segment, stenosis 95% in D1	Irregular vessel wall	75% stenosis in the middle segment	2	2.5*15mm Mei Li Dun,2.5*18ENDEAVOR	37.5	45			82.5
a181	Sinus rhythm, atrial early, ST: V3 approximately 1mm lower horizontally, ST: I, aVL horizontally lower 0.5-1mm, QT prolonged	80% stenosis in the middle distal segment	60% stenosis in the middle segment	Irregular vessel wall	1	3.5*29mm Firebird	101.5				101.5

a182	Normal ECG	More than 90% of the stenosis in the proximal segment	95% stenosis in the middle and distal segments	Irregular vessel wall	2	2.5*38?2.5*38mm XIENCEPRIME balloon stent	95	95			190
a183	T wave change	More than 80% stenosis in the proximal segment with the longest lesion	50-60% stenosis in the middle and distal segments	75% stenosis in the middle segment	1	TIVOLI 3.5*25mm	87.5				87.5
a184	Sinus reflex, Atrial premature beats, ST: V1-V3 obliquely elevated 1-2mm.	99% in the proximal segment	No obvious stenosis	No obvious stenosis		3.0*28mmEXCEL balloon stent	84				84
a185	Extensive ST-T changes	70% in the proximal segment	99% stenosis in the middle segment, 85% stenosis in the distal segment	50% stenosis in the proximal segment and 80% stenosis in the distal segment	2	3.0*18?2.5*24?mmEXCEL balloon stent	84	60			84
a186	Limb lead low voltage	90% stenosis in the proximal middle segment, 80% stenosis in the distal segment, 75% stenosis in D1, and 80% stenosis in D2	85% stenosis with calcification in the proximal segment and 95% stenosis in the middle segment	50% stenosis with calcification in the middle segment	2	TIVOLI 2.5*21?2.25*18 mm	52.5	40.5			93

a187	ST-T change	More than 90% of the stenosis in the proximal segment	80% stenosis in the middle segment	99% stenosis in the proximal and middle segments	1	TIVOLI 3.0*15mm	45				45
a188	T: I, aVL, V4, V5, V6 low level, I degree AVB	60-70% stenosis in the proximal segment, 50% stenosis in the distal segment, and 80% stenosis in the D1 segment	75% stenosis in the proximal segment, 95% of the most stenosis in the middle segment	40% stenosis in the proximal and distal segments, 85% stenosis in the middle and distal segments, 95% stenosis distal segment		3.0*14mmEXCEL balloon stent	42				42
a189	Normal ECG	More than 80% of stenosis in the proximal segment with calcification??	Irregular vessel wall	Irregular vessel wall	1	3.5*33mmEXCEL balloon stent	115.5				115.5
a190	Normal ECG	More than 80% stenosis in the proximal segment, 80% stenosis in the middle segment????80%	No obvious stenosis	No obvious stenosis	2	3.0*18?4.0*18mm Firebird	54	72			126
a191	Extremely clockwise transposition, T: I, aVL inverted	60-70% stenosis in the proximal mid-segment	More than 80% stenosis in the middle segment	Almost 100% occlusion in the middle segment	1	4.5*20Libertte stent	90				90
a192	Normal ECG	50% stenosis in the proximal segment and 80% stenosis in the distal segment	70% stenosis in the middle and distal segments	30% stenosis in the proximal segment and 40% in the middle segment	1	2.25*24mm Firebird	54				54

a193	Sinus bradycardia	99% stenosis in the proximal stent	50% stenosis in the middle segment	50% stenosis in the proximal and middle segments	2	3.5*15mmLEPU stent	52.5				52.5
a194	T: Inverted III, aVF biphasic	99% stenosis in the proximal middle segment	90% stenosis in the proximal segment	Unobstructed stent	2	3.0*12mmLEPU stent	36				36
a195	Sinus bradycardia?I degree AVB, T change	The original stent is unobstructed, and 60% stenosis in the segment	Irregular vessel wall, mild stenosis	75% stenosis in the middle segment	2	3.5*33mmEXCEL balloon stent	115.5				115.5
a196	CLBBB	Irregular tube wall, mild stenosis	85% stenosis in the middle segment	Irregular vessel wall, mild stenosis	1	3.0*14mmEXCEL balloon stent	42				42
a197	Atrial fibrillation with ventricular block, T changes	90% stenosis in the local stent??90%	100% occlusion in the proximal segment	95% of the most stenosis in the proximal and middle segments	4	2.75*18mm Firebird?2.75*14 mmEXCEL	99	77			176
a198	Left ventricular high voltage, ST-T changes	More than 80% stenosis in the middle segment	No obvious stenosis	30% stenosis in the proximal segment	1	3.0*18mmEXCEL balloon stent	54				54
a199	V1-V2 shows QS, ST: V1-4 arched, raised 1-1.5mm, I degree AVB	Stent unobstructed	80% stenosis in the middle segment	60% stenosis in the opening	1	3.0*18mmEXCEL balloon stent	54				54

a200	Inferior myocardial infarction, intraventricular block, ST-T changes	75% stenosis in the proximal segment	Irregular vessel wall	100% occlusion in the proximal segment	1	3.5*33mmEXCEL balloon stent	115.5				115.5
a201		60% stenosis in the proximal segment	85% stenosis in the proximal segment	Irregular vessel wall	1	2.75*21mmTIVOL	57.75				57.75
a202	Left ventricular high voltage	Stent unobstructed	More than 95% stenosis in the proximal segment	85% stenosis in the proximal segment	3	2.5*36?3.5*24mmEXCEL balloon stent	180	84			180
a203	Normal ECG	No obvious stenosis	50% stenosis in the middle segment	80% stenosis in the proximal segment	1	3.5*35mmTIVOL stent	122.5				122.5
a204		80% stenosis under the opening	Irregular vessel wall	Irregular vessel wall	2	3.0*28?3.5*33mmEXCEL balloon stent	84	115.5			199.5
a205	I degree AVB	The proximal stent is unobstructed	90% stenosis in the middle segment	60-70% stenosis in the proximal segment	2	Le Pu 2.5*21mm stent	52.5				52.5
a206	ST: The V1-4 bow raises upward 0.5-2mm	More than 85% stenosis in the proximal middle segment	40-55% stenosis in the proximal segment, 99% in the middle segment	80% stenosis in the proximal and middle segments and 70% stenosis in the distal segment	2	2.75*18?2.75*14mmEXCEL balloon stent	49.5	38.5			88
a207	T: Inverted III, aVF biphasic	More than 99% stenosis in the proximal middle segment	90% stenosis in the proximal segment	Unobstructed stent	2	3.0*12mmLEPU stent	36				36

a208	T: Inverted III, aVF biphasic	85% stenosis in the proximal segment and 80% stenosis in the distal segment	No obvious stenosis	85% stenosis in the middle and far segments	1	4.0*24mmEXCEL balloon stent	96				96
a209	ST: V5-V6 horizontal depression 0.5mm, T: aVL low level, V6 biphasic	The original stent is unobstructed	75% of proximal stenosis in the proximal segment	Irregular vessel wall	2	2.5*15mmLEPU?2.5*18mmTIVOL	37.5				37.5
a210	Sinus bradycardia	60% stenosis in the proximal segment and 80% stenosis in the distal segment	No obvious stenosis	No obvious stenosis	1	4.0*15mm xience	60				60
a211	Sinus bradycardia	99% stenosis in the proximal stent	50% stenosis in the middle segment	50% stenosis in the proximal and middle segments	1	3.0*15mmLEPU stent	45				45
a212	T: III upside down, aVF low level	More than 85% of stenosis in the proximal segment	No obvious stenosis	No obvious stenosis	1	??4.0*18mm	72				72
a213	Normal ECG	80% heaviest stenosis	30% stenosis in the opening	The original stent is unobstructed, and 30% stenosis in the distal segment	2	3.5*24mmEXCEL balloon stent	168				168
a214	ST-T Changes	More than 90% of the stenosis in the proximal segment	80% narrow in the middle segment	99% stenosis in the proximal segment	1	3.0*21mmTIVOL	63				63

a215	Sinus velocity	75% stenosis in the proximal segment, D100% occlusion	70% stenosis in the distal segment	90% stenosis in the proximal segment	1	2.25*24mm stent	54					54
a216	I degree AVB	50% stenosis in the proximal segment, patency of the original stent in the distal segment, and 95% stenosis in the D1 segment	80% of the most stenosis	85% stenosis in the proximal and middle segments	2	3.5*29mm Firebird	101.5					101.5
a217	ST: I, V4-V6 horizontal depression 0.5mm, T: aVL, V3 reverse phase, V4-V6 two phase	100% occlusion under the opening	50% stenosis in the proximal segment, 60% stenosis in OM	90% stenosis of the original stent in the middle segment and 50% stenosis in the distal segment	0							
a218	Low voltage in limb lead, QRs in II and aVF	60% stenosis in the proximal segment	70% stenosis in the middle segment	Nearly 85% stenosis in the proximal and middle segments	1	4.0*15mm LEPU	60					60
a219	Sinus bradycardia?Atrial premature beats, ST: V3-4 lowered 0.5-1.5mm, T: V3 biphasic, aVL, V4-6 inverted	90% stenosis in the proximal middle segment	100% occlusion in the middle segment	Irregular vessel wall	1	3.0*15mm NANO	45					45

a220	Sinus rhythm, I degree AVB	90% stenosis in the proximal middle segment	60-70% stenosis in the proximal segment	Irregular vessel wall in the whole segments, 50-60% stenosis	1	2.5*23 Firebird	57.5				57.5
a221	T wave change	80% stenosis in the proximal segment	No obvious stenosis	No obvious stenosis	1	4.0*18mm LEPU	72				72
a222	T: II, III, aVF upside down	No obvious stenosis	No obvious stenosis	Nearly 100% occlusion in the proximal segment	1	3.0*35mmTIVOL	105				105
a223	Normal ECG	Myocardial bridge in the middle distal segment	85% stenosis in the middle and distal segment	Irregular vessel wall	1	2.75*15mmTIVOL	41.25				41.25
a224	Sinus rhythm, left ventricular high voltage	100% occlusion in the proximal segment	100% occlusion in the proximal segment	95% stenosis in the proximal segment	2	3.5*28?3.5*24mmEXCEL balloon stent	98	84			98
a225	Sinus rhythm, left ventricular hypertrophy, ST-T changes	80% stenosis in the middle segment	50-60% stenosis in the middle segment	50% stenosis in the proximal segment	2	2.5*18 Firebird?2?	45				45
a226	ST: I, V1-V4 level down by 1-3mm, T: aVL, V3 bi-phase, V4-V6 upside down	100% occlusion in the proximal segment	85% stenosis in the middle segment	Irregular vessel wall, moderate stenosis	1	2.25*24mm Mei Li dun?2.5*38mm XIENCEPRIME	54	95			149

a227	Sinus bradycardia?Limb lead low voltage, T: I, aVL, V1-5 high tip	80% stenosis in the proximal segment	The original stent is unobstructed	50-60% stenosis in the proximal and middle segments	2	2.5*33 Firebird	190				190
a228	Left ventricular high voltage, T wave change	Irregular tube wall, mild stenosis	95% stenosis in the middle segment	Occlusion in the middle segment	1	2.5*36mm LEPO	90				90
a229	Normal ECG	95% stenosis in the proximal segment	99% stenosis in the proximal and middle segments	60% contraction in the proximal segment, 50% elongation in the mid-distal segment, and 60% stenosis in the PDA	3	2.5*33?3.0*29mm Firebird?2.5*14 mmEXCEL	82.5	87	35		204.5
a230	ST-T changes, QT prolongs	99% stenosis in the distal segment	Irregular vessel wall	40% stenosis in the proximal and middle segments	1	3.5*18mm Firebird	63				63
a231	V1-V3 showed QS, T: I was low, aVL was inverted	100% occlusion in the proximal segment	100% occlusion in the middle segment	30% stenosis in the opening	4	2.25*30mm Resolutue?3.5*29?5*29?2.7mm Firebird?3.0*36EXCEL	67.5	101.5	145	108	422
a232	Normal ECG	More than 80% of the stenosis in the proximal segment	No obvious stenosis	No obvious stenosis	1	??3.5*15mm	52.5				52.5
a233	Normal ECG	Irregular vessel wall	80% stenosis in the proximal segment of OM	Irregular vessel wall	1	TIVOLI 3.0*15mm	52.5				52.5

a234	Limb lead low voltage	50% stenosis in the proximal segment	No obvious stenosis	95% stenosis in the proximal segment	1	TIVOLI 4.0*30mm	120				120
a235	CLBBB	99% stenosis in the middle segment, 80% stenosis in D1	No obvious stenosis	Irregular vessel wall	1	2.75*13mm Firebird	35.75				35.75
a236		60% stenosis in the proximal middle segment, 60% stenosis in D1	90% stenosis in the distal segment of stent	99% stenosis in the early stent	4	2.5*14mmEXCEL ?3.0*13mm Firebird	35	39			74
a237	Sinus bradycardia?CRBBB	80% stenosis in the proximal middle segment	No obvious stenosis	No obvious stenosis	1	3.0*15mm LEPU	45				45
a238	Atrial fibrillation, T change	50% stenosis in the proximal segment	No obvious stenosis	Unobstructed stent	1						
a239	Sinus rhythm, ST-T changes	90% stenosis in the middle distal segment	50% stenosis in the proximal segment	60% stenosis in the middle segment, 50% stenosis in the distal segment	1	2.75*33 Firebird	90.75				90.75
a240	Normal ECG	90% stenosis in the proximal middle segment	75% of the most stenosis, 95% of the most stenosis in OM	50% stenosis in the proximal and middle segments, 60% stenosis in the middle segment, and 50% stenosis in the distal segment	1	3.0*30mm resolute stent	90				90

a241	T: aVF upside down, I low level	More than 90% of stenosis in the proximal segment, 50-60% of D1 stenosis	60% stenosis in the middle segment	60-70% stenosis in the middle segment, more than 85% stenosis in the proximal and distal segments		3.5*28mmEXCEL balloon stent	98				98
a242	I degree AVB, low voltage of limb lead, T: V5, V6 low level	60% stenosis in the opening, 85% stenosis in the proximal middle segment	40% stenosis in the middle segment	Irregular vessel wall	1	3.0*30mm resolute stent	90				90
a243	Sinus bradycardia?I degree AVB	80% stenosis in the proximal middle segment and 70% stenosis in the distal segment	90% stenosis in the proximal and middle segments	80% stenosis in the proximal segment	3	2.5*13mm Firebird?3.0*30 mmEXCEL?3.5*2 5mmTIVOLI stent	32.5	90	87.5		210
a244	Normal ECG	60% stenosis in the proximal segment, 90% stenosis in the mid-distal segment	Irregular vessel wall	Irregular vessel wall	1	2.75*13 Firebird stent	35.75				35.75
a245	Normal ECG	99% stenosis in the proximal middle segment, 99% D1 stenosis	No obvious stenosis	No obvious stenosis	1	3.5*36mmEXCEL balloon stent	126				126
a246	Left ventricular high voltage	The original stent in the middle segment is unobstructed, and 70% stenosis in the distal segment	No obvious stenosis	No constant plaques in the proximal segment with 85% stenosis	2	2.5*14mm resolute stent	70				70

a247	Normal ECG	95% stenosis in the proximal segment	No obvious stenosis	No obvious stenosis	1	3.5*18mmTIVOL	63				63
a248	Extensive anterior myocardial infarction	100% occlusion in the proximal segment	95% stenosis in the proximal and middle segments	70% stenosis in the middle segment	2	3.5*24?2.5*24mmEXCEL balloon stent, 100	84	60			144
a249	Normal ECG	50-60% stenosis in the proximal middle segment	100% occlusion in the middle segment	50% stenosis in the proximal and middle segments, 50% stenosis in PD	1	2.5*18mm resolute stent	45				45
a250	T?II?III?aVF upside down	50-60% stenosis in the middle segment	Subtotal occlusion in the middle and distal segments, the most of stenosis in the proximal segment of OM	30-40% stenosis in the proximal segment	1	Le Pu, 2.75*21mm	57.75				57.75
a251	Atrial premature beats with intraventricular differential conduction, T: I, aVL, V6 inverted, II, V5 low	80% stenosis in the proximal middle segment	Subtotal occlusion in the middle and distal segments	Nearly 100% occlusion in the proximal segment	1	Ji Wei, 2.5*28	70				70
a252	Normal ECG	85% stenosis of the distal segment of the original stent, 95% stenosis in D1	50% stenosis in the middle segment	60% stenosis in the middle segment	2	2.25*12mm Mei Li Dun stent	54				54

a253	Normal ECG	The original stent is unobstructed, and 40% stenosis in the distal segment	No obvious stenosis	80% stenosis in the proximal and middle segments	3	3.5*36mmEXCEL balloon stent	126				126
a254	Sinus bradycardia?I degree AVB, T: aVL inverted, I, V6 biphasic	90% stenosis in the proximal segment	95% stenosis in the proximal segment	95% stenosis in the proximal and middle segments	3	3.0*36?3.0*29?3.5*12mmLEPU stent	108	87	42		237
a255	Normal ECG	More than 85% stenosis in the proximal middle segment	No obvious stenosis	No obvious stenosis	1	4.5*12 BostonTAXUS stent	54				54
a256	Left ventricular high voltage	99% stenosis in the proximal segment	No obvious stenosis	30-40% stenosis in the mid-distal segment	1	4.5*16 BostonTAXUS stent	72				72
a257	T?II?III?aVF dual phase, V5-V6 low level	60-70% stenosis of original stent, 95% stenosis in D1	The original stent is unobstructed	The original stent is unobstructed, and 50-60% stenosis in the distal segment	4	3.5*18mm Firebird balloon stent	63				63
a258	Atrial fibrillation with long RR interval	80% stenosis in the proximal segment and 60% restenosis in the original stent	70% stenosis in the middle segment	100% occlusion in the proximal segment	2	Le Pu 3.5*24mm	84				84
a259	T: V3-6 upside down	60-70% stenosis in the proximal middle segment	Irregular vessel wall in the proximal segment	90% stenosis near the middle segment	1	2.25*23mm Abbott balloon stent	51.75				51.75

a260	Sinus bradycardia?ST-T changes	The original stent is unobstructed, 80% stenosis in the middle segment	40% stenosis in the middle segment	No obvious stenosis	2	3.0*21mm Yi sheng stent	63				63
a261	Sinus rhythm, V1-V3 is QS, ST: V1-V3 is approximately 1.5-2mm higher than the back of the arch	99% stenosis in the proximal middle segment	40% stenosis in the middle segment	No obvious stenosis	1	Le Pu 2.75*18mm	49.5				49.5
a262	Forearm myocardial infarction	60% stenosis of opening, 70% stenosis of proximal segment, the original stent is unobstructed	90% stenosis in the middle segment	85% stenosis in the proximal segment	3	Le Pu 2.5*21mm?4.0*23mm Firebird	47.25	92			47.25
a263	T wave change	No obvious stenosis	70% stenosis in the middle segment, 80% stenosis in OM	No obvious stenosis	1	Yi Sheng 3.0*15mm	45				45
a264	Atrial fibrillation, T change	The proximal segmental is total occlusion	95% stenosis in the middle and distal segments	90% stenosis in the middle segment	1	3.5*30mm Mei Li Dun stent	105				105
a265	T?II?aVF low level	85% stenosis in the proximal middle segment, 90% stenosis in D1	No obvious stenosis	No obvious stenosis	2	2.5*13mm Firebird?2.5*30Resolute stent	32.5	75			107.5

a266	Normal ECG	90% of the most stenosis in the proximal segment, 70% narrowed in D1	Irregular vessel wall	Irregular vessel wall in the whole segments	2	3.0*30mm resolute?3.5*12 NANO	90	42			132
a267	T wave change	95% stenosis in the proximal segment	70% stenosis in the middle and distal segments	100% occlusion in the distal segment	1	2.5*29mm Firebird	72.5				72.5
a268	ST: V1 is raised 1mm, V4-V6 is horizontally depressed by 1-1.5mm, T: V4-V6 is inverted	85% stenosis in the proximal middle segment, 90% stenosis in D1	100% occlusion from the proximal segment	90% contraction in the proximal segment, 95% stenosis in the middle segment, 99% contraction in the distal segment, 99% stenosis in PDA	4	2.5*18?2.75*23?2.75*18mm Firebird?3.5*14Excel	45	63.25	49.5	49	206.75
a269	ST: V1-V6 level increased by 0.5-3mm, V1-V5 is QS, T: V6 reversed	The original stent has mild intimal hyperplasia, and 50% stenosis in the distal segment	85% stenosis in the proximal segment	30% of the most stenosis	2	2.75*12mm balloon stent	33				33
a270	Normal ECG	85% stenosis of the opening and the proximal segment, 80%, 80%, 90% stenosis in D1, D2, D3 respectively	50% stenosis in the proximal segment	70% stenosis in the proximal segment, 60% stenosis in the middle segment	2	3.5*15?3.5*12mm NANO	52.5	42			94.5

a271	III?aVF shows QR, T wave changes	50% stenosis in the proximal segment and 40% stenosis in the distal segment	Opening, 40% stenosis in the opening, proximal and middle segments	80% stenosis in the distal segment	1	3.0*30mm Resolutue	90				90
a272	ST: the dorsal arch of the V1-V3 arch is raised 0.5-2mm, the limb leads are low, T: I, aVL, V2-6 is inverted	The most stenosis is 95%, 70% stenosis in D1	40% stenosis in the opening and the proximal segment, 99% stenosis in MO3, 50% stenosis in the middle and distal segments	80% of the most stenosis	2	2.75*23mm Firebird?2.25*14 Resolute stent	63.25				63.25
a273	T: V5, V6 low level	50% stenosis in the proximal mid-segment and 60% stenosis in D1	99% stenosis in the middle and distal segment	Irregular vessel wall	1	2.25*24mm resolute stent	54				54
a274	CRBBB	No obvious stenosis	50% stenosis in the middle and distal segments	85% stenosis in distal segment	1	3.0*19coroflex stent	57				57
a275	ST-T change	50-60% stenosis in the proximal segment of the original stent, 80% stenosis in the distal segment	30-40% stenosis in the middle segment	50-60% stenosis in the distal segment	2	Le Pu 3.5*15mm	52.5				52.5

a276	Limb lead low voltage, T: V4-V6 upside down, ST: V5, V6 horizontal depression 0.5mm, I degree AVB	95% stenosis from the proximal segment	99% stenosis in the of proximal segment of OM1	100% occlusion under the opening	4	2.25*24Resolute?2.5*15?2.75*21 Lu Pu?2.5*33mm Firebird	54	37.5	57.75	82.5	231.75
a277	Normal ECG	95% stenosis in the proximal stenosis with calcification	Mild stenosis with tumor-like expansion	60% stenosis in the proximal and distal segments	1	3.5*33mm Firebird	115.5				115.5
a278	ST:I?II?III?aVF?V4-V6 horizontal depression 0.5-1.5mm, inverted T wave, left ventricular hypertrophy	95% stenosis in the distal segment	Irregular vessel wall	Irregular vessel wall	1	2.25*18Resolute	40.5				40.5
a279	T?I?II?a???V2-6 dual phase, Q-T extension	70% stenosis in the proximal segment, 70% stenosis in the D1, mild intimal hyperplasia	No obvious stenosis	Irregular vessel wall	1	3.5*28mmEXCEL balloon stent	98				98
a280	V1-V3 ?QS?ST?V2-V3 rises 0.5mm, T: I, V2-V6 upside down	100% occlusion in the proximal segment	70-80% stenosis in proximal segment	99% stenosis in the proximal segment and 90% stenosis in the distal segment	1	3.5*14mmEXCEL balloon stent	49				49

a281	ST-T changes, QT prolongs	60% stenosis in the stent	Irregular vessel wall	90% of the most severe stenosis in the proximal and middle segments	2	3.5*25mm TIVOLI	175				175
a282	Ventricular premature beats	Opening lesions with calcification, 90% stenosis	No obvious stenosis	80% stenosis in distal segment	1	3.5*14mmEXCEL balloon stent	49				49
a283	Atrial fibrillation	50% stenosis in the proximal mid-segment	Irregular vessel wall	Irregular vessel wall	1	3.5*18mmEXCEL balloon stent	63				63
a284	Extremely clockwise transposition, T: aVL biphasic	80-90% stenosis in the proximal mid-segment	Subtotal occlusion in the proximal and middle segments	95% stenosis in the proximal segment	2	3.0*36mmEXCEL ?3.5*30mm TIVOLI	108	105			213
a285	Normal ECG	No obvious stenosis	No obvious stenosis	90% stenosis in the distal segment	2	3.0*25?2.5*30mm TIVOLI	75	75			150
a286	ST: V3-6 horizontal depression 0.5-1mm, T: III, aVF inverted, II biphasic	85% stenosis in the proximal mid-segment	70% stenosis in the proximal segment and 75% in the distal segment	95% stenosis in the proximal segment and almost completely occluded in the distal segment	2	2.25*33mm Mei Li Dun stent?3.0*38 Ya Pei stent	74.25	114			188.25
a287	CRBBB,III?aVF is Qr	40% stenosis in the proximal segment, middle segment is myocardial bridge	No obvious stenosis	80% stenosis in the middle and distal segments	1	3.5*21mm TIVOLI		73.5			73.5
a288	Normal ECG	No obvious stenosis	No obvious stenosis	80% stenosis in the middle and distal segments	1	3.5*36mmEXCEL balloon stent		126			126

a289	ST:III?aVF horizontal depression 0.5mm	90% stenosis in the middle segment	No obvious stenosis	30-40% stenosis in the proximal and middle segments	1	2.5*15mmLEPU stent	37.5				37.5
a290	Normal ECG	95% stenosis in the middle segment, 80% stenosis in the D2 opening	40% stenosis in the middle segment	Irregular vessel wall	1	2.75*30Resolute	82.5				82.5
a291	Sinus rhythm	50-60% stenosis in the proximal mid-segment	75% stenosis in the middle segment	50% stenosis in the proximal segment, 99% in the middle segment, and 90% in the distal segment	3	2.25*24Resolute?3.0*25?2.5*30 mm TIVOLI	54	75	75		204
a292	T wave change	99% stenosis in the distal segment with calcification	50% stenosis in the proximal segment, 99% stenosis with calcification in the mid-distal segments	50-60% of stenosis in the proximal segment with calcification, complete occlusion in the distal segment	2	3.75*30mm TIVOLI?3.5*18mmEXCEL balloon stent	112.5	63			175.5
a293	Limb lead low voltage, ST-T changes	Subtotal occlusion in the proximal mid-segment	70% stenosis in the proximal segment	99% stenosis in the middle segment	1	3.0*15mm TIVOLI	45				45
a294	Dual-Chamber Pacing rhythm	The original stent is unobstructed, 90% stenosis in D1	50-60% stenosis in the distal segment	50% stenosis of the intimal hyperplasia of the proximal stent	3	3.75*18mmEXCEL balloon stent	67.5				67.5

a295	Normal ECG	The original stent has 85% stenosis in the proximal segment and 60% in the distal segment	Irregular vessel wall	40-50% stenosis in the proximal and middle segments	1						
a296		80% stenosis in the proximal segment	Irregular vessel wall	No obvious stenosis	1	3.5*13mm Firebird	45.5				45.5
a297	Left ventricular high voltage, ST-T changes	Occlusion in the proximal mid-segment	No obvious stenosis	99% stenosis in the proximal segment	1	2.75*36mmEXCEL balloon stent	99				99
a298	Sinus bradycardia	40-50% stenosis in the proximal mid-segment, 99% stenosis at D1 opening	No obvious stenosis	90% stenosis in the proximal and middle segments	1	2.75*33mmEXCEL balloon stent	90.75				90.75
a299	AVB, CRBBB, T: III, aVF inverted, II, V6 biphasic	85% stenosis in the proximal segment, intimal hyperplasia of the original stent	50% stenosis of the original stent in the proximal and middle segments	Intimal hyperplasia in the original stent	6	2.75*18?2.5*24 mmEXCEL balloon stent	742.5	60			802.5
a300	V1-V3 shows QS type, III, aVF shows qr, ST-T change	The original stent is unobstructed, 40% stenosis in D1	75% stenosis in the middle segment	85% stenosis at the junction of the middle and distal stents	3	3.0*12mmNANO	108				108

a301	I degree AVB	60-70% stenosis in the proximal stent, and the intima of the original stent in the middle segment is slightly hyperplastic	50% stenosis and length lesions in the proximal middle segments	85% stenosis with calcification in the proximal segment, 90% stenosis with calcification in the middle segment	5	2.75*18mm Firebird?3.0*18 mmEXCEL	198	54			252
a302	II?III?aVF shows QRs, ST-T changes	80% stenosis between the proximal and distal stents	99% stenosis in the opening	The original stent in the proximal and middle segments is unobstructed, the PDA is occluded, and the proximal PLA segment is 70% stenosis	4	3.5*18mmEXCEL	63				63
a303	Atrial premature beats	50-60% stenosis in the proximal mid-segment with calcification	No obvious stenosis	85% stenosis in the proximal segment	1	4.0*10mm xience	40				40
a304	CRBBB?T wave change	90% stenosis in the proximal segment	No obvious stenosis	30% stenosis in the middle segment	1	3.5*28XINSORB	98				98
a305	ST: V5-V6 horizontal depression 0.5mm	99% stenosis in the middle and distal segment	70% stenosis in the proximal and middle segments, 70% stenosis under the opening of OM	Long lesions, 80-90% stenosis	2	2.75*23?2.0*18 mmFirebird	63.25	36			99.25

a306	ST: I, II, V4-V6 horizontal depression 0.5mm, T: I, II, aVL two-phase, V4 low level	Long lesions in the proximal mid-segment with plaque rupture, more than 85% stenosis in the most stenosis	Irregular vessel wall	50% stenosis in the proximal and middle segment of PD	1	3.5*30Resolutue	105				105
a307	Sinus rhythm,Premature ventricular contractionsy, low voltage lead	80% stenosis in the proximal mid-segment with diffuse calcification	75% stenosis in the proximal and middle segments, and 95% stenosis in the distal segment	Completely occulated from the middle segment	4	3.0*36?3.0*33?2.75*36mmEXCEL?2.25*23mm XIENCE	108	99	99	51.75	357.75
a308	Sinus Rhythm, Premature ventricular contractionsy, I degree AVB, limb lead low voltage	40% stenosis in the proximal segment	90% stenosis in the distal segment of the original stent	80% stenosis in the middle segment	2	3.0*12 Le Pu stent	72				72
a309	Normal ECG	85% stenosis	The original stent was not obstructed, and 50% stenosis in the OM	The original stent is unobstructed, 90% stenosis in the proximal part of the stent, and 70% stenosis in the distal segment	3	2.5*18Resolutue	135				135
a310	Normal ECG	85% stenosis in the original stent	Unobstructed original stent	Irregular vessel wall	2						
