# Supplementary material for: Co-release of cytokines after drug-eluting stent implantation in acute myocardial infarction patients with PCI
Source: Sci Rep. 2024 Jan 12;14:1236. doi: 10.1038/s41598-024-51496-8 (PMC10786845; doi:10.1038/s41598-024-51496-8)
Supplement: Supplementary file 1 — Supplementary Information. [file 41598_2024_51496_MOESM1_ESM.zip › PCI-Suppltable 3.pdf]

Supplementary Table 3: PCI patients with additional features of diagnosis, measurements and treatment outcome

Name	Aspirin(mg)	Antiplatelet	beta-receptor antagonist(mg)	ACEI/ARB	Statins(mg)	Antidiabetic Medication	
a1	100	Clopidogrel	Metoprolol 23.75	Valsartan 80	Rosuvastatin 10		
a2	100	Brilinta		Olmesartan 20	Rosuvastatin 10		
a3	100	Brilinta	Metoprolol 23.75	Perindopril 4	Atorvastatin 20		
a4	100	Clopidogrel	Arotinolol 5	Olmesartan	Rosuvastatin 10	Metformin, Piaglitazone	
a5	100	Clopidogrel	Metoprolol 11.87	Benazepril 2.5	Simvastatin 20		
a6	100	Clopidogrel		Olmesartan	Rosuvastatin 10		
a7	100	Brilinta			Rosuvastatin 10		
a8	100	Brilinta	Metoprolol 23.75	Valsartan 80	Simvastatin 20		
a9	Cilostazol	Clopidogrel		Benazepril 5	Simvastatin 10		
a10	100	Brilinta	Metoprolol 23.75	Losartan 50	Rosuvastatin 10		
a11	100	Clopidogrel		Benazepril 5	Rosuvastatin 10	Rosiglitazone	
a12	Cilostazol	Clopidogrel			Simvastatin 20		
a13	100	Clopidogrel	Metoprolol 23.75		Simvastatin 20	No Medication	
a14	100	Clopidogrel	Metoprolol 47.5	Benazepril 5	Rosuvastatin 10		
a15	Cilostazol	Clopidogrel	Metoprolol 11.87	Benazepril 5	Simvastatin 20		
a16	100	Clopidogrel	Metoprolol 47.5	Benazepril 5	Simvastatin 20		
a17	100	Clopidogrel	Metoprolol 23.75		Simvastatin 20		
a18	100	Clopidogrel	Metoprolol 23.75		Simvastatin 20		
a19	100	Clopidogrel		Benazepril 5	Simvastatin 20		
a20	100	Brilinta	Metoprolol 6.25	Losartan 50	Rosuvastatin 10		
a21	100	Brilinta		Valsartan 80	Rosuvastatin 10		
a22	Cilostazol	Brilinta	Metoprolol 23.75		Rosuvastatin 10	INS	
a23	100	Brilinta	Metoprolol 23.75	Valsartan 80	Rosuvastatin 10		
a24		Clopidogrel	Metoprolol 23.75	Benazepril 5	Simvastatin 20	INS	
a25	100	Clopidogrel	Metoprolol 11.87	Benazepril 5	Simvastatin 20		
a26	100		Metoprolol 23.75		Atorvastatin 20	INS	
a27		Clopidogrel	Metoprolol 11.87		Simvastatin 20	INS	
a28	100	Clopidogrel	Metoprolol 23.75	Benazepril 5	Simvastatin 20		
a29	Cilostazol	Clopidogrel		Benazepril 5	Simvastatin 20		
a30	Cilostazol	Brilinta	Metoprolol 23.75	Irbesartan 150	Rosuvastatin 10		
a31	Cilostazol	Brilinta	Metoprolol 23.75	Valsartan 80	Atorvastatin 20		

a32	100	Brilinta	Metoprolol 23.75	Valsartan 80	Rosuvastatin 10		
a33	100	Brilinta	Metoprolol 23.75	Valsartan 80	Rosuvastatin 10		
a34	100	Brilinta	Metoprolol 23.75		Rosuvastatin 10		
a35	100	Brilinta	Metoprolol 71.25	Valsartan 80	Atorvastatin 20	INS	
a36	100	Brilinta	Metoprolol 23.75		Rosuvastatin 10		
a37		Brilinta			Rosuvastatin 10		
a38	100	Brilinta	Metoprolol 23.75	Valsartan 80	Rosuvastatin 10		
a39	100	Brilinta		Valsartan 80	Atorvastatin 20		
a40	100	Brilinta	Bisoprolol 2.5	Valsartan 80	Rosuvastatin 10		
a41	100	Brilinta			Rosuvastatin 10		
a42	100	Brilinta			Rosuvastatin 10		
a43	100	Brilinta	Metoprolol 47.5	Valsartan 80	Atorvastatin 20		
a44	100	Brilinta	Metoprolol 23.75		Rosuvastatin 10		
a45	100	Brilinta			Simvastatin 10		
a46	100	Brilinta		Valsartan 80	Rosuvastatin 10	INS	
a47	100	Brilinta	Metoprolol 23.75	Valsartan and Hy	Rosuvastatin 10		
a48	100	Clopidogrel	Metoprolol 23.75	Valsartan 80	Rosuvastatin 10		
a49	Cilostazol	Brilinta	Metoprolol 23.75	Valsartan 80	Rosuvastatin 10		
a50	100	Brilinta	Metoprolol 23.75		Atorvastatin 10		
a51	100	Brilinta	Bisoprolol 2.5	Valsartan and Hy	Rosuvastatin 10	INS	
a52	Cilostazol	Clopidogrel	Metoprolol 23.75		Rosuvastatin 10		
a53	100	Brilinta	Metoprolol 23.75	Olmesartan 20	Rosuvastatin 10	INS	
a54	100	Brilinta	Metoprolol 23.75	Valsartan 80	Rosuvastatin 10	Metformin, Glipizide	
a55	100	Brilinta		Valsartan and Hy	Simvastatin 10		
a56	100	Brilinta	Metoprolol 23.75	Valsartan and Hy	Rosuvastatin 10		
a57	100	Brilinta	Metoprolol 23.75	Olmesartan 20	Rosuvastatin 10		
a58	100	Brilinta	Metoprolol 23.75	Valsartan 80	Rosuvastatin 10		
a59		Brilinta	Metoprolol 23.75		Rosuvastatin 10		
a60	Cilostazol	Brilinta	Metoprolol 23.75		Rosuvastatin 10		
a61	100	Brilinta		Olmesartan 20	Atorvastatin 20	INS	
a62	100	Clopidogrel	Metoprolol 23.75		Rosuvastatin 10		
a63		Brilinta	Metoprolol 23.75		Rosuvastatin 10		
a64	100	Brilinta	Metoprolol 11.78	Valsartan and Hy	Atorvastatin 20		
a65	100	Brilinta	Metoprolol 23.75	Benazepril 10	Rosuvastatin 10	INS	

a66		Brilinta	Metoprolol 23.75		Atorvastatin 20		
a67	100	Brilinta	Metoprolol 23.75		Atorvastatin 20		
a68	100	Brilinta	Metoprolol 23.75		Rosuvastatin 10		
a69	100	Brilinta		Olmesartan 10	Atorvastatin 20	Metformin, Glipizide	
a70	100	Brilinta	Metoprolol 23.75		Atorvastatin 20	Metformin, Piaglitazone	
a71		Brilinta	Metoprolol 47.5	Valsartan 80	Rosuvastatin 10		
a72	100	Clopidogrel	Metoprolol 11.87	Benazepril 2.5	Rosuvastatin 10		
a73	100	Brilinta			Pravastatin 40	Rosiglitazone	
a74		Brilinta	Metoprolol 23.75		Rosuvastatin 10		
a75		Brilinta	Metoprolol 23.75		Rosuvastatin 10		
a76	100	Brilinta			Atorvastatin 20		
a77		Brilinta	Metoprolol 47.5		Atorvastatin 20	INS	
a78		Brilinta	Metoprolol 47.5		Rosuvastatin 10	INS	
a79	100	Brilinta		Valsartan 80	Rosuvastatin 10		
a80	100	Brilinta	Metoprolol 23.75		Atorvastatin 20		
a81		Brilinta					
a82	100	Brilinta		Losartan 50	Atorvastatin 20		
a83	Cilostazol	Brilinta	Metoprolol 23.75		Rosuvastatin 10		
a84	100	Brilinta	Metoprolol 23.75		Atorvastatin 20	INS	
a85	100	Brilinta	Metoprolol 23.75		Atorvastatin 20	INS	
a86	Cilostazol	Brilinta	Metoprolol 23.75				
a87	100	Brilinta	Metoprolol 47.5	Valsartan and Hy	Atorvastatin 20	Metformin, piaglitazone	
a88		Brilinta	Metoprolol 23.75		Rosuvastatin 10		
a89		Brilinta	Metoprolol 23.75	Olmesartan 20	Rosuvastatin 10		
a90	Cilostazol	Brilinta	Metoprolol 47.5		Rosuvastatin 10		
a91	100	Brilinta	Arotinolol 5		Rosuvastatin 10		
a92	100	Brilinta	Metoprolol 23.75	Valsartan 80	Atorvastatin 20		
a93	100	Brilinta	Metoprolol 23.75	Valsartan 80	Rosuvastatin 10	INS	
a94	100	Brilinta	Metoprolol 47.5	Fosinopril 5		No Medication	
a95	100	Brilinta	Metoprolol 23.75		Rosuvastatin 10	ins	
a96	100	Brilinta	Metoprolol 23.75		Rosuvastatin 10		
a97	100	Brilinta	Metoprolol 23.75	Losartan 50	Atorvastatin 20		
a98	100	Brilinta	Metoprolol 23.75	Valsartan 80	Rosuvastatin 10		
a99		Brilinta		Fosinopril 5	Atorvastatin 20		

a100	100	Brilinta	Metoprolol 23.75	Telmisartan 40	Rosuvastatin 10		
a101	100	Brilinta	Metoprolol 23.75	Valsartan 80	Atorvastatin 20		
a102		Brilinta	Metoprolol 47.5 BID		Rosuvastatin 10	No Medication	
a103	Cilostazol	Brilinta	Metoprolol 23.75	Losartan 50	Rosuvastatin 10		
a104	75	Brilinta	Metoprolol 23.75		Atorvastatin 20		
a105		Brilinta	Metoprolol 23.75	Candesartan 4	Atorvastatin 20		
a106	100	Brilinta	Metoprolol 23.75		Rosuvastatin 10		
a107	100	Brilinta	Metoprolol 23.75		Rosuvastatin 10		
a108	100	Brilinta	Metoprolol 23.75		Rosuvastatin 10		
a109	100	Brilinta	Metoprolol 23.75		Rosuvastatin 10	INS	
a110	100	Brilinta	Metoprolol 23.75		Rosuvastatin 10		
a111	100	Brilinta	Metoprolol 23.75		Atorvastatin 20	INS	
a112		Brilinta	Metoprolol 23.75		Rosuvastatin 10	INS	
a113	100	Brilinta	Metoprolol 23.75	Olmesartan 20	Atorvastatin 20		
a114	100	Brilinta	Metoprolol 23.75		Rosuvastatin 10		
a115	100	Brilinta		Telmisartan 80	Atorvastatin 20		
a116	100	Brilinta	Metoprolol 11.78	Losartan 100	Atorvastatin 20		
a117	100	Brilinta	Metoprolol 23.75		Atorvastatin 20		
a118		Brilinta	Metoprolol 23.75	Valsartan and Hy	Rosuvastatin 10		
a119	100	Brilinta	Metoprolol 23.75		Atorvastatin 20		
a120	100	Brilinta	Metoprolol 23.75		Rosuvastatin 10	INS	
a121	100	Brilinta	Metoprolol 47.5	Olmesartan 20	Rosuvastatin 5	INS	
a122	100	Brilinta		Losartan100	Atorvastatin 20		
a123	100	Brilinta	Metoprolol 23.75	Valsartan 80	Rosuvastatin 10	INS	
a124	Cilostazol	Brilinta	Metoprolol 11.78		Rosuvastatin 10		
a125	100	Brilinta	Metoprolol 23.75	Losartan 50	Rosuvastatin 10		
a126	100	Brilinta	Metoprolol 11.78	Valsartan 80	Rosuvastatin 10		
a127	100	Brilinta	Metoprolol 23.75	Valsartan 80	Rosuvastatin 10	INS	
a128	100	Brilinta	Metoprolol 23.75		Rosuvastatin 10		
a129	100	Brilinta	Metoprolol 23.75		Rosuvastatin 10	INS	
a130	100	Brilinta	Metoprolol 23.75	Valsartan 80	Rosuvastatin 10		
a131	100	Brilinta	Metoprolol 23.75		Rosuvastatin 10		
a132	100	Brilinta		Losartan100	Atorvastatin 20		
a133	100	Brilinta	Metoprolol 23.75		Atorvastatin 20	INS	

a134	100	Brilinta	Metoprolol 47.5	Losartan100	Rosuvastatin 10		
a135	100	Brilinta	Metoprolol 23.75		Rosuvastatin 10		
a136	100	Brilinta	Metoprolol 23.75	Valsartan 80	Rosuvastatin 10	INS	
a137	100	Brilinta	Metoprolol 47.5		Atorvastatin 20	Metformin, Rosiglitazone	
a138	Cilostazol	Clopidogrel		Benazepril 5	Simvastatin 20	Metformin, piaglitazone	
a139	100	Brilinta	Metoprolol 23.75		Atorvastatin 20		
a140	100	Brilinta	Metoprolol 23.75		Rosuvastatin 10	INS	
a141	100	Brilinta	Metoprolol 47.5		Atorvastatin 20	INS	
a142	100	Brilinta	Metoprolol 23.75		Atorvastatin 20		
a143	100	Brilinta	Metoprolol 23.75	Valsartan 80	Atorvastatin 20		
a144	100	Brilinta	Metoprolol 23.75		Rosuvastatin 10		
a145	100	Brilinta	Metoprolol 23.75	Valsartan 80	Rosuvastatin 10		
a146	100	Brilinta	Metoprolol 23.75		Rosuvastatin 10		
a147	100	Brilinta	Metoprolol 23.75	Valsartan 80	Rosuvastatin 10		
a148	100	Brilinta	Metoprolol 23.75	Valsartan 80	Atorvastatin 20		
a149	100	Brilinta	Metoprolol 47.5		Atorvastatin 20	INS	
a150	100	Brilinta	Metoprolol 23.75	Valsartan 80		INS	
a151	100	Brilinta	Bisoprolol 2.5		Atorvastatin 20	INS	
a152	100	Brilinta	Metoprolol 23.75		Rosuvastatin 10	INS	
a153	75	Clopidogrel	Metoprolol 23.75		Rosuvastatin 10		
a154	100	Clopidogrel	Metoprolol 47.5	Losartan 100	Rosuvastatin 10		
a155	100	Brilinta	Metoprolol 23.75		Rosuvastatin 10		
a156	100	Brilinta	Metoprolol 23.75		Rosuvastatin 10		
a157	100	Brilinta	Metoprolol 23.75	Valsartan 80	Atorvastatin 20		
a158	100	Brilinta	Metoprolol 23.75		Atorvastatin 20		
a159	100	Brilinta		Enalapril 20	Atorvastatin 20	INS	
a160	100	Brilinta	Metoprolol 23.75		Atorvastatin 20		
a161	100	Brilinta	Metoprolol 23.75		Rosuvastatin 10	INS	
a162	100	Brilinta	Metoprolol 47.5		Atorvastatin 20		
a163	100	Brilinta	Metoprolol 23.75		Simvastatin 10	INS	
a164	100	Brilinta	Bisoprolol 5	Fosinopril 10	Pravastatin 10		
a165	100	Brilinta	Metoprolol 47.5		Rosuvastatin 10	Metformin, Piaglitazone	
a166	100	Brilinta	Metoprolol 23.75	Telmisartan 40	Rosuvastatin 10		
a167	100	Brilinta	Metoprolol 23.75	Telmisartan 81	Rosuvastatin 10		

a168	100	Brilinta	Metoprolol 23.75		Rosuvastatin 10	Metformin, piaglitazone		
a169	100	Brilinta	Bisoprolol 5	Losartan 100	Simvastatin 20			
a170	100	Brilinta	Metoprolol 23.75		Rosuvastatin 10			
a171	100	Brilinta	Bisoprolol5	Valsartan 40	Rosuvastatin 10			
a172	Cilostazol	Clopidogrel	Metoprolol 23.75		Rosuvastatin 10			
a173	100	Brilinta	Metoprolol 23.75		Rosuvastatin 10			
a174	100	Brilinta	Metoprolol 23.75		Rosuvastatin 10			
a175	100	Brilinta	Metoprolol 23.75		Rosuvastatin 10			
a176	100	Brilinta	Metoprolol 23.75		Rosuvastatin 10			
a177	100	Brilinta	Metoprolol 23.75	Valsartan 80	Rosuvastatin 10			
a178		Brilinta	Bisoprolol 2.5	Losartan 100	Atorvastatin 20			
a179	100	Brilinta	Metoprolol 23.75	Valsartan 80	Rosuvastatin 10			
a180	100	Brilinta	Bisoprolol 5	Fosinopril 10	Pravastatin 10			
a181	100	Brilinta	Metoprolol 23.75					
a182		Brilinta	Metoprolol 23.75		Atorvastatin 20			
a183	100	Brilinta	Metoprolol 23.75	Telmisartan 80	Rosuvastatin 10			
a184	Cilostazol	Brilinta			Rosuvastatin 10			
a185	Cilostazol	Brilinta		Telmisartan 80	Rosuvastatin 10	INS		
a186	100	Brilinta	Metoprolol 23.75	Losartan 50	Atorvastatin 20			
a187	Cilostazol	Brilinta	Metoprolol 23.75	Valsartan and Hy	Rosuvastatin 10			
a188	100	Brilinta	Bisoprolol 2.5	Benazepril10				
a189	Cilostazol	Brilinta	Metoprolol 23.75		Rosuvastatin 10			
a190	100	Brilinta	Metoprolol 23.75	Valsartan and Hy	Atorvastatin 20			
a191	100	Brilinta	Metoprolol 23.75		Rosuvastatin 10			
a192	100	Brilinta	Metoprolol 23.75	Fosinopril 10	Simvastatin 20			
a193	100	Brilinta	Metoprolol 23.75		Rosuvastatin 10			
a194	100	Brilinta	Metoprolol 23.75	Losartan 100	Rosuvastatin 10			
a195	100	Brilinta	Metoprolol 47.5	Losartan 100	Atorvastatin 20	INS		
a196	100	Brilinta	Metoprolol 23.75	Valsartan and Hy	Atorvastatin 20			
a197	100	Brilinta			Simvastatin 20			
a198	100	Brilinta	Metoprolol 23.75		Atorvastatin 20			
a199	100	Brilinta	Metoprolol 23.75		Rosuvastatin 10			
a200	100	Brilinta	Metoprolol 23.75	Olmesartan 20	Rosuvastatin 10			
a201	100	Brilinta		Valsartan and Hy	Rosuvastatin 10			

a202	100	Brilinta	Metoprolol 23.75		Atorvastatin 20		
a203	100	Brilinta	Metoprolol 23.75	Telmisartan40	Rosuvastatin 10		
a204	100	Brilinta	Metoprolol 23.75		Rosuvastatin 10		
a205	100	Brilinta	Bisoprolol 2.5	Losartan 25	Rosuvastatin 10		
a206	100	Clopidogrel	Metoprolol 23.75	Losartan 50	Rosuvastatin 10	INS	
a207	100	Brilinta	Metoprolol 23.75	Losartan 100	Rosuvastatin 10		
a208	100	Brilinta	Metoprolol 23.75		Rosuvastatin 10		
a209	100	Brilinta	Metoprolol 47.5	Valsartan and Hy	Rosuvastatin 10	Metformin, piaglitazone	
a210	100	Brilinta	Metoprolol 47.5		Rosuvastatin 10		
a211	100	Brilinta	Metoprolol 23.75		Rosuvastatin 10		
a212	100	Brilinta	Metoprolol 47.5	Valsartan and Hy	Rosuvastatin 10		
a213		Brilinta	Metoprolol 23.75		Rosuvastatin 10		
a214	Cilostazol	Brilinta	Metoprolol 23.75	Valsartan and Hy	Rosuvastatin 10		
a215	100	Clopidogrel	Metoprolol 47.5		Atorvastatin 20	Rosiglitazone	
a216	100	Brilinta	Bisoprolol 2.5	Losartan 50	Atorvastatin 20	Metformin, Piaglitazone	
a217	100	Brilinta	Metoprolol 23.75	Olmesartan 20	Rosuvastatin 10		
a218	100	Brilinta	Metoprolol 23.75		Rosuvastatin 10		
a219	100	Brilinta	Metoprolol 11.87		Simvastatin 20	Metformin, Piaglitazone	
a220	100	Brilinta	Metoprolol 11.87		Atorvastatin 20		
a221	75	Brilinta	Metoprolol 23.75		Atorvastatin 20		
a222	100	Brilinta	Metoprolol 23.75		Rosuvastatin 10		
a223	100	Brilinta	Metoprolol 47.5	Telmisartan 40	Rosuvastatin 10	Metformin, Piaglitazone	
a224	100	Brilinta			Rosuvastatin 10		
a225	100	Brilinta	Metoprolol 23.75	Valsartan and Hy	Atorvastatin 20		
a226	100	Brilinta	Metoprolol 23.75		Rosuvastatin 10	INS	
a227	100	Brilinta				INS	
a228	100	Brilinta	Metoprolol 23.75		Rosuvastatin 10		
a229	100	Brilinta	Metoprolol 23.75	Benazepril 10	Atorvastatin 20	INS	
a230	100	Brilinta	Metoprolol 23.75	Losartan 50	Rosuvastatin 10	INS	
a231	100	Brilinta	Metoprolol 23.75	Valsartan 80	Atorvastatin 20		
a232	100	Brilinta	Metoprolol 23.75		Rosuvastatin 10		
a233	100	Brilinta	Metoprolol 23.75	Valsartan 80	Atorvastatin 20		
a234	100	Brilinta	Metoprolol 23.75		Atorvastatin 20	No Medication	
a235	100	Brilinta	Metoprolol 23.75	Irbesartan Hydrochlorothiazide 150			

a236	75	Brilinta	Metoprolol 23.75		Rosuvastatin 10			
a237		Brilinta	Metoprolol 23.75					
a238	75	Brilinta	Metoprolol 23.75					
a239	Cilostazol	Brilinta	Metoprolol 23.75		Rosuvastatin 10			
a240	100	Brilinta	Metoprolol 23.75	Olmesartan 20	Atorvastatin 20			
a241	100	Brilinta	Metoprolol 47.5	Perindopril 4	Rosuvastatin 10			
a242	100	Brilinta		Olmesartan 20	Rosuvastatin 10	INS		
a243		Brilinta	Metoprolol 23.75	Losartan 100	Rosuvastatin 10			
a244	100	Brilinta	Metoprolol 23.75	Losartan 50	Rosuvastatin 10	Metformin, Glipizide		
a245	100	Brilinta	Metoprolol 23.75	Losartan Hydrocl	Rosuvastatin 10	Metformin, piaglitazone		
a246	100	Brilinta	Metoprolol 23.75	Olmesartan 20	Atorvastatin 20			
a247	100	Brilinta	Bisoprolol 2.5	Olmesartan 20	Atorvastatin 20			
a248		Clopidogrel	Metoprolol 23.75		Rosuvastatin 10			
a249	100	Brilinta	Metoprolol 23.75	Valsartan 80	Rosuvastatin 10			
a250	100	Brilinta	Metoprolol 23.75		Rosuvastatin 10			
a251	100	Brilinta	????11.87	Valsartan 80	Atorvastatin 20			
a252	Cilostazol	Clopidogrel	Metoprolol 23.75	Valsartan 80	Rosuvastatin 10			
a253		Brilinta	Metoprolol 23.75	Valsartan 80	Rosuvastatin 10			
a254	100	Brilinta	Metoprolol 23.75		Atorvastatin 20			
a255	100	Clopidogrel	Metoprolol 23.75	Valsartan 80	Rosuvastatin 10			
a256	100	Brilinta	Metoprolol 23.75		Rosuvastatin 10	INS		
a257	Cilostazol	Clopidogrel	Bisoprolol 5	Benazepril 10	Rosuvastatin 10	INS		
a258	100	Brilinta	Arotinolol 20	Valsartan 80	Rosuvastatin 10			
a259	100	Clopidogrel	Metoprolol 23.75	Losartan 100	Atorvastatin 20			
a260	100	Brilinta	Metoprolol 23.75	Fosinopril 10	Atorvastatin 20	INS		
a261	100	Brilinta	Metoprolol 47.5		Rosuvastatin 10			
a262	100	Brilinta	Metoprolol 23.75		Rosuvastatin 10			
a263	100	Brilinta	Metoprolol 23.75	Valsartan 80	Rosuvastatin 10			
a264	100	Brilinta	Metoprolol 23.75	Benazepril 10	Rosuvastatin 10			
a265	100	Brilinta	Metoprolol 23.75		Rosuvastatin 10	Metformin, Piaglitazone		
a266	100	Brilinta	Metoprolol 23.75		Rosuvastatin 10			
a267	100	Brilinta	Metoprolol 47.5	Losartan Potassiu	Rosuvastatin 10			
a268	100	Clopidogrel	Metoprolol 23.75	Valsartan 80	Rosuvastatin 10	INS		
a269	Cilostazol	Clopidogrel	Metoprolol 47.5	Valsartan 80	Rosuvastatin 10	Rosiglitazone		

a270	100	Brilinta	Metoprolol 23.75	Valsartan 80	Rosuvastatin 10	Metformin, piaglitazone	
a271	100	Clopidogrel		Losartan 100	Atorvastatin 20		
a272	100	Clopidogrel	Metoprolol 23.75	Losartan Potassiu	Atorvastatin 20	Metformin, Rosiglitazone	
a273	100	Brilinta	Metoprolol 23.75	Losartan Potassiu	Rosuvastatin 10		
a274	Cilostazol	Clopidogrel	Metoprolol 23.75		Rosuvastatin 10		
a275	Cilostazol	Brilinta	Metoprolol 23.75	Valsartan 80	Atorvastatin 20		
a276	100	Clopidogrel	Metoprolol 23.75	Losartan 100	Atorvastatin 20	No Medication	
a277	100	Brilinta	Bisoprolol 2.5	Losartan 100	Rosuvastatin 10	INS	
a278	100	Clopidogrel	Metoprolol 23.75		Atorvastatin 20		
a279		Brilinta	Metoprolol 23.75		Rosuvastatin 10		
a280	100	Brilinta	Metoprolol 23.75	Benazepril 2.5	Rosuvastatin 10		
a281	100	Brilinta	Metoprolol 23.75	Valsartan 80	Atorvastatin 20		
a282	100	Brilinta	Metoprolol 23.75	Valsartan and Hy	Rosuvastatin 10		
a283	100	Brilinta	Metoprolol 23.75		Atorvastatin 20	INS	
a284	100	Brilinta	Metoprolol 23.75		Rosuvastatin 10		
a285	Cilostazol	Clopidogrel	Metoprolol 23.75		Rosuvastatin 10		
a286	100	Brilinta	Metoprolol 23.75		Atorvastatin 20	No Medication	
a287	100	Brilinta	Metoprolol 23.75	Valsartan 80	Rosuvastatin 10	Metformin, Piaglitazone	
a288	100	Brilinta	Metoprolol 23.75		Rosuvastatin 10		
a289	100	Brilinta	Metoprolol 23.75		Rosuvastatin 10		
a290	100	Brilinta	Metoprolol 23.75	Valsartan and Hy	Rosuvastatin 10		
a291	100	Clopidogrel	Metoprolol 23.75	Losartan Hydroch	Atorvastatin 20	Metformin, Rosiglitazone	
a292	100	Brilinta	Metoprolol 23.75	Benazepril 5	Atorvastatin 20	No Medication	
a293	75	Clopidogrel			Rosuvastatin 10		
a294	100	Clopidogrel	Metoprolol 23.75	Telmisartan 80	Rosuvastatin 10		
a295		Brilinta	Bisoprolol 2.5		Rosuvastatin 10		
a296	100	Brilinta	Metoprolol 23.75		Rosuvastatin 10	INS	
a297	100	Brilinta	Bisoprolol 2.5		Rosuvastatin 10		
a298	100	Brilinta	Bisoprolol 2.5	Losartan 100	Atorvastatin 20	INS	
a299	100	Brilinta		Benazepril 5	Rosuvastatin 10		
a300	100	Brilinta	Almarl 20mg		Rosuvastatin 10	No Medication	
a301	100	Clopidogrel	Metoprolol 23.75		Rosuvastatin 10		
a302	100	Brilinta	Almarl 20mg	Irbesartan Hydroc	Rosuvastatin 10		
a303		Brilinta	Metoprolol 23.75		Rosuvastatin 10		

a304	100	Brilinta	Metoprolol 23.75		Atorvastatin 20		
a305	100	Clopidogrel	Metoprolol 23.75	Losartan 100	Atorvastatin 20		
a306	100	Clopidogrel	Metoprolol 47.5	Irbesartan Hydroc	Atorvastatin 20		
a307	100	Brilinta	Metoprolol 23.75		Rosuvastatin 10		
a308	100	Brilinta	Metoprolol 23.75	Losartan 100	Atorvastatin 20		
a309	100	Brilinta	Metoprolol 23.75	Valsartan 80	Rosuvastatin 10	INS	
a310	100	Brilinta	Metoprolol 23.75	Valsartan 80	Rosuvastatin 10	Metformin, glipizide	
