# Supplementary material for: Co-release of cytokines after drug-eluting stent implantation in acute myocardial infarction patients with PCI
Source: Sci Rep. 2024 Jan 12;14:1236. doi: 10.1038/s41598-024-51496-8 (PMC10786845; doi:10.1038/s41598-024-51496-8)
Supplement: Supplementary file 1 — Supplementary Information. [file 41598_2024_51496_MOESM1_ESM.zip › PCI-Suppltable 5.pdf]

Supplementary Table 5: Correlation of cytokine release with ejection fraction (LVEF%)

Criteria	Cytokines	No of Sample	Number of stent (Correlation coefficient (r^2)	P-Value	No of Sample	Total length of Stent (Correlation Coefficient (r^2)	P-Value
LVEF%	Hs-CRP	97	-0.07, 95%CI (-0.26-0.13)	0.50	183	-0.14, 95% (-0.28-0.0035)	0.055
LVEF%	TNFα	107	-0.030, 95% CI (-0.21-0.16)	0.75	205	-0.05, 95% CI (-0.18-0.09))	0.51
LVEF%	IL10	51	-0.38, 95% CI (-0.28-0.21))	0.76	123	-0.35, 95% CI (-0.21-0.14)	0.70
LVEF%	IL6	99	-0.07, 95% CI (-0.27-0.12)	0.44	181	-0.09, 95% CI(-0.24-0.04)	0.18
LVEF%	IL2eceptor	107	-0.27, 95% CI (-0.44—0.09)	0.004	205	-0.23, 95%CI (-0.36—0.10)	0.0006
LVEF%	IL-1ß	62	-0.10, 95%CI (-0.34-0.14)	0.41	121	-0.90, 95% CI (-0.26-0.089)	0.32
LVEF%	IL8	107	-0.07, 95% CI (-0.26-0.11)	0.43	204	-0.07, 95% CI (-0.21-0.06)	0.28
