# Supplementary material for: Co-release of cytokines after drug-eluting stent implantation in acute myocardial infarction patients with PCI
Source: Sci Rep. 2024 Jan 12;14:1236. doi: 10.1038/s41598-024-51496-8 (PMC10786845; doi:10.1038/s41598-024-51496-8)
Supplement: Supplementary file 1 — Supplementary Information. [file 41598_2024_51496_MOESM1_ESM.zip › pci-suppletable 4.pdf]

Supplementary Table 4: Baseline characteristics of PCI patients					
**Criteria**	**symptoms/Cytokine 1**	**Cytokine 2**	**Sample number**	**Mean**	
Age	Diabetes		91	66.26	
Age	No Diabetes		219	67.34	
Age	Hypertension		207	68	
Age	No		103	65.25	
Age	TNFα		150	46.85	
Age	IL8		149	41.46	
Age	IL-ß		94	1.24	
STEMI	TNFα	IL8	23	42.039	
STEMI	TNFα	IL-1ß	13	0.55	
NSTEMI	TNFα	IL8	20	43.02	
NSTEMI	TNFα	IL-1ß	12	1.2	
UA	TNFα	IL8	123	50.7	
UA	TNFα	IL-1ß	60	3.36	
CAD	TNFα	IL8	48	53.37	
CAD	TNFα	IL-1ß	36	1.18	
LAD-PCI	TNFα	IL8	119	44.75	
LAD-PCI	TNFα	IL-1ß	67	1.88	
RCA-PCI	TNFα	IL8	82	42.77	
RCA-PCI	TNFα	IL-1ß	49	1.44	
LCX-PCI	TNFα	IL8	44	40.12	
LCX-PCI	TNFα	IL-1ß	23	1.16	
Diabetes	TNFα	IL-1ß	49	2.72	
No Diabetic	TNFα	IL-1ß	134	1.68	
Diabetes	TNFα	IL8	90	42.35	
No Diabetes	TNFα	IL8	218	50.28	
Hypertension	TNFα	IL-1B	125	1.8	
No Hypertension	TNFα	IL-1ß	58	2.1	
Hypertension	TNFα	IL8	206	48.98	
No Hypertension	TNFα	IL8	102	45.89	
Hypertension	No. of STENT		195	1.8	
No Hypertension	No of STENT		99	1.68	
Age	No of STENT		150	66.7	
Blood Glucose level	No of STENT		149	5.1	
RCA-PCI	No of STENT		79	2.3	
LAD-PCI	No of STENT		113	1.5	
No of STENT	HS-CRP		251	7.15	

No of STENT	HS- CRP <5mg/L		188	1.7	
No of STENT	HS- CRP >5mg/L		63	1.8	
No of STENT	HS-CRP	IL8	128	7.1	
No. of STENT	Hs-CRP	IL6	106	6.68	
No of STENT	HS-CRP	IL2-receptor	129	509.59	
No of STENT	HS-CRP	IL10	82	2.3	
No of STENT	HS-CRP	IL-1ß	78	1.5	
Total STENT Length	No of STENT		205	71.79	
Total STENT Length	RCA-PCI		80	134.4	
Total STENT Length	LAD-PCI		119	102.76	
Total STENT Length	LCX-PCI		46	76.8	
Total STENT Length	CAD		48	121.77	
Total STENT Length	UA		120	102.52	
Total STENT Length	TNFα		298	47.95	
Total STENT Length	IL-1ß		177	2.1	
Total STENT Length	IL8		296	44.25	
Total STENT Length	Hs-CRP		264	8.28	
Metoprolol 23.75	TNFα	IL8	204	63.14	
Metoprolol 11.87	TNFα	IL8	6	70.19	
Metoprolol 23.75	TNFα	IL1-ß	128	2.23	
Metoprolol 11.87	TNFα	IL1-ß	5	1.3	
Simvastatin-20	TNFα	IL8	19	45.14	
rosuvastatin-10	TNFα	IL8	185	48.61	
Simvastatin-20	TNFα	IL-1ß	8	0.66	
rosuvastatin-10	TNFα	IL-1ß	113	1.22	
benazepril-5.0	TNFα	IL8	13	48.58	
benazepril-2.5	TNFα	IL-1ß	5	15.6	
valsartan 80	TNFα	IL8	53	53.73	
olmesartan 20	TNFα	IL8	9	33.69	
clopidogrel	TNFα	IL8	48	46.64	
brilinta	TNFα	IL8	259	48.34	
clopidogrel	TNFα	IL-1ß	29	1.02	
brilinta	TNFα	IL-1ß	154	2.14	
