# Supplementary material for: Co-release of cytokines after drug-eluting stent implantation in acute myocardial infarction patients with PCI
Source: Sci Rep. 2024 Jan 12;14:1236. doi: 10.1038/s41598-024-51496-8 (PMC10786845; doi:10.1038/s41598-024-51496-8)
Supplement: Supplementary file 1 — Supplementary Information. [file 41598_2024_51496_MOESM1_ESM.zip › PCI-suppl table 1.pdf]

	Supplementary table 1: PCI Patients with diagnosis and cytokine levels																						
																*							
Admission da	Name	Gender	Age	Diagnosis	hypertension	Diabetes	HDL-C	LDL-C	WBC*10^9	PLT*10E9/L	Blood glucose (HbA1C)	TG	LVEF%	hs-CRPmg/l)	Hbhc1		IL2 Recep	IL6	IL8	IL10	TNF	Results of Coronary angiography	syntax Score
2014.01	a1	M	65	STEMI	Yes	No	1.18	1.39	6.8	194	4.4	2	59	1.98		0.7	417	2.16	18	0.64	13.8	LAD-PCI	34.5
2014.01	a2	M	61	ACS	No	Yes	1.22	1.91	8.5	163	6.05	1	65	0.83	6.5	<5	366	<2	24	<5	10.7	RCA-PCI/LCX-PCI	22
2014.01	a3	F	79	UA	No	Yes	1.28	2.47	6.3	152	3.75	1	64	1.35	10.1	<5	444	3.32	46	<5	97.3	LAD-PCI	23
2014.01	a4	F	80	STEMI	Yes	Yes	1.43	2.09	6.02	203	7.04	1	55	1.13	7.1	<5	544	9.7	100	<5	95.5	LCX-OM-PCI	27
2014.01	a5	M	65	STEMI	Yes	No	1.06	2.7	10.2	205	5.76	3	62.7	8.2	6.9	0.2	243	1.81	11	0.59	17.6	RCA-PCI	21
2014.02	a6	M	72	UA	No	Yes	1.53	2.69	6.3	145	4.71	1	57	1.04		<5	529	<2	18	<5	56	LAD-PCI	18
2014.02	a7	M	51	UA	No	Yes	1.58	2.68	6.8	200	5.62	1	55	2.05	6.5	<5	445	<2	12	<5	7.75	RCA-PCI	29
2014.02	a8	M	61	UA	Yes	No	1.22	2.34	6.9	151	4.97	1	70	1.77	5.6	0.3	563	2.15	4.5	1.17	11.6	RCA-PCI	19
2014.02	a9	F	75	STEMI	No	No	1.36	3.56	7.89	173	6.27	2	36.7	2.96	6.4	<5	340	4.83	10	<5	19.5	RCA-PCI/Stent recanalization	24
2014.02	a10	F	82	ACS	Yes	No	1.24	3.07	5.8	128	5.49	2	52	42.62	5.4	<5	838	5.66	65	50.3	72.2	LCX-PCI	22
2014.03	a11	M	70	STEMI	Yes	Yes	1.17	3.25	7.1	135	7.12	0	34.4	1.02	6.3	<5	745	3.17	8.8	<5	20	nmc	38
2014.03	a12	M	52	STEMI	No	No	1.2	3.59	9	169	5.68	1		0.89	6.3	<5	263	3.45	14	<5	33.3	LCX-PCI	8.5
2014.03	a13	F	66	NSTEM	No	Yes	1.36	4.11	5.84	228	12.13	3	37.1	0.83	7.7	<5	649	11.6	11	<5	7.05	RCA-PCI/LAD-PCI	25
2014.03	a14	M	66	STEMI	No	No	1.28	2.9	9.9	146	6.31	1	35.6	10.55	7.1	0.4	857	23	43	0.66	9.58	LAD-PCI	16
2014.03	a15	M	81	NSTEM	Yes	No	1.27	1.18	6.7	137	4.71	1		108.59	5.6	<5	612	7.04	294	<5	285	LCX-PCI	27
2014.03	a16	M	37	NSTEM	Yes	No	1.18	2.62	12.9	298	5.71	2	66.9	7.57		0.7	665	10.3	42	12.2	23.6	????	51.5
2014.03	a17	M	60	NSTEM	Yes	No	1.07	2.18	5.2	170	4.29	3	50.7	10.4		<5	1304	32.1	14	<5	28.5	RCA-PCI	36
2014.03	a18	M	52	NSTEM	Yes	No	1.17	3.29	5.8	323	6.57	2	58.1	110.26		<5	1171	3.73	19	<5	27	RCA-PCI	35.5
2014.03	a19	M	67	STEMI	Yes	No	1.32	3.78	11.7	232	5.63	1	63.6	1.32		<5	1000	7.24	138	<5	43.6	RCA-PCI	33.5
2014.03	a20	M	82	ACS	Yes	No	0.62	1.97	10.6	262	10.1	2	63.7	58		5.6	2529	54.2	113	5.61	16.2	LCX-PCI	10
2014.04	a21	M	51	After P	Yes	No	1.23	1.22	5.5	235	4.81	1	69.4	0.83	5.4	<5	339	3.78	31	<5	61.6	LAD-PCI	17
2014.04	a22	M	81	UA	No	Yes	1.14	1.64	4.1	186	6.05	2	62	2.65	6.5	<5	464	7.48	30	<5	19.8	LCX-PCI	31
2014.04	a23	F	55	UA	Yes	No	1.17	2.04	7.9	252	5.93	3	64.9	6.85	6	<5	446	2.38	35	<5	33.2	LAD-PCI	9

2014.04	a24	F	70	STEMI	No	Yes	1.23	1.88	5.4	172	3.44	2		3.4	10.8	<5	571	3.65	39	<5	41.3	RCA-PCI	20
2014.04	a25	M	80	NSTEM	No	No	1.05	2.75	7.8	157	4.35	3	63.3	<8		<5	415	17.8	8.2	<5	7.81	RCA-PCI	18
2014.04	a26	M	57	NSTEM	No	Yes	1.3	2.87	5.88	242	4.49	3	61	9.33	6.4	<5	340	<2	7.6	<5	9.41	LAD-PCI	16
2014.04	a27	F	75	STEMI	No	Yes	1.57	2.11	8	167	3.66	2	62	42.17	6.8	<5	937	10.2	71	<5	264	LCX-PCI	39
2014.04	a28	M	70	NSTEM	No	No	1.34	2.91	4.8	154	5.9	1	67.3	1.39		<5	552	4.81	53	<5	61.5	LCX-PCI	23
2014.04	a29	M	85	UA	Yes	No	1.35	2.39	8.8	124	5.3	0	70	17	5.6	1.2	453	1.35	6.1	1.22	25.1	RCA-PCI	52.5
2014.05	a30	M	81	CAD	Yes	No	1.65	2.42	6.66	229	4.45	1	61	6.46	5.7	<5	418	2.24	26	<5	49.2	RCA-PCI	39
2014.05	a31	M	72	UA	Yes	No	1.33	2.32	5.27	142	5.19	1	56	2.01	5.6	<5	442	3.52	77	<5	77.3	RCA-PCI	5
2014.05	a32	M	59	UA	Yes	No	1.33	2.59	7.7	237	4.69	1		3.01	5.7	<5	484	5.3	176	<5	85.8	LAD-PCI	12
2014.05	a33	M	66	UA	Yes	No	1	1.61	6.02	328	4.25	2	52	7.06	5.6	<5	859	12.6	148	10.2	142	RCA-PCI	17
2014.05	a34	M	71	UA	Yes	No	1.33	1.35	9	215	4.68	2	74	1.07	5.5	<5	380	4.88	294	<5	97.3	LCX-PCI	14
2014.05	a35	F	81	STEMI	Yes	Yes	1.48	14.4	3.9	173	3.98	2	63	6.9	9	1	571	3.7	17	1.08	10.3	LCX-PCI	37
2014.05	a36	M	74	UA	No	No	1.21	2.35	7.5	209	4.4	2	54	1.46	6.3	<5	444	4.51	159	<5	97.2	LCX-PCI	22
2014.05	a37	M	52	UA	No	No	1.12	2	8.2	130	3.48	2	71	0.83	5.5	<5	593	3.55	151	<5	173	LAD-PCI	10
2014.05	a38	M	58	UA	Yes	No	1.16	3.36	10.6	289	5.14	3	74	4.81	5.7	<5	763	3.64	15	<5	25.3	LAD-PCI	41
2014.05	a39	M	65	After P	No	No	1.65	3.55	5.43	135	6	4	64	1.35	5.7	<5	275	2.03	38	<5	42.6	LAD-PCI	10
2014.05	a40	M	55	ACS	No	No	1.37	1.97	4.9	171	3.59	2	68	1.64	6.1	<5	464	<2	86	<5	153	LAD-PCI	28
2014.05	a41	M	74	UA	No	No	1.61	3.46	4.76	98	5.62	3		0.83		<5	280	<2	28	<5	71.6	RCA-PCI	19
2014.05	a42	M	72	UA	No	No	1.18	1.33	8.1	232	2.79	1	66	2.51	5.6	<5	317	8.36	24	<5	10.6	LAD-PCI	27
2014.05	a43	M	52	STEMI	Yes	No	0.9	1.51	10.8	229	2.82	2	69	1.97	5.8	<5	379	4.83	267	<5	127	LAD-PCI	16
2014.05	a44	M	73	STEMI	Yes	No	1.66	1.33	10.2	282	4.01	1	56.8	2.25	5.9	<5	370	36.1	50	<5	20	LAD-PCI	20
2014.05	a45	M	73	After P	Yes	No	0.92	1.46	8.9	391	2.96	1	46	16		<5	518	2.38	13	<5	15.5	RCA-PCI	28
2014.05	a46	M	71	UA	No	Yes	1.47	1.57	6.5	138	3.18	2	73	1.04	7.8	<5	312	<2	48	<5	79.3	RCA-PCI	23
2014.06	a47	M	75	UA	Yes	No	1.99	2.58	6.4	195	4.49	1	69.4	0.83	5.1	<5	263	<2	58	<5	60.4	RCA-PCI	5
2014.06	a48	M	67	UA	Yes	No			6.1	120				<8		<5	522	10.9	485	<5	0.049	LAD-PCI	12
2014.06	a49	M	52	AM	No	No	1.27	1.85	9.39	150	5.18	2		1.72	6.5	<5	495	<2	77	<5	79.5	LCX-PCI	20
2014.06	a50	M	64	NSTEM	Yes	No	0.99	2.27	5.6	187	3.75	2	65	40.1	5.9	<5	319	8.08	37	<5	34	LAD-PCI	44.5
2014.06	a51	F	62	UA	Yes	Yes	1.41	3.26	8.8	265	5.25	3	71	6.7	7.7	<5	534	<2	36	<5	35.2	RCA-PCI	15
2014.06	a52	M	51	ACS	No	No	1.26	3.04	11.2	240	4.97	3	73	1.33	5.8	<5	488	2.44	25	<5	48.6	LAD-PCI	10
2014.06	a53	M	84	UA	Yes	Yes	1.71	1.78	8.6	128	3.73	2	68.4	1.41	11.8	<5	597	4.56	105	<5	45.6	LCX-PTCA	11

2014.06	a54	F	80	UA	Yes	Yes	1.19	5.08	6.9	192	7.94	5	76	3.76	13.6	<5	540	6.51	77	<5	81.9	LAD-PCI	29
2014.06	a55	M	55	After P	Yes	No	1.04	2.43	7.07	274	4.24	2		1.43	5.3	<5	389	2.88	31	<5	36.8	RCA-PCI	23
2014.06	a56	F	66	ACS	Yes	No	1.74	3.14	5.76	188	5.26	1		<8	6	<5	256	10.1	47	<5	35.2	LAD-PCI	8
2014.06	a57	F	77	UA	Yes	No	1.2	2.54	5.3	164	4.31	3		0.83	6.6	<5	970	5.35	198	<5	106	LCX-PCI	15
2014.06	a58	M	69	After P	Yes	No	1.07	2.42	6.6	150	3.94	1	50	0.83	5.5	<5	394	3.16	188	<5	156	LAD-PCI	26
2014.06	a59	M	54	AMI	Yes	No	1.38	3.58	5.58	166	5.56	4	65	2.36	6.3	<5	331	56.6	29	<5	25.6	LAD-PCI	47
2014.06	a60	M	70	UA	Yes	No	0.86	1.17	5.6	174	4.18	1		2.53	6	<5	193	4.01	43	<5	61	RCA-PCI	39
2014.06	a61	M	55	UA	Yes	Yes	1.34	3.29	8.9	172	5.12	3	63.9	10.15	8.4	<5	1700	10.8	81	<5	70.1	trigeminal-PCI.	48
2014.06	a62	M	57	UA	No	No	1.46	3.47	5.5	164	4.74	2	69	0.9	5.6	<5	322	7.25	103	<5	67.2	LCX-PCI	5
2014.06	a63	M	58	ACS	No	No	0.86	2.47	7.2	169	3.76	2	66	0.68	5.5	<5	399	2.17	30	<5	25.4	LM-LCX-PCI	15
2014.06	a64	F	83	UA	Yes	No	1.6	1.22	5.5	217	2.89	1	65	4.7	7	<5	1087	6.67	77	<5	178	RCA-PCI	38
2014.06	a65	M	38	After P	No	Yes	1.11	1.06	5	129	2.38	1	47.4	2.18	9.1	<5	340	2.29	51	<5	76.3	LCX-PCI	26
2014.06	a66	M	79	UA	Yes	No	1.57	1.53	6.8	151	3.14	2		1	6.1	<5	410	<2	83	<5	97.2	LCX-PCI	26
2014.06	a67	F	56	UA	No	No	1.72	2.24	10	290	4.05	2	73	2.08	5.7	<5	347	<2	27	<5	44	RCA-PCI	41.5
2014.06	a68	M	76	MI	Yes	No	1.02	0.87	5.1	96	1.82	1	54	1.02	7.3	5.7	400	7.34	397	<5	100	LAD-PCI	20
2014.06	a69	F	72	UA	Yes	Yes	1.3	2.99	11	193	10.8	2		0.3	6.8	<5	309	<2	20	<5	26.8	LAD-PCI	10
2014.06	a70	M	70	UA	Yes	No	1.3	2.45	9.5	213	4.15	2		1.37	5.7	<5	478	3.33	35	<5	67.8	LAD-PCI	15
2014.07	a71	M	58	UA	Yes	Yes	1.09	1.71	9.2	195	6.95	1	71	1.14	9.6	<5	636	11.6	163	<5	61.9	LCX-PCI	18.5
2014.07	a72	F	79	STEMI	Yes	No	1.06	3.3	5.3	152	5.8	2	60.2	17.75	6.5	1.3	831	2.86	16	1.06	13.2	LAD-PCI	38.5
2014.07	a73	F	61	UA	Yes	Yes	1.41		6.1	85	6.08	2		0.91	6.9	<5	525	<2	18	<5	14.4	LCX-PCI	8
2014.07	a74	M	60	UA	Yes	No	1.83	2.92	6.8	217	4.91	3		0.83	5.9	<5	209	<2	70	<5	54.1	D1-PCI	10
2014.07	a75	F	83	CAD	Yes	No	1.03	2.36	7.7	191	4.21	2		1.36	5.3	<5	787	4.84	91	<5	103	LCX-PCI	9
2014.07	a76	M	59	UA	Yes	No	0.92	1.48	5.6	140	3.03	1	66.9	0.99	5.3	<5	435	<2	46	<5	35.5	RCA-PCI	39
2014.07	a77	F	65	CAD	Yes	Yes	1.35	2.5	7.2	161	4.43	3		1.39	6.5	<5	195	<2	40	<5	29.8	D1-PCI	43
2014.07	a78	M	61	UA	Yes	Yes	0.97	2.75	14.5	183	5.11	3		13	7.8	<5	316	<2	50	<5	29	LAD-D-PCI	21
2014.07	a79	M	61	After P	Yes	No	1.27	2.52	5	206	4.2	3	60	1.05	5.4	<5	479	5.71	26	<5	38.8	LAD-PCI	10
2014.07	a80	M	59	UA	No	No	0.96	1.47	9.1	190	2.81	1	69	4.13	5.7	<5	466	6.84	179	<5	88.6	LAD-PCI	10
2014.07	a81	F	82	AMI	Yes	No			7.57	239	5.84		63	22.63	5.9	<5	328	4.68	13	11.8	23.9	RCA-PCI	16
2014.07	a82	M	76	AMI	No	No	1.5	3.87	3.54	134	6.17	4	71	2.27	6.3	<5	649	3.34	34	<5	29.4	LAD-PCI/D2-PCI	51.5

2014.07	a83	M	74	UA	No	No	1.54	2.71	5.06	92	4.55	3	45.5	7.59	6.1	<5	829	8.52	19	<5	19.4	RCA-PCI	57.5
2014.07	a84	M	75	UA	No	Yes	1.35	2.04	4.9	163	4.16	2	57	1.44	6	<5	420	<2	12	<5	17.4	LAD-PCI	36
2014.07	a85	F	62	After P	Yes	Yes	1.79	1.75	7.4	141	3.48	2		<8		<5	278	3.01	25	<5	41.6	LAD-PCI	33.5
2014.07	a86	M	61	After P	Yes	No	1.3	3.15	16.9	244	5.15	3	75	<8	5.4	<5	550	2.99	19	<5	46.6	LAD-PCI	22
2014.07	a87	F	66	UA	Yes	Yes			7.7	325	7.99			1.31	8	<5	270	2.82	44	<5	34.4	RCA-PCI	34.5
2014.07	a88	M	68	CAS	Yes	No	1.59	3	9.3	200	4.92	3		3.82	5.9	<5	494	<2	18	15.5	30.3	RCA-PCI	14
2014.07	a89	F	58	CAD	Yes	No	0.94	1.9	7.2	225	3.4	2	73	1.1	6.1	<5	377	<2	25	<5	41	LCX-PCI	16
2014.07	a90	F	57	CAD	No	No	1.21	2.25	5.4	214	4.29	2	69	1.44	6.5	<5	292	<2	28	<5	25.2	LAD-PCI	14
2014.07	a91	M	60	UA	Yes	No	1.06	1.92	10	206	3.62	2		3	5.3	<5	439	4.35	51	<5	39.4	RCA-PCI	34.5
2014.07	a92	M	62	UA	Yes	No	0.7	2.34	8	191	4.28	2		37.46	6.3	<5	812	29.3	27	<5	40.7	LAD-PCI	18
2014.07	a93	M	59	UA	No	Yes	0.98	1.89	5.8	164	3.49	2	64.9	3.16	9	<5	559	4.19	12	<5	11.3	RCA-PCI	14
2014.07	a94	M	77	ACS	Yes	Yes	3.5	2.13	8.6	206	17.5	1		9.38	9.9	7.7	486	15.9	83	<5	38.1	LCX-OM-PCI	25
2014.07	a95	F	52	UA	No	Yes	1.31	2.31	8.9	230	4.16	2	60	1.71	10.1	<5	411	2.22	42	<5	58.3	RCA-PCI	22
2014.07	a96	M	81	CAD	Yes	No	1.27	2.69	8.5	216	4.49	3	72	2.22	5.7	<5	707	2.79	60	<5	31.2	LAD-PCI	14
2014.07	a97	M	56	MI	Yes	No	1.18	2.11	9.6	150	3.9	2	65	1.43	4.9	<5	471	2.34	129	<5	82.3	LAD-PCI	25
2014.07	a98	M	67	CAD	No	No	1.34	3.47	6.03	250	5.17	3	70.3	2.61	5.6	<5	381	<2	60	<5	38.7	LAD-PCI	23
2014.07	a99	F	72	CAD	Yes	No	1.55	3.4	7.7	479	5.79	3	69	8.75	5.9	<5	277	4.7	15	<5	20.5	LCX-PCI	22
2014.07	a100	M	63	After P	No	No	1.33	3.37	9.7	255	6.34	4		1.95	5.6	<5	408	3.73	<5	<5	70.7	LAD-PCI/LCX-PCI	16
2014.07	a101	F	65	UA	Yes	No	1.18	1.89	8.7	162	3.53	2		3.06	6	<5	632	<2	18	<5	42.5	LAD-PCI/Stent recanalization	13
2014.07	a102	M	55	UA	Yes	Yes	0.9	2.4	5	233	12.22	5		1.31	9.3	<5	332	<2	56	<5	52.7	LAD-PCI/RCA-PCI	22
2014.07	a103	M	63	UA	Yes	No	1	1.1	8	219	5.61	1	67	0.97	6.2	<5	465	4.3	248	<5	150	LAD-PCI	15
2014.07	a104	F	82	ACS	Yes	No	2.39	3.39	7	116	4.87	1	69	1.22	6.1	0.4	521	5.29	10	1.14	13.4	LCX-PCI/LAD-PCI	25
2014.07	a105	F	67	UA	Yes	No	1.28	3.29	4.6	177	4.79	1	73.9	<8		<5	313	<2	21	<5	34.3	LAD-PCI	10
2014.08	a106	M	82	UA	Yes	No	1.18	1.91	6.1	159	3.18	2	48	0.09	6	6.9	517	<2	199	<5	85.4	RCA-PCI	14
2014.08	a107	F	73	UA	Yes	No	1.38		5.9	224	4.63	3	63	<8	5.4	<5	355	<2	6.8	<5	8	LAD-PCI/RCA-PCI	24
2014.08	a108	F	66	CAD	No	No	1.22	3.76	3.64	161	5.64	4	67	1.98	5.3	<5	632	7.62	58	5.11	112	D1-PCI	9

2014.08	a109	M	66	UA	No	Yes	1.07	1.97	5.02	113	3.27	2		6.16	7	<5	506	4.48	71	<5	123	LAD-PCI	34
2014.08	a110	M	62	UA	Yes	No	1.16	2.1	5.5	231	3.37	2	74	0.47	6.3	<5	232	<2	49	<5	18.1	LAD-PCI	36
2014.08	a111	F	54	UA	Yes	Yes	1.32	3.27	6.9	170	5.09	3		2.63	11.9	<5	342	<2	20	<5	21.5	PLA-PCI	4
2014.08	a112	F	72	UA	Yes	Yes	1.15	1.08	7	165	2.59	1		3.65	8.4	<5	383	3.54	96	<5	79.7	LAD-PCI	17
2014.08	a113	M	74	UA	Yes	No	1.32	3.6	4.42	221	5.5	4	64	1.76	5.5	<5	414	2.86	34	<5	49.9	LAD-PCI	15
2014.08	a114	M	51	MI	No	No	1.05	2.17	6	145	4.13	2	74.6	10.95	5.5	<5	365	4.59	52	<5	57	LAD-PCI	17
2014.08	a115	M	66	After s	Yes	No	1.05	1.73	6.7	105	2.86	2	55	1.09	5.9	<5	436	13.6	52	<5	49.4	LCX-PCI	23
2014.08	a116	M	76	UA	Yes	No	1.17	1.76	5.4	141	3.46	2	67	4.76	5.9	<5	497	6.37	55	<5	75.6	RCA-PCI	26
2014.08	a117	M	77	After s	Yes	No	1.19	1.23	6.7	140	2.45	1		<8	5.5	<5	530	3.78	76	<5	29.6	LAD-PCI	31.5
2014.08	a118	F	61	After s	Yes	No	1.49	1.73	6.7	123	6.72	2		0.83	6.8	<5	275	2.6	120	<5	82.5	LAD-PCI	27.5
2014.08	a119	M	76	UA	No	No	0.96	1.54	8.1	254	3.43	2		1.63	6	<5	523	2.27	357	<5	83.6	LAD-PCI	14
2014.08	a120	F	59	UA	No	Yes	0.92	1.11	8.1	219	2.24	1	75	1.74	7.3	<5	379	<2	50	<5	39.4	LAD-PCI	10
2014.08	a121	M	73	CAD	Yes	Yes	0.82	1.16	8.4	155	3.07	1	60	<8	8	<5	388	2.19	57	<5	41.5	RCA-PCI	25
2014.08	a122	F	76	CAD	Yes	No	1.31	2.77	5.1	134	4.86	3	62.5	<8	6.1	<5	269	<2	31	<5	24	LAD-PCI	13
2014.08	a123	M	58	After s	Yes	Yes	0.85	1.86	7.3	247	3.32	2	70.3	<8	7.4	<5	456	2.69	75	<5	74.2	RCA-PCI	20
2014.08	a124	M	74	UA	No	No	1.21	1.95	6.2	221	3.01	2	65	0.83	6.4	<5	423	<2	201	<5	201	RCA-PCI	7
2014.08	a125	M	56	After s	Yes	No	1.08	1.61	5.77	141	3.41	2		0.83	6	<5	435	<2	84	<5	86	LAD-PCI	20
2014.08	a126	F	65	UA	Yes	No	1.45	2.49	6.08	171	4.16	2	63	3.16	6.2	<5	333	3.66	72	<5	72.3	LAD-PCI	9
2014.08	a127	F	67	ACS	Yes	Yes	0.96	1.5	6.4	202	2.81	2	51	10.63	6.6	<5	438	5.2	108	<5	52.3	LAD-PCI/RCA-PCI	23
2014.08	a128	F	80	UA	Yes	No	1.07	1.72	4.1	95	3.01	2	66.9	6.19	7.7	<5	909	8.92	105	6.81	122	LCX-PCI	13
2014.08	a129	M	64	UA	Yes	Yes	1.28	2.47	4.9	201	4.22	2	70	3.08	6.8	<5	397	<2	65	<5	108	LAD-PCI	10
2014.08	a130	F	80	UA	Yes	Yes	0.9	2.03	6.9	135	3.41	2	27.6	9.77	8	<5	609	4.06	73	<5	40.4	RCA-PCI	15
2014.08	a131	M	48	AMI	Yes	No	0.68	1.77	9.4	207	3.23	2		3.02	6.2	<5	535	2.11	57	<5	116	LAD-PCI	13
2014.08	a132	F	59	UA	Yes	No	0.9	2.31	6.3	149	4.47	2	74	4.09	5.7	<5	412	5.43	64	<5	33.5	LAD-PCI	16
2014.08	a133	F	58	UA	Yes	Yes	1.11	2.68	4.4	192	4.56	3	68.5	0.83	7.2	<5	329	<2	<5	<5	27.9	LAD-PCI	9
2014.08	a134	M	67	UA	Yes	No	1.15	2.87	4.34	162	4.57	3		1.39	5.5	<5	373	2.33	56	<5	87.8	LCX-PCI	5
2014.08	a135	M	61	CAD	No	No	1	2.61	7.9	172	17.9	3		<8	6	<5	382	3.86	80	<5	92.6	RCA-PCI	5
2014.08	a136	M	55	ACS	Yes	Yes	1.04	1.36	9.5	206	5.2	3	68	2.3	6.9	2.2	575	3.11	100	1.55	86.7	LAD-PCI/LCX-PCI	22
2014.08	a137	M	61	MI	No	Yes	1.32	1.39	10	198	6.08	1		<8	7.2	<5	355	<2	20	<5	29.2	LAD-PCI	26

2014.08	a138	M	61	STEMI	No	Yes	1.61	1.73	12.1	159	6.7	1	59.8	64.14	7.9	<5	445	<2	49	<5	49.8	LAD-PCI	29.5
2014.09	a139	F	78	UA	Yes	No	0.99	1.42	7.68	266	7.91	2	60	19		0.8	406	2.89	22	2.59	26.3	RCA-PCI	7
2014.09	a140	M	54	UA	Yes	Yes	0.81	1.66	10.43	353	4.62	3	60	2.59	9	1	571	3.7	17	1.08	10.3	LAD-PCI	16
2014.09	a141	M	78	UA	Yes	Yes	1.66	2.85	6.2	229	5.4	1		16.19	8.5	<5	705	17.8	66	<5	83.9	LAD-PCI	28
2014.09	a142	M	57	UA	Yes	Yes	0.86	1.37	8.3	137	3.09	1	56	4.53	6.3	82	532	13.2	###	1.58	162	LM-PCI	32
2014.09	a143	F	77	UA	No	No	1.18	2.71	7.5	173	4.55	3	62	4.94	5.8	54	362	36.7	317	1.65	88.8	LAD-PCI	23.5
2014.09	a144	M	83	UA	Yes	No	0.008	2.04	8.3	328	3.35	2		7.52	5.6	1.8	1313	11.2	158	1.95	68.9	LCX-PCI	20
2014.09	a145	M	65	UA	Yes	No	0.86	2.06	4.2	198	3.65	2	69.2	1.03	5.8	5.3	348	2.86	105	2.28	54.4	RCA-PCI/LCX-PCI	22
2014.09	a146	M	67	UA	No	No	1.3	1.52	8.8	202	3.06	2		0.91	5.6	1.3	315	10.6	100	4.63	38.7	RCA-PCI	26
2014.09	a147	M	50	UA	Yes	No	1.19	2.99	8.8	301	4.94	3	78	0.91	5.6	1.8	333	5.87	187	1.19	110	LCX-PCI	18
2014.09	a148	F	76	CAD	Yes	No	0.97	2.38	7.41	145	4.39	2		3.04	6.6	1.9	540	5.03	114	1.37	137	RCA-PCI	20
2014.09	a149	M	69	CAD	Yes	Yes	1.15	3.37	5.79	250	5.25	3	67	1.7		2.3	332	2.66	73	1.68	163	LAD-PCI	27
2014.09	a150	M	69	UA	Yes	Yes	1.18	2.08	6.8	168	4.76	2		<8		2.4	424	2.56	100	0.91	61.6	LAD-PCI	23
2014.09	a151	M	70	ACS	No	Yes	1.21	3.05	10	275	5.93	3	30	30	6.5	3.2	1059	3.75	53	1.49	55	LCX-PCI	30.5
2014.09	a152	F	63	UA	Yes	Yes	1.47	2	7	208	0.48	2	71	1.12	6.6	0.5	323	1.21	17	21.5	13.2	LCX-PCI	6
2014.09	a153	M	66	UA	No	No	1.31	1.48	6.5	144	6.05	1		0.92	5.8	0.4	373	1.62	14	0.39	18.5	LAD-PCI	18
2014.09	a154	F	65	NSTEMI	Yes	No	1.2	3.9	8.4	209	5.81	1	66	<8	6.3	1.5	320	8.77	13	0.95	11	LAD-D-PCI	4
2014.09	a155	M	73	ACS	Yes	No	1.16	1.97	7.23	211	3.25	2	57	1.47	5.3	1.4	504	5.86	58	3.76	105	PDA-PCI	4
2014.09	a156	M	43	UA	No	No	1.4	2.59	8.73	249	4.56	3		4.31	5.8	1.3	287	13.3	95	1.22	38.6	LAD-PCI	11
2014.09	a157	M	63	UA	Yes	No	1.36	3.87	7.6	226	6.05	4		<8		2.3	464	2.98	102	1.39	98.9	LCX-PCI	44
2014.09	a158	M	53	AMI	Yes	No	0.93	3.07	7.7	177	4.78	3	58	<8	5.6	0.5	474	7.83	31	0.83	63.7	RCA-PCI	22
2014.09	a159	M	59	CAD	Yes	Yes	1.05	1.48	4.9	221	2.64	1	65	0.83	6.2	1.3	366	1.65	68	1.74	62.3	LAD-PCI	22
2014.09	a160	M	78	AMI	Yes	No	0.95	2.59	6.2	168	4.17	3	29.1	<8	5.8	1.5	606	4.6	119	1.35	85	LAD-CTO-PCI	24.5
2014.09	a161	M	58	UA	Yes	Yes	0.99	2.19			3.55	2				0.2	245	3.66	21	0.8	11	LCX-PCI,LAD-PCI	15
2014.09	a162	F	73	CAD	Yes	No	1.47	3.44	4.5	173	5.35	3		2.66	5.6	1	879	2.87	59	1.17	73.9	LAD-PCI	20
2014.09	a163	F	78	UA	Yes	Yes	0.67	0.69	3.6	146	4.81	1	69	1.07	8.8	0.6	267	0.67	11	1	27.4	OM-PCI	12
2014.10.	a164	M	71	CAD	Yes	No	1.15	1.91	10.5	210	4.46	2		11.3	5.7	1.7	649	9.6	112	1.1	177	LAD-D1-PCI	14
2014.10.	a165	F	82	CAD	No	Yes	1	1.49	5.08	134	8.1	1		3.21	8	0.8	519	4.21	33	7.46	35.5	RCA-PCI?LCX-PCI	35

2014. 10.	a166	M	47	MI	Yes	No	1.04	2.6	6.05	361	5	1		3.92	5.7	0.2	434	1.89	10	0.54	8.9	RCA-PCI/LCX-PCI	23
2014. 10.	a167	F	80	ACS	Yes	No	1.51	4.28	8.4	305	4.98	1		10.32	6.8	2.2	219	13.3	114	0.83	40.1	RCA-PCI/LCX-PCI	18
2014. 10.	a168	F	72	ACS	Yes	Yes	1.69	2.18	8.6	232	7.59	1		3.9	9.8	1	436	2.55	72	0.96	32.4	LAD-PCI/LCX-PCI	37
2014. 10.	a169	M	63	ACS	Yes	No	2.5	2.22	6.2	197	5.27	1	67		5.6	0.5	411	2.09	28	1.23	30.4	LAD-PCI	5
2014. 10.	a170	F	80	CAD	No	No	1.44	3.36	8.76	271	5.97	1	67	9.58	6.6	2	448	4.44	55	2.44	35.4	LAD-PCI	7
2014. 10.	a171	M	71	ACS	Yes	No	1.11	1.49	8.9	153	5.13	2	71.8	1.26	6.3	2.3	487	4.48	182	1.79	150	LCX-PCI/OM-PCI	6
2014. 10.	a172	M	63	CAD	Yes	No	1.13	1.8	9.8	188	4.96	2		1.05	6.4	1.6	271	1.79	82	1.08	88.2	LAD-PCI,RCA-PCI	15
2014. 10.	a173	F	57	UA	Yes	No	1.56	3.84	8?25	259	5.93	4		0.83	5.5	2.2	281	1.99	128	0.81	94.1	LAD-PCI	10
2014. 10.	a174	M	65	UA	No	No	1.64	2.94	5.1	162	4.83	3	70	0.32	5.5	5.7	374	8.78	158	1.37	117	LAD-PCI?LCX-PCI	19
2014. 10.	a175	M	67	UA	No	No	1.31	2.11	11.6	270	4.88	2	66	1.14		1.1	275	11.1	20	1.08	19.8	LAD-PCI/RCA-PCI	22
2014. 10.	a176	M	72	CAD	Yes	No	1.15	4.33	4.63	172	6.8	4	66	<8	5.7	1.9	514	4.1	88	1.61	25.9	LAD-PCI/LCX-PCI	27
2014. 10.	a177	F	55	ACS	Yes	No	1.26	2.42	6.4	168	4.21	2		4.62		1.7	336	1.67	127	1.31	86	RCA-PCI	15
2014. 10.	a178	M	59	NSTEI	No	No	0.97	1.87	7.6	213	4.57	1		0.83		3.3	437	10.6	257	1.23	90.8	LCX-PCI	40
2014. 10.	a179	F	76	AMI	Yes	No	1.42	2.62	5.4	141	2.92	1		119.56	9.4	1.1	269	19.2	0.7	16.2	18.7	RCA-PCI	20
2014. 10.	a180	M	71	CAD	Yes	No	1.15	1.91	10.5	210	4.46	2		11.3	5.7	1.7	649	9.6	112	1.1	177	LAD-D1-PCI	16
2014. 10.	a181	M	71	CAD	Yes	No	1.03	2.47	7.8	236	5.74	2		<8		1.4	772	8.33	121	1.71	64	LAD-PCI	12
2014. 10.	a182	F	62	CAD	Yes	No	0.91	3.18	5.9	197	10.93	3		0.83	9.4	2.1	426	4.58	137	1.51	77.5	LAD-PCI	15
2014. 11	a183	F	74	ACS	Yes	No	1.12	2.25	4.7	212	4.15	2	73	1.76	5.9	0.6	486	11.9	26	0.88	39.5	RCA-PCI	17
2014. 11	a184	M	55	UA	No	No	1.07	2.61	6.98	236	5.3	3	71	1.73	5.5	0.6	387	2.11	16	0.43	21.7	LAD-PCI	10
2014. 11	a185	F	85	CAD	Yes	Yes	0.96	1.71	4.68	146	3.24	1	63	<8	8.9	0.5	778	5.43	49	4.8	49.5	LCX-PCI	19
2014. 11	a186	F	78	UA	Yes	No	1.31	2.43	8	119	4.34	2	78	6.81	6	1.8	522	6.38	16	3.82	34.9	LCX-PCI	28
2014. 11	a187	F	81	UA	Yes	No	1.81	2.94	4.36	237	5.04	3	59	1.42	5.6	0.5	435	7.59	6.6	1.29	16.4	RCA-PCI	22
2014. 11	a188	M	77	After c	Yes	No	1.11	2.5	8.5	184	4.04	3	66.1	<8		2.3	521	2.78	90	1.62	57.3	RCA-PCI	30
2014. 11	a189	M	78	UA	Yes	No	0.007	2.84	5.51	198	4.8	3		1.96	5.4	1.2	367	2.49	89	1.38	52.9	LAD-PCI	13
2014. 11	a190	F	80	Corona	Yes	No	1.02	2.52	4.7	212	4.25	2	55	102.51	5.9	4.1	360	17.3	53	3.53	27	LAD-PCI	15

2014.11	a191	M	71	MI	Yes	No	1.32	3.22	1.9	171	5.3	3	73.8	1.02	5.1	0.3	368	2.09	14	1.37	30.6	RCA-PCI	25
2014.11	a192	F	53	UA	No	No	1.31	2.35	8.3	193	4.42	2		<8		1.5	312	2.62	33	1.14	18.8	LAD-PCI	23
2014.11	a193	M	57	CAD	No	No	1.19	2.24	5.7	197	4.15	2	73.4	1.9	5.6	1.5	375	3.14	91	1.04	65.3	LAD-PCI/RCA-PCI	18
2014.11	a194	M	56	ACS	No	No	1.09	1.46	6	161	2.59	2		1.63	6.2	0.5	379	2.48	10	1.29	13.3	LAD-PCI	21
2014.11	a195	M	77	CAD	Yes	Yes	0.88	2.23	4.1	210	3.89	2	65	9.36	5.5	0.6	716	3.55	17	2.08	29.9	RCA-PCI	29
2014.11	a196	F	76	ACS	Yes	No	0.9	1.99	5.8	195	5.1	3	40	1.6	7.6	0.4	894	3.61	17	1.32	21.1	LCX-PCI	5
2014.11	a197	F	84	ACS	No	No	1.45	2.79	5.45	155	4.13	3	41	1.23	5.2	0.3	618	3.22	15	0.91	24.1	LAD-PCI	39.5
2014.11	a198	F	78	UA	No	No	1.38	2.36	4.7	216	5.01	2		0.97	5.8	0.4	394	2.74	32	0.6	32.2	LAD-PCI	7
2014.11	a199	M	64	UA	No	No	0.96	2.7	7.4	240	5.47	2	58	0.9	6.1	0.7	329	1.99	14	0.73	28.4	LCX-PCI	18
2014.11	a200	M	69	STEM	Yes	No	1.3	2.77	10.2	286	6.4	4	53.1	102.68		0.4	641	3.46	12	2.27	7.99	RCA-PCI	20
2014.11	a201	F	79	UA	Yes	No	1.26	3.2	5	207	4.96	1	61	0.57	5.8	1.7	485	2.77	23	1.07	18.5	LCX-PCI	14
2014.11	a202	M	78	UA	Yes	No	1.21	1.33	7.1	174	5.24	1	47	1.54	5.6	0.9	679	10.9	25	1.18	18.9	LCX-PCI	22
2014.11	a203	F	67	UA	Yes	No	1.38	2.58	7.24	294	5.7	1		1.6	6	0.3	359	2.52	20	0.44	19.8	RCA-PCI	8
2014.11	a204	M	56	UA	No	No					6.64				5.9	2.5	446	2.51	210	1.15	126	LM-LAD-PCI	24
2014.11	a205	F	62	MI	Yes	No	1.37	1.92	6	128	4.87	1	65	19.05	5.3	3.5	503	78.7	185	88.6	79.7	LCX-PCI	22.5
2013.11	a206	M	72	AMI	Yes	Yes	1.17	2.42	8.8	212	5.32	4	47	0.95	6.6	<5	452	2.68	204	<5	89	LAD-PCI/LCX-PCI	23
2014.11	a207	M	56	UA	No	No	1.94	1.46	6	161	2.59	1	66	<8	6.2	0.5	379	2.48	10	1.29	13.3	LAD-PCI	22
2014.11	a208	M	56	UA	Yes	No	0.99	1.45	6.96	179	5.3	1		0.83	5.4	0.2	347	2.15	7.1	0.73	10.5	LAD-PCI	19
2014.11	a209	M	52	UA	Yes	Yes	0.98	1.56	7.5	208	6.19	1	64	1.36	6.6	0.9	387	1.7	58	0.51	38.8	LCX-PCI	23
2014.11	a210	M	61	CAD	Yes	No	1.52	2.42	7.1	159	5.36	3		0.42	5	0.5	414	1.84	6.7	1.66	8.93	LAD-PCI	15
2014.11	a211	M	57	CAD	No	No	1.19	2.24	5.7	197	5.04	3	73.4	1.9	5.6	1.5	375	3.14	91	1.04	65.3	LAD-PCI	18
2014.11	a212	F	57	CAD	Yes	No	1.57	3.17	4	165	5.76	1		0.51	5.9	0.5	209	1.24	7.2	0.52	6.79	LAD-PCI	10
2014.11	a213	M	63	UA	No	No	1.4	1.93	5.03	177	4.77	1	52			0.1	437	2.15	7.6	1.49	6.92	LAD-PCI	15
2014.11	a214	F	81	UA	Yes	No	1.81	2.94	4.36	237	5.39	1	59	1.42	5.6	0.6	551	7.95	17	0.83	26	RCA-PCI	22
2014.11	a215	M	60	ACS	Yes	Yes	1.4	3.19	9.4	228	8.21	2		67	7.1	1.6	518	9.71	25	0.67	28.5	D1-PCI	29
2014.12	a216	M	52	CAD	Yes	Yes	0.91	2.02	6.7	194	10.2	1	67	1.01	10.8	0.1	487	3.94	20	0.7	8.87	RCA-PCI	30
2014.12	a217	M	62	ACS	Yes	No	0.94	1.52	7.4	136	5.2	2		<8		0.3	597	3.01	9.2	1.32	10.4	LAD-PCI	26
2014.12	a218	M	50	CAD	No	No	1.6	3.67	6	168	4.84	1		<8	5.7	7.8	228	2.26	9.1	0.39	8.21	RCA-PCI	17
2014.12	a219	M	83	NSTEM	No	Yes	0.78	2.06	9.6	219	6.8	1		21.45	6.4	0.6	2459	7.12	9	2.6	18	LCX-PCI	22

2014.12	a220	F	81	ACS	No	No	1.54	3.11	4.7	234	4.39	1	77	0.72	5.7	3.1	340	2.26	28	2.03	17.5	LAD-PCI	20
2014.12	a221	F	91	UA	Yes	No	1.41	1.48	6.6	192	5.32	1	71.5	1.28	5.3	0.6	864	3.59	15	11.4	12.4	LAD-PCI	9
2014.12	a222	M	68	CAD	No	No	1.46	2.36	5.8	284	4.9	1		7.62	5.7	0.3	827	2.74	9.8	1.33	19.3	RCA-PCI	21
2014.12	a223	M	65	ACS	Yes	Yes	1.39	2.94	7.5	208	7.74	1	64.4	1.31	8.5	1.4	379	1.92	14	1.67	12.2	LCX-PCI	5
2014.12	a224	M	75	ACS	No	No	0.81	1.45	7.8	107	4.59	1	65	21.16	5.5	0.9	1024	10.5	20	34.1	22.4	RCA-PCI	42
2014.12	a225	M	62	ACS	No	No	1.42	1.22	8.8	207	4.98	1	66.9	6.38	5.9	1.4	458	2.73	16	2.13	15.5	LAD-PCI	15
2014.12	a226	M	61	STEMI	Yes	Yes	1.56	2.23	7.1	193	4.99	2	54	0.96	5.9	0.2	574	1.4	17	0.77	15.4	LAD-PCI	27.5
2014.12	a227	M	58	ACS	No	Yes	0.84	0.94	8.8	143	6.19	1		1.09	6.4	0.4	474	3.02	12	5.17	11	LAD-PCI	21
2014.12	a228	M	66	CAD	No	No	1.31	2.88	5.25	180	5.66	1	67	2.39	6.2	0.2	378	2.23	8.4	1.52	8.55	RCA-PCI	16
2014.12	a229	M	58	CAD	Yes	Yes	1	1.75	8.9	212	4.06	1	73.5	<8		0.5	439	3.38	11	0.87	7.79	LAD-PCI	30
2014.12	a230	F	65	UA	No	Yes	1.13	1.63	6.7	263	9.02	2	68	<8		0.2	358	4.91	8.3	0.41	9.35	LAD-PCI	17
2014.12	a231	M	63	NSTEM	Yes	No	1.02	2.51	7.7	156	4.76	1		<8	6.4	0.7	404	4.29	21	3.12	28	LAD-PCI/LCX-PCI	39.5
2014.12	a232	M	56	UA	No	No	1.24	1.52	7.34	233	6.2	2		1.17	6.4	0.3	274	2.94	18	1.3	58.8	LAD-PCI	9
2014.12	a233	M	82	ACS	Yes	No	1.84	3.49	6.5	310	6.23	1	67	2.62	6.2	0.1	728	3.79	7.1	0.54	10.6	OM-PCI	4
2014.12	a234	M	49	ACS	No	Yes	0.92	2.66	6.9	186	11.16	2		4.19	8.9	0.4	630	3.07	10	4.13	22.3	RCA-PCI	14
2014.12	a235	F	69	UA	Yes	No	1.56	3.13	5.98	233	6.21	1	69	3.59	6.4	0.3	489	2.13	17	2.01	12.7	LAD-PCI	10
2014.12	a236	F	68	UA	Yes	No	1.66	1.53	4.6	126	4.29	1		0.83	5.5	0.3	382	2.06	6.3	1.01	8.43	RCA-PCI	21
2014.12	a237	M	62	CAD	Yes	No	1.32	2.85	7.8	139	4.49	4	67	2.24	5.8	0.3	256	4.38	12	0.67	18.5	LAD-PCI	9
2014.12	a238	F	70	ACS	Yes	No	1.2	1.68	5.94	180	6.08	1	77	1.74	6.6	2.5	577	4.92	18	1.33	23.9	RCA-PCI	14
2014.12	a239	F	74	ACS	No	No	1.56	3.84	6.8	210	5.98	1		<8		0.4	528	2.24	13	1.09	11.7	LAD-PCI	22
2014.12	a240	M	59	NSTEM	Yes	No	1.07	2.53	8.9	182	5.12	1		9		0.6	447	5.83	27	0.95	22.7	LAD-PCI	30
2014.12	a241	F	60	NSTEM	Yes	No	1.75	3.44	5.93	235	5.4	1		0.83	6.6	0.8	256	2.56	32	2.53	19	LAD-PCI	23
2014.12	a242	M	85	Syncop	Yes	Yes			6.1	102	5.16		71	12		1	644	3.23	13	0.56	8.58	LAD-PCI	12
2014.12	a243	M	84	ACS	Yes	No	0.82	1.46	8.1	182	5.2	3		<8	5.5	0.3	684	10.1	21	1.31	23.9	RCA-PCI	27
2014.12	a244	M	60	CAD	No	Yes	1.12	3.02	7.6	220	8.34	1	75	<8		0.1	486	1.56	6.7	1.43	6.29	LAD-PCI	15
2014.12	a245	M	62	UA	Yes	Yes	1.09	2.15	10.2	200	7.15	2		<8		0.6	345	7.25	4.1	0.88	8.58	LAD-PCI	12
2014.12	a246	M	70	ACS	Yes	No	0.91	1.39	7.6	220	5.63	1		<8		0.3	416	4.18	12	0.63	9.74	RCA-PCI	23.5
2014.12	a247	M	64	UA	No	No	1.14	2.28	10.1	139	5.01	1		<8	5.7	0.6	620	1.56	14	10.7	13.8	LAD-PCI	10
2014.12	a248	M	72	STEMI	No	No	1.56	2.64	13	211	5.52	1	56	136.45	5.5	0.8	764	20.4	49	6.39	60.6	LAD-PCI	33.5
2014.12	a249	M	51	NSTEM	Yes	No	3.29	1.09	8.1	210	6.06	1	59.7	4.94	5.6	0.4	354	32.3	11	0.85	9.26	LCX-PCI	30

2015.01	a250	M	38	ACS	Yes	No	1	3.87	8.5	93	6.04	3	71	0.83	5.4	0.3	1044	2.64	28	1.25	22.6	LCX-PCI	20
2015.01	a251	F	80	CAD	Yes	No	1.41	2.78	4.4	179	4.79	1	60	1.45	6	0.5	441	5.36	46	0.66	26.8	LCX-PCI	33
2015.01	a252	M	58	CAD	Yes	No	1.32	2	8.1	231	4.51	3	66	0.83	5.8	0.4	549	1.87	18	1.12	9.29	LAD-PCI	31.5
2015.01	a253	M	64	UA	Yes	No	1.2	1.75	7	151	4.79	2	60	2.15	5.8	1.2	332	3.53	47	0.65	44.3	RCA-PCI	30
2015.01	a254	M	60	CAD	No	No	1.5	4.62	9	222	6.31	1	73	1.95	6	0.9	259	7.47	19	1.15	18.6	RCA-PCI	22
2015.01	a255	M	61	UA	Yes	No	0.95	2.08	6.2	203	4.38	2		1.32	5.5	0.4	436	3.24	6.3	0.7	10.5	LAD-PCI	10
2015.01	a256	M	72	ACS	No	Yes	1.2	3.36	6.2	196	3.87	1	68	3.1	6.8	0.7	381	5.77	17	0.58	14.4	LAD-PCI	10
2015.01	a257	M	67	ACS	Yes	Yes	0.92	2.12	10.3	292	5.01	1	63	8.46	6.4	0.4	288	4.92	12	0.64	20.1	LAD-PCI	35
2015.01	a258	M	75	STEMI	Yes	No	1.34	2.93	5.8	117	5.02	1	71	6.35	6.1	0.7	530	3.64	31	0.77	43.8	RCA-PCI	30
2015.01	a259	M	68	ACS	Yes	No	1.08	2.25	6.1	297	5.48	1		<8		1.2	1822	6.31	22	0.57	58.8	RCA-PCI	15
2015.01	a260	M	60	CAD	Yes	Yes	0.9	1.29	6.3	257	3.65	1	60	45.46	7.4	1.1	774	7.1	23	9.88	24.4	LAD-PCI	28.5
2015.01	a261	M	74	STEMI	No	No	1.46	3.45	8.3	163	5.76	1	65	3.08	6.1	0.6	237	15.8	9.5	0.47	9.34	LAD-PCI	10
2015.01	a262	M	78	MI	No	No	138	1.95	5.11	178	4.83	1		0.83	5.7	0.5	546	2.01	11	0.89	21.9	LCX-PCI/RCA-PCI	28
2015.01	a263	F	76	UA	Yes	No	1.08	4.07	6.3	169	6.66	7	78	1.77	6	0.8	369	3.17	21	0.84	16.2	LCX-PCI	6
2015.01	a264	M	65	UA	Yes	No	1.06	1.56	4.9	167	5.68	2	62	1.28	6.3	0.2	410	3.32	19	3.08	66.2	LAD-PCI	33.5
2015.01	a265	F	66	UA	No	Yes	1.33	1.23	5.59	194	6.06	1	66.1	1.58	7.1	0.6	421	2.07	21	1.91	22.8	LAD-D-PCI	12
2015.01	a266	F	65	CAD	No	No	1.38	3.71	6	193	4.85	1		<8		0.1	675	3.05	8.3	0.45	11.6	LM-LAD-PCI	27
2015.01	a267	M	67	NSTEM	No	No	1.84	3.3	5.5	174	4.75	1	68	<8		0.2	464	1.19	6.9	1.36	9.12	RCA-PCI/LAD-PCI	29.5
2015.01	a268	M	83	UA	Yes	Yes	1.34	1.29	8	138	3.43	1	59	<8	5.2	1	576	8.99	39	1.17	40.8	RCA-PCI	36
2015.01	a269	M	56	STEMI	No	No	0.92	3.1	7.45	236	8.58	1	75	118.57	8	0.2	449	2.02	14	1.3	18.7	LCX-PCI	16
2015.01	a270	M	73	UA	Yes	Yes	0.89	1.19	8.6	98	6.91	2	72	1.87	6.2	1.4	579	5.03	31	0.89	15.6	LCX-PCI	11
2015.01	a271	M	68	AMI	Yes	No	1.04	1.11	6.8	136	5.84	1	64	18.05		0.4	272	1.27	13	1.69	15.9	RCA-PCI	29.5
2015.01	a272	M	71	ACS	No	Yes	1.01	1.57	8.2	176	7.13	1		<8		0.3	491	3.19	8.4	0.91	14.2	LAD-PCI/OM3-PCI	32
2015.01	a273	M	53	ACS	Yes	No	0.92	3.35	9.3	165	5.81	1	58	21		0.6	508	6.22	6.9	0.44	8.87	LCX-PCI	17
2015.01	a274	F	78	ACS	Yes	No	1.28	1.7	6.4	121	4.2	1	69	0.9	5.5	0.4	454	2.79	28	0.63	45.6	RCA-PCI	10
2015.01	a275	F	82	ACS	Yes	No	1.31	2.41	7.34	202	4.35	1	73.5	3.83	5.9	5.5	466	6.1	109	<5	49.6	LAD-PCI	16
2015.01	a276	M	82	NSTEM	Yes	Yes	0.68	1.82	6.7	182	12.02	2	57	14.93	7.9	0.5	712	15.4	13	2.03	12.5	LAD-PCI?OM-PCI	26
2015.01	a277	M	67	ACS	Yes	Yes	0.99	2.09	7	118	3.52	2		<8	5.6	0.4	931	11.9	18	1.53	23.8	LAD-PCI	16

2015.01	a278	F	55	ACS	Yes	No	0.97	2.61	6.4	164	4.9	5	63	<8	5.7	0.2	1263	8.03	12	1.47	24.3	LAD-PCI	12
2015.01	a279	F	63	ACS	No	No	1.57	2.69	5.7	237	4.73	1	59.8	9.04	5.8	0.4	421	2.22	19	0.79	25.7	LM-LAD-PCI	25
2015.02	a280	M	70	STEMI	Yes	No	1.3	2.23	5.99	221	6.1	3	63	2.1	6.7	0.7	413	2.4	29	1.32	16	RCA-PCI	36.5
2015.02	a281	M	76	After P	Yes	Yes	0.81	2.11	8.1	217	8.67	1	65	67.14	13.3	0.6	742	15.9	11	3.89	13.7	RCA-PCI	10
2015.02	a282	M	71	After P	Yes	No	0.84	1.19	6.2	98	4.1	1	65	5.54	5.6	1.2	552	3.91	66	5.13	66.8	LAD-PCI	18
2015.02	a283	F	85	ACS	Yes	Yes	1.71	1.86	3.15	143	2.89	1	73.8	8.23	6.7	2.1	477	23.2	57	4.13	96.2	RCA-PCI	13
2015.02	a284	F	82	ACS	Yes	No	1.92	3.24	5.61	258	5.47	1	74.6	0.85	5.9	0.4	466	4.15	10	0.98	16.7	LCX-PCI/CA-PCI	28.5
2015.02	a285	F	64	UA	No	No	1.28	1.42	6.4	186	4.6	1	70	4.72	5.7	0.7	147	3.65	29	1.85	38.9	RCA-PCI	21
2015.02	a286	F	64	STEMI	No	Yes	0.94	2.75	4.5	177	10.58	2	64	10.88	9.5	0	477	3.68	13	0.99	16.6	RCA-PCI	31
2015.02	a287	M	57	UA	Yes	Yes	1.09	2.72	7.27	286	7.74	3	72	1.67	7.4	0.6	328	2.22	40	1.13	40.6	RCA-PCI	5
2015.02	a288	M	61	UA	No	No	1.15	2.11	6.4	211	5.06	1		1.21	4.9	0.3	474	3.86	22	1.13	33	RCA-PCI	5
2015.02	a289	M	63	CAD	no	No	1.03	1.8	8.5	325	5.04	1	67.9	1.18	6.3	1.1	1116	11.6	22	3.18	38.8	LAD-PCI	9
2015.03	a290	M	68	UA	Yes	No	1.95	2.81	5.4	190	5.35	1	70	0.18	5.6	0.4	356	3.35	11	0.95	8.19	LAD-PCI	10
2015.03	a291	F	77	ACS	Yes	Yes	1.44	3.74	7.7	228	6.58	3	70	0.69	6.7	0.3	256	1.5	11	3.93	21.3	RCA-PCI	22
2015.03	a292	M	68	ACS	No	Yes	0.89	2.71	8.9	209	12.19	1	72	4.14	8.8	0.7	299	3.12	14	1.37	14.9	LCX-PCI/LAD-PCI	31
2015.03	a293	M	88	NSTEMI	No	No	0.97	2.88	3.3	73	4.66	1	64	0.81	5.7	5.4	533	31.8	457	1.41	149	LAD-PCI	36.5
2015.03	a294	M	82	CAD	Yes	No	1.14	2.62	9.4	188	5.05	1	59	9.64	5.8	0.8	476	3.78	22	1.3	33.4	D1-PCI	21
2015.03	a295	F	82	UA	Yes	No	1.38	2.04	5.7	221	4.75	1	67.9	1.28	5.6	0.6	768	12.2	17	0.62	15.3	LAD-PCI/PTCA	16
2015.03	a296	F	67	UA	Yes	Yes	1.69	2.01	6.8	146	6	1	68.4	0.24	7.1	0.5	342	2.24	11	1.49	12.2	LAD-PCI	9
2015.03	a297	M	69	NSTEMI	No	No	1.29	1.93	5.84	193	4.82	1	97	0.26	5.6	0.5	408	2.37	8.7	1.4	7.23	LAD-PCI	33.5
2015.03	a298	F	59	UA	Yes	Yes	1.75	2.55	6	219	5.7	1		0.38	6.4	0.2	359	2.38	10	1.01	18.7	RCA-PCI	22
2015.03	a299	M	80	CAD	No	No	1.13	1.32	4.8	93	5.36	1	56	0.51	6.1	0.6	681	4.5	28	1.32	29.9	LAD-PCI	27.5
2015.04	a300	M	39	Antiqu	Yes	Yes	1.18	2.29	9.5	178	12.59	1	46	0.75	11.1	0.6	207	2.29	28	0.59	57.9	RCA-PCI	18
2015.04	a301	M	77	ACS	No	No	0.98	2.07	5.4	162	4.78	3	62	1.81	5.2	4.7	426	7	155	2.93	136	RCA-PCI	34
2015.04	a302	M	62	CAD	Yes	No	1.19	2.03	7	159	4.88	2	56	0.9	5.3	2.3	450	4.19	196	0.98	309	LM-LAD/LCX-PCI	36
2015.04	a303	M	70	CAD	No	No	1.42	2.11	6.6	255	4.86	1	72	0.48	6.1	0.2	264	1.19	16	3	12.6	RCA-PCI	10
2015.04	a304	F	67	ACS	No	No	2.2	2.42	5	192	5.77	1	64.9	2.94	5.3	<5	282	<2	7.9	<5	6.6	LAD-PCI	8

2015. 04	a305	M	68	ACS	Yes	No	1.45	3.32	6.6	176	5.81	1	68	1.35	5.5	1.6	367	8.93	59	4.63	20.1	LAD-PCI	14
2015. 04	a306	F	64	ACS	Yes	No	1.37	2.43	6.32	205	6.19	2	64.9	0.17	6.4	0.6	211	1.22	27	2.24	15.3	LAD-PCI	12
2015. 05	a307	M	70	CAD	Yes	No	0.99	2.92	5.91	146	5.39	3	58	0.55	5.4	0.5	599	1.36	9.7	0.53	19	RCA-PCI	27
2015. 05	a308	M	77	UA	Yes	No	1.24	1.81	5.9	398	4.51	1	68	0.48	4.7	1.5	335	3.66	12	1.22	26	RCA-PCI	7
2015. 05	a309	M	67	UA	Yes	Yes	1.32	3.29	5.4	118	4.62	1	67	0.68	6.1	0.5	295	2.99	21	1.39	13.9	RCA-PCI	18
2015. 05	a310	M	53	After P	No	Yes	1.07	2.27	9.9	191	7.82	2	66	0.99		5.6	476	2.95	113	1.36	61	LAD-PTCA	16

